# Information theory approaches to improve glioma diagnostic workflows in surgical neuropathology

**DOI:** 10.1111/bpa.13050

**Published:** 2022-01-10

**Authors:** Lokman Cevik, Marilyn Vazquez Landrove, Mehmet Tahir Aslan, Vasilii Khammad, Francisco Jose Garagorry Guerra, Yolanda Cabello‐Izquierdo, Wesley Wang, Jing Zhao, Aline Paixao Becker, Catherine Czeisler, Anne Costa Rendeiro, Lucas Luis Sousa Véras, Maicon Fernando Zanon, Rui Manuel Reis, Marcus de Medeiros Matsushita, Koray Ozduman, M. Necmettin Pamir, Ayca Ersen Danyeli, Thomas Pearce, Michelle Felicella, Jennifer Eschbacher, Naomi Arakaki, Horacio Martinetto, Anil Parwani, Diana L. Thomas, José Javier Otero

**Affiliations:** ^1^ Department of Pathology The Ohio State University Wexner Medical Center Columbus Ohio USA; ^2^ Mathematical Biosciences Institute The Ohio State University Columbus Ohio USA; ^3^ Peoples' Friendship University of Russia Moscow Russia; ^4^ Facultad de Medicina UdeLaR Cátedra de Anatomía Patológica, Hospital de Clínicas Manuel Quintela Universidad de la República Uruguay; ^5^ Department of Biomedical Informatics The Ohio State University College of Medicine Columbus Ohio USA; ^6^ Pathology Department Barretos Cancer Hospital Barretos Brazil; ^7^ Molecular Oncology Research Center Barretos Cancer Hospital Barretos Brazil; ^8^ Life and Health Sciences Research Institute (ICVS) School of Medicine University of Minho Braga Portugal; ^9^ MultiPat Laboratório de Anatomia Patológica Campinas Brazil; ^10^ Department of Neurosurgery Acibadem MAA University Istanbul Turkey; ^11^ Department of Pathology Acıbadem University School of Medicine Istanbul Turkey; ^12^ Division of Neuropathology Department of Pathology University of Pittsburgh Medical Center Pittsburgh Pennsylvania USA; ^13^ Division of Neuropathology Department of Pathology University of Texas Medical Branch Galveston Texas USA; ^14^ Department of Pathology Barrow Neurological Institute St. Joseph's Hospital and Medical Center Phoenix Arizona USA; ^15^ Departamento de Neuropatología y Biología Molecular Instituto de Investigaciones Neurológicas Dr Raúl Carrea (FLENI) Buenos Aires Argentina

**Keywords:** 1p/19q codeletion, cIMPACT, glioma, image segmentation, information theory, machine learning

## Abstract

**Aims:**

Resource‐strained healthcare ecosystems often struggle with the adoption of the World Health Organization (WHO) recommendations for the classification of central nervous system (CNS) tumors. The generation of robust clinical diagnostic aids and the advancement of simple solutions to inform investment strategies in surgical neuropathology would improve patient care in these settings.

**Methods:**

We used simple information theory calculations on a brain cancer simulation model and real‐world data sets to compare contributions of clinical, histologic, immunohistochemical, and molecular information. An image noise assay was generated to compare the efficiencies of different image segmentation methods in H&E and Olig2 stained images obtained from digital slides. An auto‐adjustable image analysis workflow was generated and compared with neuropathologists for p53 positivity quantification. Finally, the density of extracted features of the nuclei, p53 positivity quantification, and combined ATRX/age feature was used to generate a predictive model for 1p/19q codeletion in *IDH*‐mutant tumors.

**Results:**

Information theory calculations can be performed on open access platforms and provide significant insight into linear and nonlinear associations between diagnostic biomarkers. Age, *p53*, and *ATRX* status have significant information for the diagnosis of *IDH*‐mutant tumors. The predictive models may facilitate the reduction of false‐positive 1p/19q codeletion by fluorescence in situ hybridization (FISH) testing.

**Conclusions:**

We posit that this approach provides an improvement on the cIMPACT‐NOW workflow recommendations for *IDH*‐mutant tumors and a framework for future resource and testing allocation.

## Graphical abstract

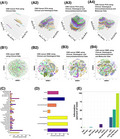

**Abbreviations**
ATRX: ATP‐dependent helicase
CCC method: Cut‐Cluster‐Classify method
CCC: concordance correlation coefficient
CGGA: Chinese Glioma Genome Atlas
cIMPACT‐NOW: the Consortium to Inform Molecular and Practical Approaches to CNS Tumor Taxonomy
CNN: convolutional neural network
CNS: central nervous system
DAB: diaminobenzidine
FISH: fluroscence in situ hybridization
GFAP: glial fibrillary acidic protein
GIMP: GNU Image Manipulation Program
H&E: hematoxylin and eosin
IDH: isocitrate dehydrogenase
IHC: immunohistochemistry
IoU: intersection over union
LMIC: low‐to‐middle‐income country
ML/AI: machine learning/artificial intelligence
PC: principal component
PCA: principal component analysis
SOP: standard operating procedure
TCGA: The Cancer Genome Atlas
tSNE: t‐distributed stochastic neighbor embedding
WHO: World Health Organization
WSI: whole slide imaging

## 1 INTRODUCTION

The integration of molecular subtyping in brain tumor diagnosis for patients in low‐resource settings represents a huge inequity in clinical care and is the premier leadership challenge facing the neuropathology community. Without a doubt, the integration of molecular data with histology has improved diagnosis and prognostication and assisted with neuro‐oncology and radiation oncology treatment planning [1]. However, the deployment of these technologies globally is fraught with caveats. Per recent reports, the United States has less than 450 formally trained neuropathologists (and fewer with molecular diagnostics experience), and the number of pathologists per capita shows a negative trajectory over time [2]. This trend is worse throughout the world. Globally, pathologist training is under‐resourced, pathology practice is poorly compensated, dedicated subspecialty training is nonexistent, and poorly implemented laboratory procedures for basic histochemical and immunohistochemical assays dampen the prospects of molecular assay deployment. Although some of the technical costs of molecular assays have declined, low‐resource healthcare ecosystems also suffer from a paucity of clinical bioinformaticists [3]. This lack of human resource investment in clinical bioinformaticists may even be a more dire threat to molecular pathology deployment than the distribution of sequencing technologies. Furthermore, as primary brain cancers are rare, brain tumor‐specific immunohistochemical biomarker testing is not commonly available even in some academic pathology laboratories. These realities directly conflict with the diagnostic recommendations put forth in the WHO Classification of CNS Tumors. Solutions that attempt to overcome the lack of investment in reagents, physical resources, and human resources are needed. For these reasons, a strong interest within neuropathology has emerged to implement machine learning/artificial intelligence‐based workflows in routine clinical practice.

Alternative solutions, including centralized diagnostic services, have been attempted. For instance, Crosier et al. reported on a centralized neuropathology review strategy for medulloblastoma in the United Kingdom which evaluated histology, RNAseq, and DNA methylation data of ~80 medulloblastoma patients per year [4]. They reported a modified risk stratification for 29% of patients and provided a critical feasibility analysis of this model. However, such a centralized review may not be feasible in all healthcare ecosystems. For instance, in the United States, DNA methylation profiling is not widely available or clinically validated in many laboratories. Expanding testing to all brain tumors is highly problematic. Chief among these challenges is the well‐known problem that diagnostic tests lose their predictive values when disease incidence decreases in the sampling population. This phenomenon most commonly occurs when increasing the number of subjects being tested without regard to pretest probability thresholds. Furthermore, in some clinical paradigms, next‐generation sequencing (NGS) has shown to be cost‐effective only at specific pretest probability thresholds [5]. Triaging tests based on the incorporation of clinical, histologic, and immunohistochemical findings have been proposed as a cost‐effective solution [6].

With these concerns in mind, we focused on generating simple, easily deployable tools for assessing the most critical investment strategies for low‐resource laboratories. To achieve this, we utilized information theory to identify the most critical data required for diagnostics and posit that simple information theory calculations can aid in determining investment strategies for biomarker acquisition.

## 2 MATERIALS AND METHODS

### 2.1 Brain cancer simulated population model

Our brain cancer population simulation is composed of 51 diagnostic entities obtained from the WHO Classification of CNS Tumors [7]. Data on these features were extracted from the scientific literature to generate a simulation of patient populations with these entities. The clinical features, the histologic features, immunoohistochemichal markers, and molecular features were delineated in [Link], [Link] with all references (summary of each entity is available in Figures S1–S51). The importance of each feature in the dignosis of this simulation model was performed by separating all the data into a training set (70%) and a test set (30%). The randomForest algorithm was called from R’s randomForest package using the default values, and the efficiency of the model was tested by evaluation of a confusion matrix by the confusionMatrix function call in R’s caret package, which showed that this model had an accuracy of 0.95 (95% CI = [0.952, 0.9564], no information rate = 0.068, and *p* < 10^−12^). We then split the dataframe by diagnosis and, for each diagnosis, passed the a random forest classifier followed on the original dataframe. For each feature of the dataframe, we scrambled each diagnostic feature by performing a random sampling of the original dataframe containing all diagnoses and saved the value of the accuracy generated by random forest classifier. These data are presented in Table S1.

### 2.2 Real‐world data sets for information theory validation

Nine different data sets from different countries were used for the calculations of Bayesian probabilities and mutual information. The numbers of available cases and details were delineated in Table 1.

**TABLE 1:** Summary of clinical data for epidemiologic study

	Country	Number of available cases (Astrocytoma/oligodendroglioma)		
		For age	For gender	For information theory
The Cancer Genome Atlas (TCGA)	The United States, Brazil, Italy, Germany, Australia	411 (243/168)	411 (243/168)	411 (243/168)
Chinese Glioma Genome Atlas (CGGA)	China	88 (48/40)	88 (48/40)	88 (48/40)
The Ohio State University (OSU)	The United States	110 (59/51)	110 (59/51)	110 (59/51)
FLENI	Argentina	276 (166/110)	276 (166/110)	76 (54/22)
Hospital de Amor, Barretos	Brazil	37 (20/17)	37 (20/17)	26 (13/13)
University of Texas Medical Branch (UTMB)	The United States	32 (18/14)	–	–
Barrow Neurological Institute	The United States	36 (14/22)	–	–
Acibadem Hospital	Turkey	193 (82/111)	193 (82/111)	189 (79/110)
University of Pittsburgh Medical Center (UPMC)	The United States	207 (121/86)	207 (121/86)	207 (121/86)
Total		1390 (771/619)	1322 (739/583)	1107 (617/490)

### 2.3 Age distribution and probability calculation

Age distribution of *IDH1/2*‐mutant gliomas was presented as density plots. For the Bayesian probability plots, probabilities of 1p/19q codeletion in *IDH1/2* mutant tumors given age and gender were calculated using the “naivebayes” package in R with a Laplace correction for smoothing.

### 2.4 Information theory calculations

**FIGURE 1:**
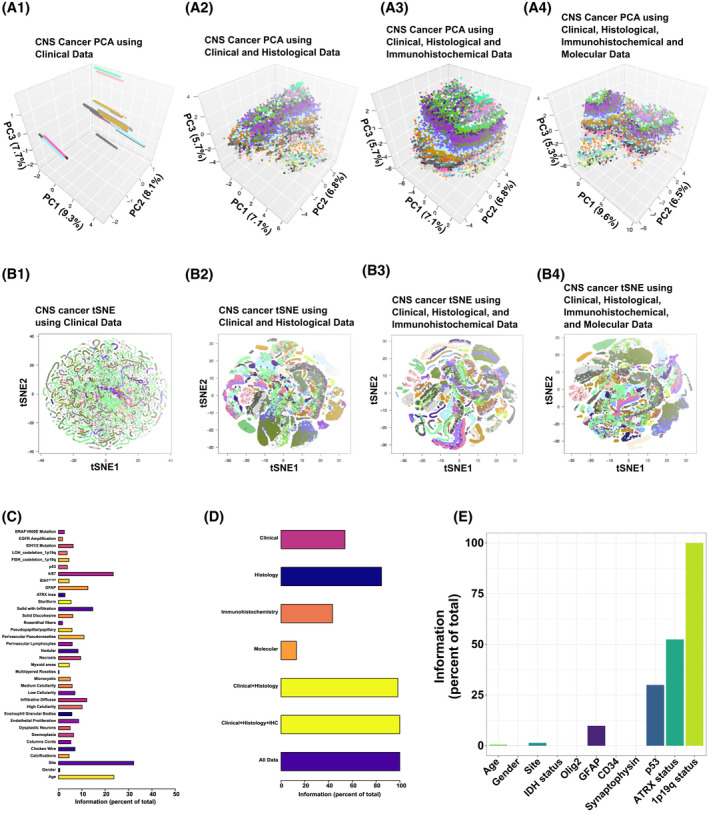
Different clustering patterns with clinical, histologic, immunohistochemical, and molecular information on the brain cancer population simulation and information gain in the glioma simulation model for clinical decision‐making. Dimensionality reduction by principal component analysis is shown in A1–A4, and by *t*‐stochastic neighbor embedding in B1–B4, with the features delineated on the top of each graph. Each color represents a unique diagnosis in the WHO classification scheme. (A1 and B1) Dimensionality reduction with clinical features alone demonstrates only a few visible clusters. (A2 and B2) Incorporating clinical history with histology generates the commencement of clear layering in PCA and discrete clusters with t‐SNE. (A3 and B3) Inclusion of immunohistochemical data improves the capacity of discerning clusters. (A4 and B4) The additional molecular features does not significantly improve the clustering. (C) Individual information gains with all clinical, histologic, immunohistochemical, and molecular features in the glioma simulation model. Age, site, and Ki67 are the features that have the most amount of necessary information for the diagnosis. (D) Information gains with clinical, histologic, immunohistochemical, and molecular information. Histology provides most of the necessary information for the diagnosis. Combining the clinical data with histology and immunohistochemistry provides more than 95% of the necessary information, whereas adding molecular data provides minimal information gain. (E) *IDH1/2*‐mutated tumors were subsetted, and the mutual information of the features on the *X*‐axis was quantified as % information on the *Y*‐axis. The conditional information function call was called on data the 1p19q status

**FIGURE 2:**
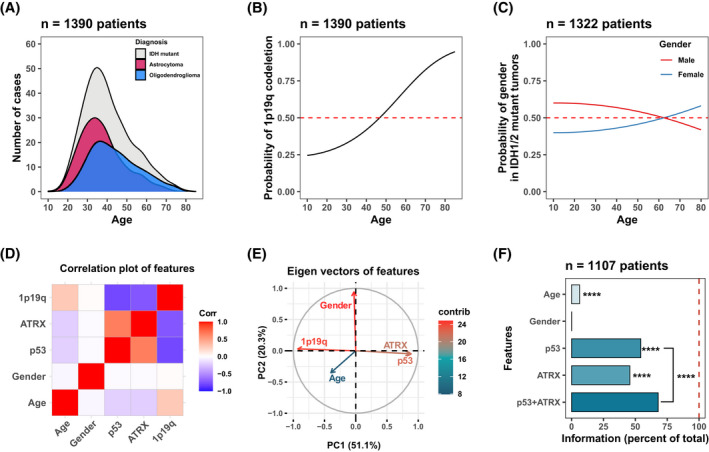
A global IDH‐mutant (Grades 2 and 3) data set was used for the application of information theory as real‐world data and the importance of selected features for 1p19q codeletion status. (A) IDH‐mutant astrocytoma and oligodendroglioma cases have a different distribution of age. Oligodendroglioma cases are more common in advanced age. (B) Probability of being an oligodendroglioma increases with age. (C) There is not a significant difference in the gender for the probability of 1p/19q codeletion. (D) Correlation plot of features as a dummy variable. p53 and ATRX mutation have a strong correlation and negative correlation with 1p19q codeletion. Gender does not show any correlation with other features including 1p19q codeletion. (E) Dimensionality reduction of the features with a PCA shows similar results to the correlation matrix, which is similar variation between the p53 and ATRX mutation. (F) p53 and ATRX mutation information provide significant information for the diagnosis. Adding ATRX to p53 mutation provides additional information even though there is marked redundancy between these features. Permutation analysis was used for significance analysis. *p ≤ 0.05, **p ≤ 0.01, ***p ≤ 0.001; ****p ≤ 0.0001

Information theory, initially developed by Claude E. Shannon at Bell Labs in 1948 [8], rests on the notion that information can be objectively quantified into units called bits (or nats, where 1 nat =log2(e)*bit). Mutual information is the exact opposite term of entropy, where entropy represents the uncertainty of the information. Maximum mutual information indicates all information quantity needed for the 1p/19q codeletion status (or diagnosis) in our example. For simplicity, the provided information with features is represented as a percentage of maximum mutual information (as in Figures 1, 2, and 5). Details were delineated in [Link], [Link].

### 2.5 Image acquisition

Hematoxylin–eosin (H&E)‐stained sections and p53 and Olig2 immunohistochemistry‐stained sections of *IDH*‐mutant diffuse gliomas were selected for image segmentation. Details were delineated in [Link], [Link].

### 2.6 Segmentation methods (unsupervised and supervised) and evaluation of segmentation fidelity

Two types of segmentation methods were used in our analysis: unsupervised (Deconvolution combined with Otsu thresholding, K‐means, and the Cut‐Cluster‐Classify) and supervised methods (Trainable Weka Segmentation and U‐Net). We made a ground truth using GIMP and R for the evaluation of segmentation fidelity and U‐Net training. Details were delineated in [Link], [Link].

### 2.7 Evaluation of segmentation fidelity

For our evaluation, we only have two regions of interest: cell nuclei (stained in purple for H&E images and brown for Olig2 and p53 images) and background. Since one of the challenges of segmenting our data set comes from the various levels of noise, we also developed an image noise assay to test how robust the methods are to noise. Percentage ratios of added noise were selected as 0, 5, 10 15, 25, 50, 75, 90 to evaluate the whole pattern of noise resistance. We report accuracy and intersection over union (IoU). Details were delineated in [Link], [Link].

### 2.8 Data processing

#### 2.8.1 Condensation of data and probability calculation of Olig2 features

Eighty‐nine features were extracted from the nuclei in each channel of the images using the masks after the segmentation with the deconvolution method. After feature extraction, we excluded incomplete nuclei in the images for a better representation. Extracted features are shape, moment, pixel intensity, and Haralick's features. We had 213 features left after the elimination of repeated features in different channels. After principal component analysis (PCA) application, 8 principal components (PCs) in Olig2 features and 9 principal components (PCs) in H&E features were selected for the representation of 90% variation of features. We calculated the sample density of nuclei in a multidimensional space of PCs. Three hundred and fifty nuclei were sampled in each case for density calculation of features extracted from H&E‐ and Olig2‐stained images. The mean, median, and standard deviation values of the distances to 10th (d10), 20th (d20), 40th (d40), and 60th (d60) closest nuclei were calculated in a distance map as “density features.” The histogram parameters, such as mean, standard deviation, kurtosis, and skewness, on selected 3 shape features (major axis, eccentricity, and area) were used as “histogram features.” The mutual information of these density and histogram features of H&E‐ and Olig2‐stained slides were used for feature selection.

#### 2.8.2 Automated p53 positivity workflow

An automated positivity workflow for p53 immunoreactivity was developed for objective quantification. The detailed workflow was explained in the Results section. Briefly, we did segmentation on 1 image from positive control, 1 image from negative control, and 4 images from the tumor on the slide. After feature extraction, we labeled nuclei in the positive and negative control and used these labels as ground truth to make an RF modeling for the prediction of nuclei in the tumor images. After prediction, the positivity percentage was calculated as a percentage of positive nuclei to the total. We compared the automated positivity workflow results of p53 with human observers’ evaluations. These human observers were practicing neuropathologists at different hospitals who utilized slightly distinct interpretation workflows for p53 immunohistochemistry. Each neuropathologist interpreted the p53 immunohistochemistry as indicative of “mutant” or “wild type” in 82 cases of p53 images. After completion, the same 74 cases (8 cases do not have ATRX images) were evaluated with ATRX images to detect any bias of ATRX information on p53 positivity results. For models utilized in Figure 6, the continuous scale %p53 positive cells obtained from our automated workflow was utilized.

## 3 RESULTS

### 3.1 Information theory enhances understanding of individual feature impact in diagnostic neuropathology

Our ultimate goal is to generate tools and workflows that improve pathologist decision‐making throughout the world, with a particular emphasis on resource‐poor settings. To this end, we generated a brain cancer population simulation, whereby we obtained specific features known to neuropathologists who are capable of distinguishing tumors and performed a thorough review of the scientific literature to obtain the data upon which to build this simulation (see Supporting Materials and Methods). We utilized three easily obtainable clinical features (age, neuroanatomical site, and gender), histologic features, and immunohistochemical stains (GFAP, ATRX, Ki67, IDH1 R132H, P53, CD34, EMA, Olig2, reticulin, and synaptophysin). We also utilized the results of basic molecular assays that are commonly reimbursed by private insurers, public aid, and Medicare, in the United States. All features for each entity simulated are delineated in the Supporting Materials and Methods. We first performed dimensionality reduction using principal component analysis (PCA) as well as a nonlinear algorithm, the tSNE, on our simulated brain tumor population (Figure 1A,B). We note that plotting tumors only on clinical features (age, site, and gender) with tSNE and PCA results in no discernable cluster generation (Figure 1A1,B1 **)**. By including histologic features from H&E‐stained sections, one is able to visibly appreciate the generation of specific clusters of entities (Figure 1A2,B2). The addition of immunohistochemistry further increased the clusters relative to clinical features plus immunohistochemistry. However, the addition of molecular features did not necessarily increase the clustering upon visual inspection (compared Figure 1A4,B4 with Figure 1A3,B3). These data are in‐line with well‐known concepts in the neuropathology community that emphasize the integration of clinical context with histology. We further conclude that this simulation provides a useful in silico framework to test workflow concepts by Monte Carlo‐like simulations prior to real‐life deployment.

Then we generated a thought experiment where we imagined a scenario where the biopsy/resection procedure represented a conduit of data transmission from the patient to the pathologist. This scenario is analogous to the flow of information in information processing and amenable to information theory calculations (see Section 2). We evaluated all the features delineated in our brain cancer simulation model and quantified the mutual information (Figure 1C). We note that age, neuroanatomical site, and Ki67 proliferation index were the features that carried the most amount of information in the glioma simulation model. A combination of individual features showed that histology data provided nearly 80% of the information necessary for diagnosis (Figure 1D). Surprisingly, basic clinical information (age, site, and gender) provided just over half of all information necessary for a diagnosis, whereas molecular data alone provided less than 20% of the information needed. Combining clinical data with histology data and immunohistochemistry increased the information to over 95%, with only minimal information gained by the molecular data. To identify the most critically important features for each diagnosis in our simulation, we generated a randomForest based workflow that identified the most critical diagnostic features in this simulation (Table S1). After evaluating the information theory calculations and the results of our randomForest analysis, we note a significant asymmetry in the type of information contained in patient data, with the neuroanatomical site, age, and Ki67 labeling index being the most important features in diagnosis. This asymmetry produces a significant tendency to generate bias in machine learning‐based diagnostic algorithms. In order to illustrate how such simulations can be used for strategic planning, we subsetted the simulated brain cancer population for IDH‐mutated tumors and performed information theory calculations to determine the utility of age, gender, site, IDH status, 1p19q status, and immunohistochemical detection of Olig2, GFAP, CD34, synaptophysin, p53, and ATRX (Figure 1E). We noted that p53 and ATRX status provided a significant quantity of information as to 1p19q status. We further conclude that basic information theory calculations permit the rapid acquisition of which features are most impactful for diagnostic neuropathology.

### 3.2 Age, p53, and ATRX status have significant contribution in the diagnosis of *IDH*‐mutant tumors

Then we focused on *IDH*‐mutant tumors to validate these information theory‐focused workflows. To achieve this, we took advantage of publicly available data sets from the Cancer Genome Atlas and the Chinese Glioma Genome Atlas as well as data from hospitals in Turkey, the United States, Argentina, and Brazil to provide a global perspective (see map in Figure S52). The data sets collected included patient age (continuous numeric), gender (binary), p53 (binary), ATRX (binary), and 1p/19q codeletion status (binary), summing to 1390 total *IDH*‐mutant gliomas. We noticed that *IDH*‐mutant gliomas showed a different distribution of patient age. Generating a probability distribution of diagnosis as a function of age, we noticed an increase in the probability of 1p/19q codeletion with advanced age (Figure 2A,B). Confirming other studies, we found no association of gender with 1p/19q codeletion status (Figure 2C).

We then evaluated the relationship of these biomarkers to 1p/19q codeletion status by two linear methodologies: correlation matrix and principal component analysis (PCA). To achieve this, we generated dummy variables of the features. Figure 2D shows that ATRX and p53 mutations show a high correlation with each other and, as expected, are highly negatively correlated with 1p/19q codeletion status. Then we interrogated the data variance by PCA. As shown in Figure 2E, PC1 and PC2 account for over 70% of the variance in the data set. By plotting the eigenvectors along PC1 and PC2, we can appreciate the significant redundancy between ATRX and p53 status in the PC1/2 graph (note the near superimposition of the ATRX and p53 eigenvectors in Figure 2E). Also, the eigenvector for gender shows a nearly orthogonal orientation relative to 1p/19q codeletion eigenvector, indicating no association. These evaluations raise the possibility that data for P53 and ATRX show significant redundancy, and if so, assessing both biomarkers would be unnecessary in diagnostic workflows. To test this hypothesis, we performed information theory calculations of these parameters (Figure 2F). We found a statistically significant increase in information when both ATRX and p53 were evaluated together, a finding in‐line with current cIMPACT‐NOW clinical practice [9]. This means new information gain is present when combining both p53 and ATRX data points. We conclude that information theory analysis is capable of determining if biomarkers showing significant correlation and covariance add significant information when used in combination. It also provides significant additional insight into linear computational methods.

### 3.3 Auto‐adjustable image analysis workflows for surgical neuropathology

We next sought to evaluate the information gain extractable from tissue morphology. A well‐appreciated morphologic distinction between *IDH*‐mutant gliomas is the propensity of the former to show significantly greater nuclear pleomorphism. We first evaluated Olig2 expression by immunohistochemistry, a biomarker diffusely expressed in infiltrating gliomas [10, 11, 12], and which was a commonly utilized biomarker among our global collaborative group. Olig2’s nuclear localization also facilitates image segmentation. We then evaluated a variety of supervised and unsupervised segmentation algorithms, which included (1) matrix deconvolution with OTSU thresholding [13, 14], (2) WEKA segmentation [15], (3) k‐means segmentation, (4) Cut‐Cluster‐Classify segmentation [16], and (5) a convolutional neural network with a UNET architecture [17]. We also performed a separate analysis utilizing a UNET algorithm trained with experimental noise (see Figure S57 for Image Noise Assay workflow and comparison of segmentation methods). Our data were derived from the OSU patient archives. We note that matrix deconvolution with OTSU thresholding showed high accuracy up until 25% noise for both H&E staining and Olig2 immunohistochemistry (Figure S57B,C, blue line), whereas UNET trained with significant noise was able to maintain high accuracy with high levels of noise. However, when evaluating IoU, both methods showed significant drop‐off with more than 25% noise (Figure S57B,C). We conclude that for simple segmentation processes where the high signal to noise exist, matrix deconvolution with OTSU thresholding is the simplest approach.

**FIGURE 3:**
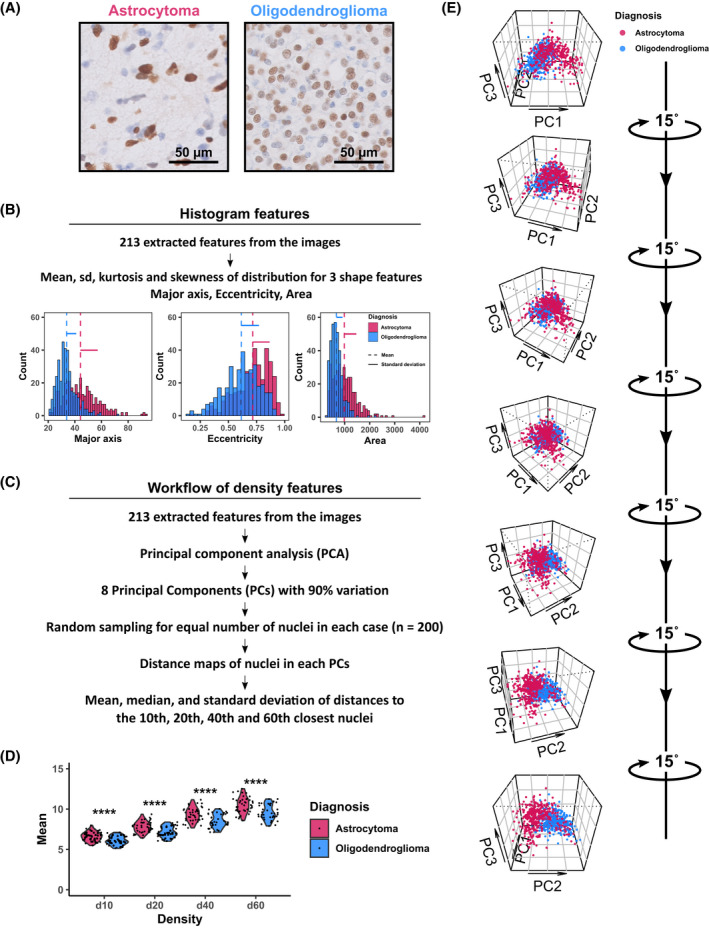
Explanation of density and histogram features. (A) Typical astrocytoma and oligodendroglioma images. These cases are used for the explanation of histogram and density features. (B) The histogram of 3 shape features (major axis, eccentricity, and area) in example images were compared. Astrocytoma and oligodendroglioma show different means, standard deviations, and histogram shapes which support different kurtosis and skewness. (C) Two hundred and thirteen extracted features per nucleus were used for the calculation of density features. After dimensionality reduction with PCA, we utilized 8 PCs, which accounted for 90% variation. PCA also provided us perpendicular features in multidimensional space for Euclidean distance calculation. The same number of nuclei were sampled before distance calculation to avoid the effect of cellularity in the images. The mean, median, and standard deviation of Euclidean distance of nuclei to its 10th, 20th, 40^th^, and 60th closest neighbor in the multidimensional space were used as density features. (D) Comparison of mean d10, d20, d40, and d60 between astrocytoma and oligodendroglioma cases. (E) Our oligodendroglioma and astrocytoma examples have different distributions of nuclei in a 3D space of PCs. Although astrocytoma example has a wide‐spread distribution of nuclei in multidimensional space, oligodendroglioma nuclei are highly clustered in one location that supports the finding of more uniform nuclei in oligodendroglioma

We then generated image masks of all Olig2‐stained nuclei and overlaid the image masks onto the red, green, and blue image matrices to extract features of cell nuclei. These features included shape, pixel intensity, and pixel texture representing over 200 features per nucleus. To reduce the dimensions, we performed PCA which provided 90% of the variance in 8 principal components for Olig2 staining. Evaluation of morphologic features in some cases showed a tendency for oligodendroglioma cases to show higher kurtosis in histograms describing nuclear shape features (Figure 3B). However, the absolute values of these parameters differed on a case‐by‐case basis, and we were not able to use these raw measurements. We also felt that the pleomorphism among cells likely also included marked variance in DAB‐staining intensity and texture (e.g., compare astrocytoma staining pattern for Olig2 in Figure 3A). We, therefore, evaluated the principal components in multidimensional space (Figure 3C shows the workflow). An example of our analysis is shown in Figure 3D for a sample case of *IDH*‐mutant astrocytoma and oligodendroglioma. Note that the *IDH*‐mutant astrocytoma shows a wide scatter of coordinates, thus showing a lower data density. In contrast, oligodendroglioma is more heavily clustered in one area. This dense packing of the oligodendroglioma nuclei represents a reflection of the nuclear monomorphism in oligodendroglioma relative to astrocytoma. We calculated the mean Euclidean distance of each cell to its 10th, 20th, 40th, and 60th neighbor in the multidimensional PC space for each patient in our data set. An increased Euclidean distance to its *n*th neighbor would indicate that, on average, larger heterogeneity in morphology, Olig2 intensity, and Olig2 texture is present in tumor cell nuclei. For each distance measure, we note that *IDH*‐mutant astrocytoma showed elevated Euclidean distance relative to oligodendroglioma, indicating a lower density in multidimensional space (Figure 3E). Data density calculations from Euclidean Distance capture the nuclear pleomorphism differences between *IDH*‐mutant gliomas. We further posit that this methodology, which is based on within‐sample differences, represents a superior metric for evaluating morphology as it would be more resistant to interlaboratory methodologies.

**FIGURE 4:**
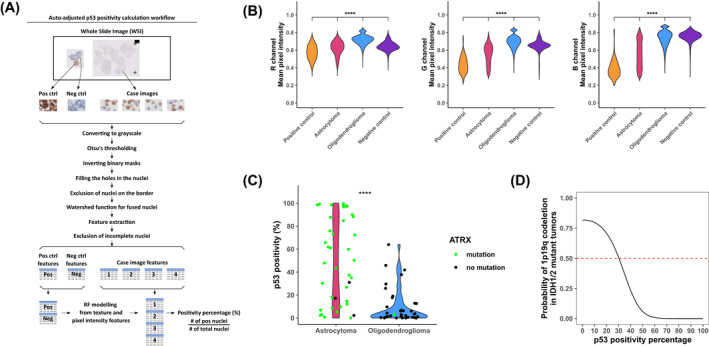
Auto‐adjusted p53 positivity calculation workflow and relationship between p53 continuous scale and 1p/19q codeletion status. (A) We took 3 types of images from p53‐stained whole slide images: positive control (ovarian serous carcinoma), negative control (benign lymph node), and glioma images. Texture and pixel intensity features of positive and negative control nuclei were used for modeling. All nuclei in the glioma images were labeled by the model as positive and negative. p53 positivity percentage was calculated as the number of positive nuclei divided by the number of all nuclei in the images. (B) Comparison of mean pixel intensity values in R, G, and B channels of tumor images, positive, and negative controls. (C) Distribution of p53 positivity on astrocytoma and oligodendroglioma cases. Astrocytoma cases have a more uniform distribution for p53 positivity although oligodendroglioma cases mostly have a very low p53 positivity percentage. Green and black colors show the ATRX mutation status of cases. (D) Trend of the probability of 1p19q codeletion status with p53 positivity percentage obtained from this dataset. *p ≤ 0.05, **p ≤ 0.01, ***p ≤ 0.001, ****p ≤ 0.0001

**FIGURE 5:**
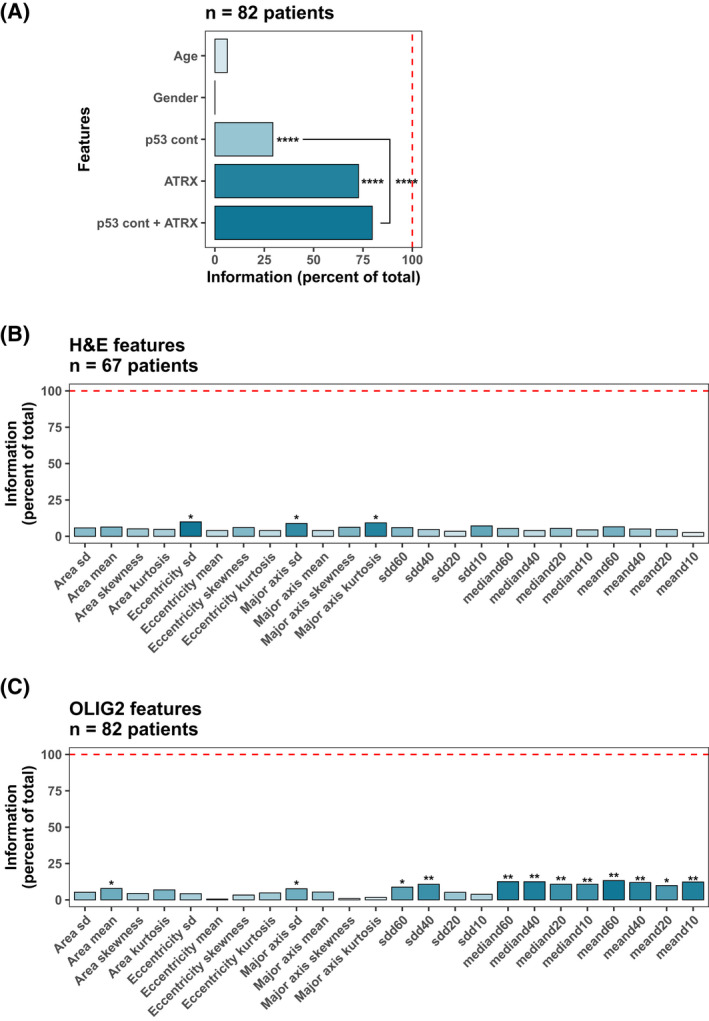
Information theory of features (age, gender, p53 continuous scale, and ATRX) on the OSU data set and significance of H&E and OLIG2 features for diagnosis of IDH‐mutant Gliomas. (A) Comparison of information gained on 82 cases. p53 positivity percentage from automated workflow and binary ATRX mutation results from the reports show the highest significant information gain for the diagnosis. Adding ATRX information to the p53 positivity percentage provides significant information although there is a big redundancy between them. (B and C) 10 Olig2 features provide significant information for the diagnosis although there is only 1 H&E feature that provides significant information. These 10 significant Olig2 features were used for modeling in later applications

We next focused our analysis on features of p53 immunohistochemistry, an important biomarker for glioma classification. Intense p53 nuclear immunoreactivity is utilized as a surrogate for *TP53* mutation but suffers significant limitations. In addition to some inactivating *TP53* mutations resulting in complete negative staining [18, 19], Takami, et al. demonstrated that in addition to immunoreactivity intensity, percent of immunoreactivity was also a critical feature [20]. However, other studies have set a cutoff of positivity for p53 positivity by IHC as 10% regardless of intensity in other tumor paradigms [21]. Generating a universal intensity and texture cutoff for p53 immunohistochemistry is challenging due to variance in laboratory practices. In our institution, we routinely perform our glioma‐related p53 immunohistochemistry with a serous carcinoma positive control and a benign lymph node as a negative control, mounted on the same slide. We utilized these internal controls as our metric for the designation of p53 positive or negative for each case (see the workflow in Figure 4A). The mean accuracies of random forests for each case are 0.991 (95% CI: 0.985–0.996). Once the model is trained, we passed it on to the actual data from the patient to generate the prediction. For benchmarking, we compared the performance of our model to two human neuropathologists (see Table 2 for concordance data). We note that Lin's concordance for interobserver neuropathology recordings showed overlap in the 95% CI of neuropathology observer 1: automated workflow and neuropathology and observer 2: automated workflow. These data indicate that the automated workflow shows similar a concordance level to neuropathologists as neuropathologists concord with each other. We next tested the extent to which mean pixel intensity extracted from the nuclei were distinct between the astrocytoma and oligodendroglioma cases in the red, green, and blue channels (Figure 4B). Mean pixel intensity distribution showed a clear distinction between the tumor nuclei and control nuclei for the modeling. Figure 4C demonstrates the distribution of p53 positive cells, as determined by our random forest classifier for each case. The variance between the p53 quantification is quite large in the astrocytoma cases, whereas in the oligodendroglioma, the majority of the cases showed low %p53 detection. Based on these data, we then generated a probability function using the naïve Bayes function in R (Figure 4D). As %p53 positivity increases, the probability of 1p/19q codeletion in our data set decreases in an exponential manner. Figure 5A demonstrates information theory calculations for this p53 continuous‐scale data set. Automated p53 quantification using this workflow represents an objective alternative to subjective p53 interpretation. Also, the addition of ATRX information to p53 positivity percentage provides a significant information increase for diagnosis. We also used a mutual information approach to determine the significant features in the histogram and density features of H&E‐ and Olig2‐stained images (Figure 5B,C). There are 12 significant Olig2 features (Figure 5C), whereas 3 significant H&E features were noted (Figure 5B).

**TABLE 2:** Comparison of p53 scoring systems

Comparison	CCC	95% CI
Observers–Workflow comparison		
Observer 1 vs. Workflow	0.60	0.46–0.72
Observer 2 vs. Workflow	0.56	0.44–0.67
Observer 1 vs. Observer 2 vs. Workflow	0.63	
Observer–Observer with ATRX comparison		
Observer 1 vs. Observer 1 with ATRX	0.66	0.51–0.77
Observer 2 vs. Observer 2 with ATRX	0.93	0.90–0.95
Observer 1–Observer 2 comparison		
Observer 1 vs. Observer 2	0.78	0.70–0.85
Observer 1 with ATRX vs. Observer 2 with ATRX	0.73	0.61–0.82

### 3.4 Informatics‐based clinical practice guidelines for the evaluation of *IDH*‐mutant tumors

**FIGURE 6:**
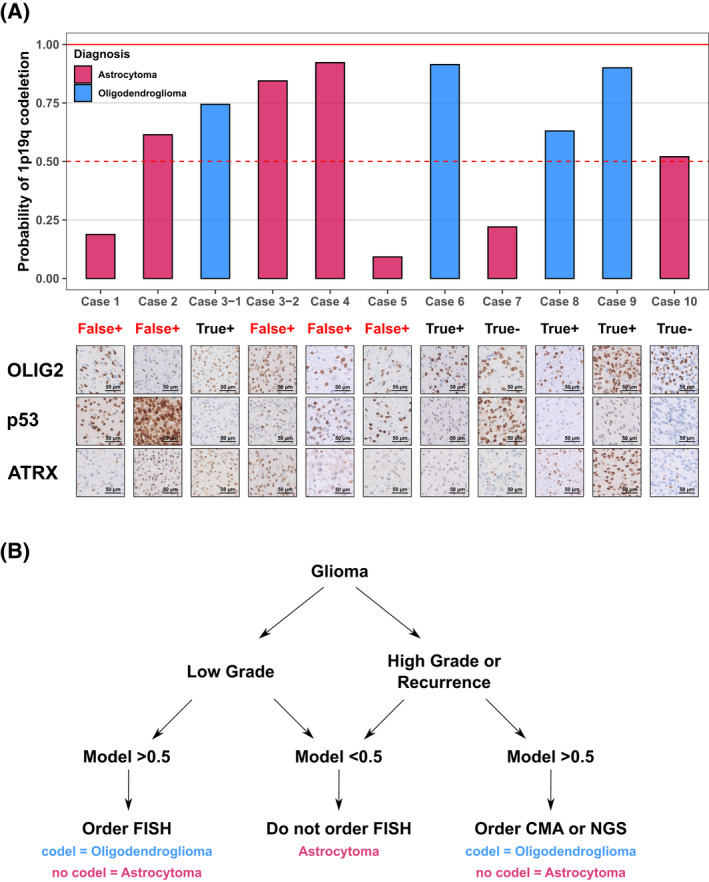
Prediction results of validation data set using p53 positivity percentage, Olig2 features, and the probabilities based on age and ATRX status. (A) Prediction results on validation cases. All predicted astrocytoma cases are true and the model captured all oligodendroglioma cases. All failed cases (Case 2, Case 3–2, and Case 4) are high‐grade (Grade 4) glioma cases. The other cases are grade 2/3 glioma cases. We generated an algorithm based on the prediction results and tumor grades. Details of the algorithm are delineated in the results. The representative images for Olig2, p53, and ATRX immunohistochemistry were shown in the figure. (B) Algorithm for when to order FISH, chromosomal microarray (CMA), or next‐generation sequencing (NGS) for 1p19q codeletion status. The first step in the algorithm is the grade and the recurrence. If the prediction result is less than 50%, there is no need to order FISH, CMA, or NGS. If the prediction is more than 50% and the glioma is low grade, order FISH for 1p/19q codeletion. If the prediction is more than 50% and the glioma is low grade or recurrent, order CMA or NGS for 1p/19q codeletion

We next trained a random forest classifier that used the significant Olig2 features of data density, p53 labeling (continuous scale quantification), and a combined ATRX/age feature on 82 cases. The ATRX/age feature was obtained from probability estimates of a random forest classifier trained on 1107 cases of the global data set after splitting the train (70%) and test (30%) sets, where 1p/19q codel status was modeled as a function of ATRX and patient age. This ATRX/age feature was calculated on the 82 cases of the OSU data. We note that in our OSU archival data set, missing (3 cases) or equivocal (2 cases, 1 on 82 cases, 1 on validation data set) ATRX status existed, and so we imputed the ATRX status based on a random forest classifier trained on the global data set, where ATRX status was modeled as a function of age and p53 status. For our 82 cases, we used an 80% threshold to convert the p53 continuous scale to a binary result (positive or negative) for this prediction. Then we tested the random forest classifier on the testing set (~30%, representing 25 cases). Our results showed an accuracy of 0.92%, *p* = 0.000022. We then implemented this algorithm on a series of 11 diagnostically challenging validation cases (Figure 6A and summarized in Table S2), most of which represented tumor recurrences. Detailed clinical histories of these patients are delineated in the [Link], [Link]. Our model incorrectly classified four astrocytoma cases (Case 2, Case 3‐2, Case 4, and Case 10). All the incorrectly classified cases showed either high‐grade architectural features (necrosis and microvascular hyperplasia) or represented recurrent cases. Based on these results, we propose a clinical practice guideline to facilitate decisions related to the ordering of expensive nonreimbursable modern molecular tests (Figure 6B). We note that our algorithm is quite robust for low‐grade cases. In the setting of a low‐grade *IDH*‐mutant tumor, if our model prediction is less than 0.5 for 1p/19q codeletion, the case can be easily diagnosed as an astrocytoma without further workup. If the low‐grade glioma shows a prediction of >0.5, 1p/19q codeletion by FISH is a sufficient diagnostic test to diagnose accurately *IDH*‐mutant astrocytoma and oligodendroglioma. For *IDH*‐mutant tumors that are recurrent or show high‐grade morphology, a model prediction of >0.5 should trigger a chromosomal microarray or NGS‐based 1p/19q codeletion assay so as to avoid the potential of 1p/19q FISH false positives. 1p/19q FISH false positives are the instances in which there is a discordance between 1p19q FISH and 1p19q analysis by chromosomal microarray or next‐generation sequencing as false‐positive cases with regard to the diagnosis of oligodendroglioma.

## 4 DISCUSSION

In this study, we implemented, for the first time, information theory approaches as a conceptual and practical framework upon which to determine the features that offer the most information to reach an integrated neuropathologic diagnosis. We tested this hypothesis first with a highly comprehensive simulation of brain cancer patients and validated this approach using real‐world data. We developed, validated, and deployed a simple image analysis workflow that could be adopted in resource‐poor settings using open‐source software as well as translated to a variety of diagnostic applications and proposed a clinical practice guideline recommendation in the evaluation of *IDH*‐mutant tumors. We note that few emerging concepts in modern pathology have experienced as much hype as machine learning and AI (for instance, see Ref. [22] and related its editorial Ref. [23]). We propose that our information theory approach represents a simple way for resource‐challenged healthcare ecosystems to make informed decisions on reagent and testing platform investments. We further propose that in silico simulation, such as performed by our glioma population simulation, provides an inexpensive framework amenable to Monte Carlo‐like simulation and permits the testing of how specific datapoints may affect the differential diagnosis. We posit that generating such Monte Carlo‐like simulations can provide multiple insights to other surgical pathology paradigms. The workflow presented in the [Link], [Link] can easily be substituted by other tumor types and/or other types of information by creating an *m × n* matrix in Excel with each row corresponding to a specific tumor entity, each column a feature/biomarker, and each cell a proportion of cases showing positivity for the designated feature/biomarker. Such a table would then be imported into Rstudio or Python to generate the simulation using a sampling function as delineated in the methods.

### 4.1 Implications of information theory on diagnostic neuropathology

The glioma diagnosis simulation and our information theory analysis raise some very interesting points regarding the future development and deployment of clinical diagnostic aids for neuropathology. At the forefront of our findings is the identification that simple information provides the majority of information. Specifically, basic clinical history, radiographic documentation of tumor site, and histologic findings carried the most information. This is rather intuitive, but these findings are of utmost importance for low‐resource healthcare ecosystems. These findings are in‐line with recommendations for low‐to‐middle‐income countries (LMIC) that have emphasized local healthcare ecosystems to embrace a culture of quality in pathology laboratories, in particular, by adopting appropriate standard operating procedures, quality control, and quality assurance practices [24]. Underscoring this point, a comprehensive study by Fleming et al. generated a recommended capabilities list for Pathology Laboratories in LMICs. Chief among these recommendations included high‐quality histologic/cytologic services as well as training for laboratory staff and physician‐pathologists [25, 26]. Our findings from the brain cancer population simulation underscore the key importance of quality histology in diagnostic neuropathology. Building upon these recommendations, we would further advocate that evaluation of clinical, histologic, and biomarker features by information theory could provide a quantitative framework from which to make data‐driven decisions on diagnostic services.

Our work raises significant concerns with ML‐/AI‐based clinical diagnostic aids in neuropathology. First, most brain cancer data set structures include high‐resolution genomic data on very few samples. Pooling data sets from various centers results in missing data elements that are often beyond the capabilities of imputation for clinical outcomes research [27]. In addition, as demonstrated in our glioma simulation, the quantity of information present is highly asymmetrical, and many brain tumor entities are exceedingly rare. This results in ML‐/AI‐based algorithms, nearly all of which utilize some version of information theory, to focus on age, neuroanatomical site, and proliferative index for diagnosis. As a result, predictive algorithms may generate adequate differential diagnoses, but these would be of minimal utility in routine practice. Our approach to overcoming this problem is to identify specific, granular diagnostic conundrums as candidate branch points in clinical decision‐making, where ML/AI can make an impact. This permits the focused evaluation of biomarker information with several advantages over traditional analyses. PCA and correlation plots indicated such a high correlation and covariance between p53 and ATRX status that it raised the possibility that these two biomarkers were fully redundant, legitimately questioning employing both antibodies. In contrast, using an information theory approach, we found that the incorporation of both biomarkers simultaneously resulted in a statistically significant information gain. This synergy in information gain was masked using traditional linear analyses. Similar evaluations could be employed to evaluate additional frequently paired biomarkers.

### 4.2 Simple image analysis workflows deployable throughout the world

The advent of CNN networks and image recognition has revolutionized the modern world, and it will continue to provide significant impacts to diagnostic pathology. However, its deployment in the practice of pathology globally has several limitations. First and foremost, although pathologists worldwide have access to brightfield microscopes equipped with cameras, few laboratories contain whole slide imaging (WSI)‐capable scanners. In addition to capturing all of the tissue examined, WSI has the added benefit of imposing a standard operating procedure (SOP) in image acquisition, whereas traditional photomicroscopy is nearly impossible to be replicated from one session to another. In our study, we utilized tiles of image patches taken from WSI images to mimic pathologists capturing images on their desktop microscopes. A second challenge is that the lack of adherence to SOPs for histopathology across the globe results in highly variable interlaboratory fixation and staining protocols [28]. Although several attempts to correct this have been published (e.g., Ref. [29]), the nonlinearity in histologic color maps has posed a significant challenge when controlling for fixation and staining conditions across labs [30] and may be unsurmountable in labs with high batch variance in staining. For this reason, we focused on incorporating self‐normalizing workflows when dealing with color intensity and color texture features. One simple approach is to use internal known positive controls, such as p53 immunohistochemistry in ovarian serous carcinoma [31] with a known negative control (in our case, a benign lymph node), and train within each case an algorithm based on pixel intensity and texture features. This internal slide control provides an unbiased methodology. A second approach we took was using the concept of data density within multidimensional space for the evaluation of nuclear pleomorphism in glioma. These analyses can be performed across histopathology labs as they would only require image acquisition using the same objective magnification and numerical aperture.

### 4.3 Machine learning‐workflow improvement to WHO and cIMPACT‐NOW clinical guidelines

Our test‐case scenario dealt with the need to confirm 1p/19q codeletion status in *IDH*‐mutant gliomas. In many healthcare ecosystems, even in the United States, no reimbursement for chromosomal microarray to confirm 1p/19q codeletion exists. In contrast, 1p/19q codeletion testing by FISH, although suffering in positive predictive value relative to more advanced techniques, reimburses routinely. This leads to a situation in which pathologists must make a decision to perform unreimbursed reflex testing in a serial fashion to confirm 1p/19q codeletion, further delaying treatment planning by neuro‐oncology/radiation oncology. The cIMPACT guidelines, which are anticipated to be incorporated into the 2021 WHO classification, represent a significant improvement to prior recommendations [9, 32]. Specific to our test case at hand is that current cIMPACT guidelines dictate that documentation of *TP53* mutation and concurrent *ATRX* mutation within an *IDH*‐mutant tumor excludes the diagnosis of oligodendroglioma. A specific problem arises when pathologists identify an *IDH*‐mutant tumor, and either ATRX immunohistochemistry is unavailable, or ATRX immunohistochemistry is equivocal (as was shown in Case 4 of Figure 6A) or negative due to copy number loss involving the ATRX locus in a tumor with 1p/19q codeletion. Furthermore, the designation of *TP53* mutation subjectively by immunohistochemistry is fraught with caveats [33]. We developed a predictive model as a clinical diagnostic aid that incorporates Olig2 features, % of cells with strong p53 immunoreactivity, and the probability based on age and ATRX status. The goal of this model is to provide guidance in the event that ATRX immunohistochemistry is either unavailable or equivocal. Our validation set (Figure 6) included 5 false‐positive 1p/19q FISH results, only two of which could have been prevented by adherence to cIMPACT‐NOW guidelines, whereas our improved guidelines handle all cases in the validation set and would prevent all false‐positive 1p/19q in the assays we analyzed.

## CONFLICT OF INTEREST

The authors have no conflicts of interest to declare that are relevant to the content of this article.

## AUTHOR CONTRIBUTIONS

Clinical context and clinical informatics: Diana L. Thomas, José Javier Otero, Lokman Cevik, Mehmet Tahir Aslan, Wesley Wang; Data analysis and data visualization: Catherine Czeisler, José Javier Otero, Lokman Cevik, Mehmet Tahir Aslan, Wesley Wang; Data acquisition: Francisco Jose Garagorry Guerra, Yolanda Cabello‐Izquierdo, Wesley Wang, Jing Zhao, Aline Paixao Becker, Catherine Czeisler, Anne Costa Rendeiro, Lucas Luis Sousa Véras, Maicon Fernando Zanon, Rui Manuel Reis, Marcus de Medeiros Matsushita, Koray Ozduman, M. Necmettin Pamir, Ayca Ersen Danyeli, Thomas Pearce, Michelle Felicella, Jennifer Eschbacher, Naomi Arakaki, Horacio Martinetto, Anil Parwani; Writing—original draft preparation: Diana L. Thomas, José Javier Otero, Lokman Cevik, Marilyn Vazquez Landrove, Wesley Wang; Writing—review and editing: all authors; Mathematical and statistical modeling: Marilyn Vazquez Landrove, Jing Zhao.

## ETHICAL APPROVAL

This study involved an analysis of retrospectively collected individual patient information and digital images in patients with *IDH*‐mutant gliomas. This study was approved by the ethics board of the Ohio State University and all the local ethics boards.

## Supporting information

Fig S1‐S58
**FIGURE S1** Anaplastic astrocytoma, WHO grade 2, IDH−mutated
**FIGURE S2** Anaplastic astrocytoma, WHO grade 2, IDH−wild type
**FIGURE S3** Anaplastic ependymoma, posterior fossa A, WHO grade 3
**FIGURE S4** Anaplastic ependymoma, posterior Fossa B, WHO grade 3
**FIGURE S5** Anaplastic ependymoma, spine, WHO grade 3
**FIGURE S6** Anaplastic ependymoma, supratentorial−RELA, WHO grade 3
**FIGURE S7** Anaplastic ependymoma, supratentorial−YAP, WHO grade 3
**FIGURE S8** Anaplastic ganglioglioma
**FIGURE S9** Anaplastic oligodendroglioma
**FIGURE S10** Anaplastic pleomorphic xanthoastrocytoma
**FIGURE S11** Angiocentric glioma
**FIGURE S12** Astroblastoma
**FIGURE S13** Astrocytoma, WHO grade 2, IDH−mutated
**FIGURE S14** Astrocytoma, WHO grade 2, IDH−wild type
**FIGURE S15** Atypical choroid plexus papilloma, WHO grade 2
**FIGURE S16** Atypical rhabdoid tumour
**FIGURE S17** Central neurocytoma
**FIGURE S18** Cerebellar liponeurocytoma, WHO grade 2
**FIGURE S19** Choroid glioma of the third ventricle
**FIGURE S20** Choroid plexus carcinoma
**FIGURE S21** Choroid plexus papilloma
**FIGURE S22** Desmoplastic infantile astrocytoma, WHO grade 1
**FIGURE S23** Desmoplastic infantile ganglioglioma
**FIGURE S24** Diffuse leptomeningeal glioneuronal tumor
**FIGURE S25** Diffuse midline glioma, WHO grade 4
**FIGURE S26** Diffuse oligodendroglioma, WHO grade 2
**FIGURE S27** Dysembryoplastic neuroepithelial Tumor
**FIGURE S28** Dysplastic cerebellar gangliocytoma, WHO grade 1
**FIGURE S29** Ependymoma, posterior fossa A, WHO grade 2
**FIGURE S30** Ependymoma, posterior fossa B, WHO grade 2
**FIGURE S31** Ependymoma, spine, WHO grade 2
**FIGURE S32** Ependymoma, supratentorial‐YAP, WHO grade 2
**FIGURE S33** Ependymoma, supratentorial, RELA, WHO grade 2
**FIGURE S34** Extraventricular neurocytoma
**FIGURE S35** Gangliocytoma, WHO grade 1
**FIGURE S36** Glioblastoma
**FIGURE S37** Astrocytoma, IDH−mutant, WHO grade 4
**FIGURE S38** Glioblastoma, IDH−wild type, WHO grade 4
**FIGURE S39** Medulloblastoma, non−WNT/non−SHH, WHO grade 4
**FIGURE S40** Medulloblastoma, SHH class, WHO grade 4
**FIGURE S41** Medulloblastoma, WNT group, WHO grade 4
**FIGURE S42** Meningioma, WHO grade 1
**FIGURE S43** Paraganglioma
**FIGURE S44** Pilocytic astrocytoma
**FIGURE S45** Pilomyxoid astrocytoma
**FIGURE S46** Pleomorphic xanthoastrocytoma, WHO grade 2
**FIGURE S47** Rossete‐forming glioneuronl tumour, WHO grade 1
**FIGURE S48** Schwannoma, WHO grade 1
**FIGURE S49** Solitary Fibrous tumour/hemangiopericytoma grade 1
**FIGURE S50** Subependymoma
**FIGURE S51** Subependymal Giant cell astrocytoma, WHO grade 1
**FIGURES S1–51** Summary of each entity in simulation model. (A) Age distributions, (B) Neuroanatomical distributions, (C) Ki67 distributions, (D) Distribution of histological features, (E) Distribution of immunohistochemical features, (F) Distribution of molecular pathology features. Data represented in panels D, E, and F are violin plots of the distribution of the histological, immunohistochemical, and molecular biomarkers. Each biomarker is listed as a binary readout of 0 = negative, 1 = positive. The violin shape provides an indication of the relative proportion showing positive or negative results for this biomarker. In cases where the literature does not support a distribution, (for instance, necrosis in astrocytoma, a notch is placed at the level of the 0)
**FIGURE S52** Distribution of cases in the global IDH mutant (Grade 2 and 3) dataset. World map shows the distribution of the cases in the global dataset. TCGA study (purple circles) has cases from different areas of the world such as Italy, Germany, Australia, but mostly the United States. This overall distribution of global dataset shows a good variety of ethnicity and genetic background to represent a real‐world data
**FIGURE S53** Deconvolution with Otsu’s thresholding and Watershed. The images were splitted into R, G and B channels. Then R (red), G (green) and B (blue) channels were converted to H (hematoxylin), D (DAB) and X channels. D channel was selected for brown color in OLIG2 staining. After application of median filtering, Otsu's thresholding was applied to the D channel. The holes in the nuclei were filled out and then the nuclei on the border were eliminated for a better representation of nuclei. As a last step, watershed function was applied for overlapping or contacting nuclei
**FIGURE S54** K‐means clustering segmentation. The images were converted to a data frame using R, G and B pixel intensity values. All pixels were clustered into 3 clusters using k‐means clustering method. Each pixel was labeled as cluster 1, cluster 2, or cluster 3. The cluster that contain specifically the nuclei was chosen as foreground and the other 2 clusters were accepted as background
**FIGURE S55** Cut‐Cluster‐Classify (CCC) segmentation method. (A) All patches in the image are viewed as high dimensional points and a threshold is applied to the sample density (Cut) to get clusters. After clustering (Cluster), the patches below the threshold were classified based on the clusters (Classify). (B) Then all pixels were voted for clusters based on cluster result on overlapping patches. The cluster that contains nuclei was accepted as foreground and the other one was accepted as background. 5x5 patches were used for the segmentation
**FIGURE S56** Relationships of R channel pixel intensity values with G and B pixel intensity values for color of noise. Mean pixel intensity values of R channel are correlated to mean pixel intensity values of G and B channels. To better represent these spectrums, we used support vector machine (SVM) modeling to predict G and B values from R. The colors of noise are purple for H&E staining and brown for OLIG2 staining
**FIGURE S57** Image noise assay workflow and comparison of supervised and unsupervised image segmentation methods for H&E and OLIG2 immunostaining. (A) An image noise assay was created for the fidelity evaluation of image segmentation methods to determine the best one for our purpose. We extracted the purple color for H&E staining and brown color for OLIG2 staining. A support vector model (SVM) was made to generate a noise similar to colors of the image. After generation of noise for a full image, the pixels were randomly sampled with certain percentages to implement the noise to the images. This workflow provided us to compare the performance of segmentation methods with different amount of noise. (B and C) We plotted segmentation performance by accuracy and intersection‐over‐union (IoU). Additionally, we added the U‐Net trained with random noise generation as “U‐Net with noise” to the comparison. In U‐Net with noise, we trained a new model every time when we added noise to the image. (B) In H&E staining, U‐Net models show highest accuracies independent from the noise percentage. On the other hand, U‐Net method shows low IoU, when U‐Net with noise has a better IoU values. Overall deconvolution method has the better performance for accuracy and IoU. (C) In OLIG2 staining, similar to the H&E, deconvolution and U‐Net with noise methods have the highest performance regarding accuracy and IoU. Despite deconvolution method has a sharp decrease after 25% noise, U‐Net with noise maintained high IoU even after 25% noise
**FIGURE S58** (A) Gini importance of RF model for the probability of 1p19q codeletion using ATRX and age. (B) Gini importance of final RF model used for the 1p19q codeletion in validation data set. (C) Simulation of the model with different p53 percentages showing 1p19q codeletion probability in different scenarios

Table S1
**TABLE S1** Impact of scrambling features on accuracy of random forest model

Supplementary Material
**TABLE S2** Validation set data

Supplementary Material

## Data Availability

The data that support the findings of this study are openly available in “figshare” at http://doi.org/10.6084/m9.figshare.16493964.
