# Supplementary material for: Information theory approaches to improve glioma diagnostic workflows in surgical neuropathology
Source: Brain Pathol. 2022 Jan 10;32(5):e13050. doi: 10.1111/bpa.13050 (PMC9425010; doi:10.1111/bpa.13050)
Supplement: Supplementary file 1 — Fig S1‐S58 FIGURE S1 Anaplastic astrocytoma, WHO grade 2, IDH−mutated FIGURE S2 Anaplastic astrocytoma, WHO grade 2, IDH−wild type FIGURE S3 Anaplastic ependymoma, posterior fossa A, WHO grade 3 FIGURE S4 Anaplastic ependymoma, posterior Fossa B, WHO grade 3 FIGURE S5 Anaplastic ependymoma, spine, WHO grade 3 FIGURE S6 Anaplastic ependymoma, supratentorial−RELA, WHO grade 3 FIGURE S7 Anaplastic ependymoma, supratentorial−YAP, WHO grade 3 FIGURE S8 Anaplastic ganglioglioma FIGURE S9 Anaplastic oligodendroglioma FIGURE S10 Anaplastic pleomorphic xanthoastrocytoma FIGURE S11 Angiocentric glioma FIGURE S12 Astroblastoma FIGURE S13 Astrocytoma, WHO grade 2, IDH−mutated FIGURE S14 Astrocytoma, WHO grade 2, IDH−wild type FIGURE S15 Atypical choroid plexus papilloma, WHO grade 2 FIGURE S16 Atypical rhabdoid tumour FIGURE S17 Central neurocytoma FIGURE S18 Cerebellar liponeurocytoma, WHO grade 2 FIGURE S19 Choroid glioma of the third ventricle FIGURE S20 Choroid plexus carcinoma FIGURE S21 Choroid plexus papilloma FIGURE S22 Desmoplastic infantile astrocytoma, WHO grade 1 FIGURE S23 Desmoplastic infantile ganglioglioma FIGURE S24 Diffuse leptomeningeal glioneuronal tumor FIGURE S25 Diffuse midline glioma, WHO grade 4 FIGURE S26 Diffuse oligodendroglioma, WHO grade 2 FIGURE S27 Dysembryoplastic neuroepithelial Tumor FIGURE S28 Dysplastic cerebellar gangliocytoma, WHO grade 1 FIGURE S29 Ependymoma, posterior fossa A, WHO grade 2 FIGURE S30 Ependymoma, posterior fossa B, WHO grade 2 FIGURE S31 Ependymoma, spine, WHO grade 2 FIGURE S32 Ependymoma, supratentorial‐YAP, WHO grade 2 FIGURE S33 Ependymoma, supratentorial, RELA, WHO grade 2 FIGURE S34 Extraventricular neurocytoma FIGURE S35 Gangliocytoma, WHO grade 1 FIGURE S36 Glioblastoma FIGURE S37 Astrocytoma, IDH−mutant, WHO grade 4 FIGURE S38 Glioblastoma, IDH−wild type, WHO grade 4 FIGURE S39 Medulloblastoma, non−WNT/non−SHH, WHO grade 4 FIGURE S40 Medulloblastoma, SHH class, WHO grade 4 FIGURE S41 Medulloblastoma, WNT group, W [file BPA-32-e13050-s003.pdf]

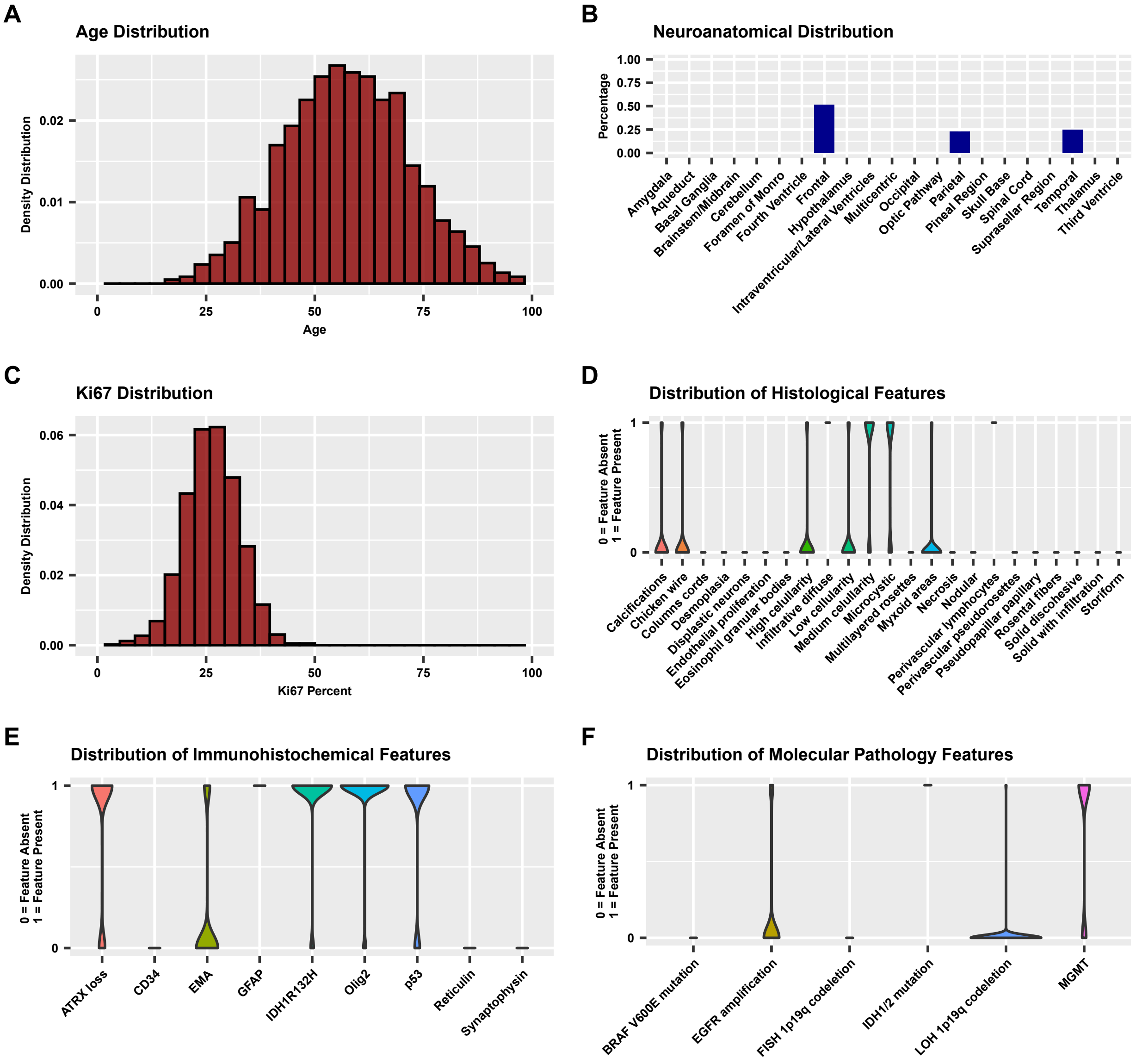

Figure S1. Anaplastic Astrocytoma, WHO grade 3, IDH−mutated

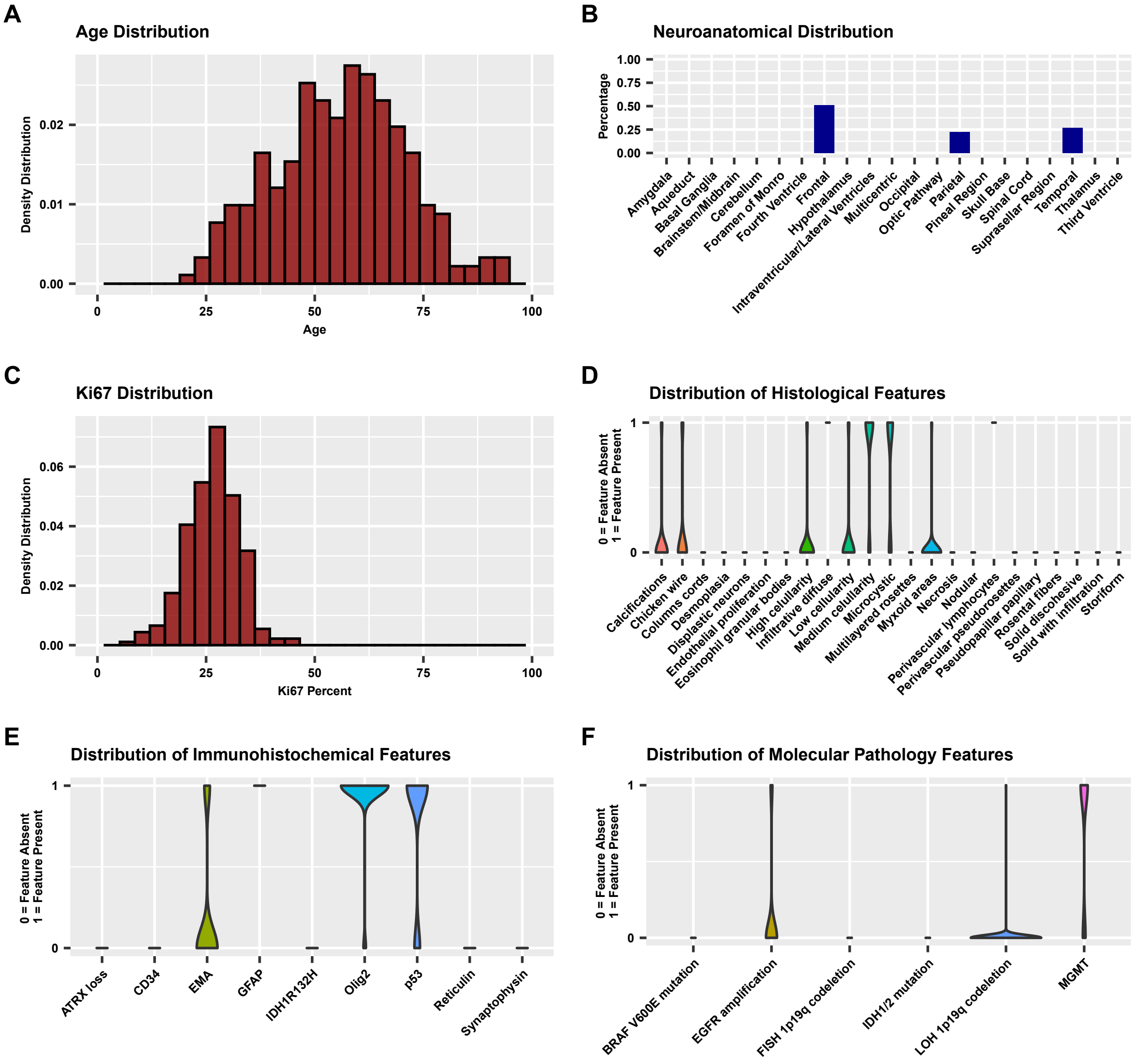

Figure S2. Anaplastic Astrocytoma, WHO grade 3, IDH−wild type

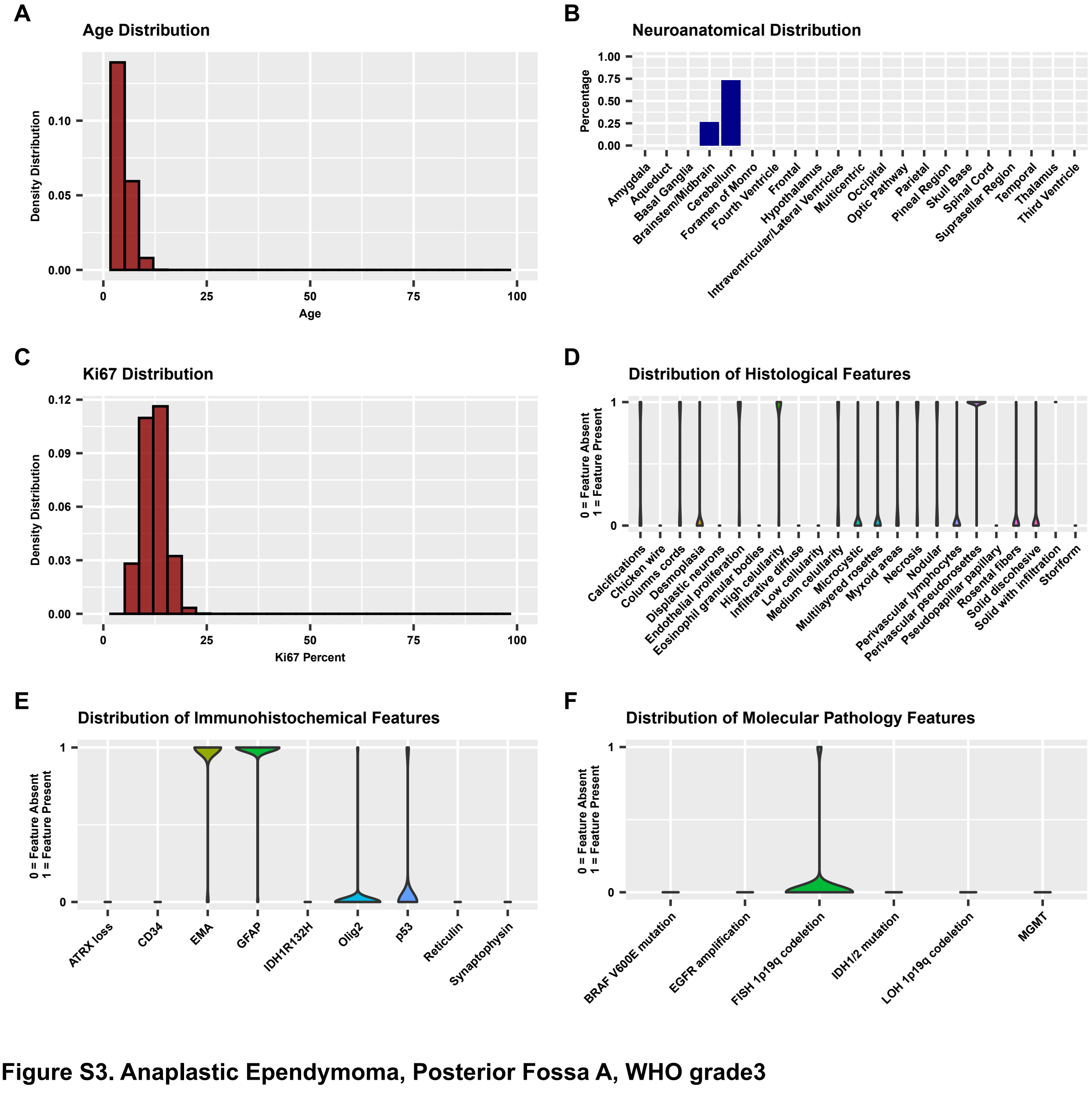

Figure S3. Anaplastic Ependymoma, Posterior Fossa A, WHO grade3

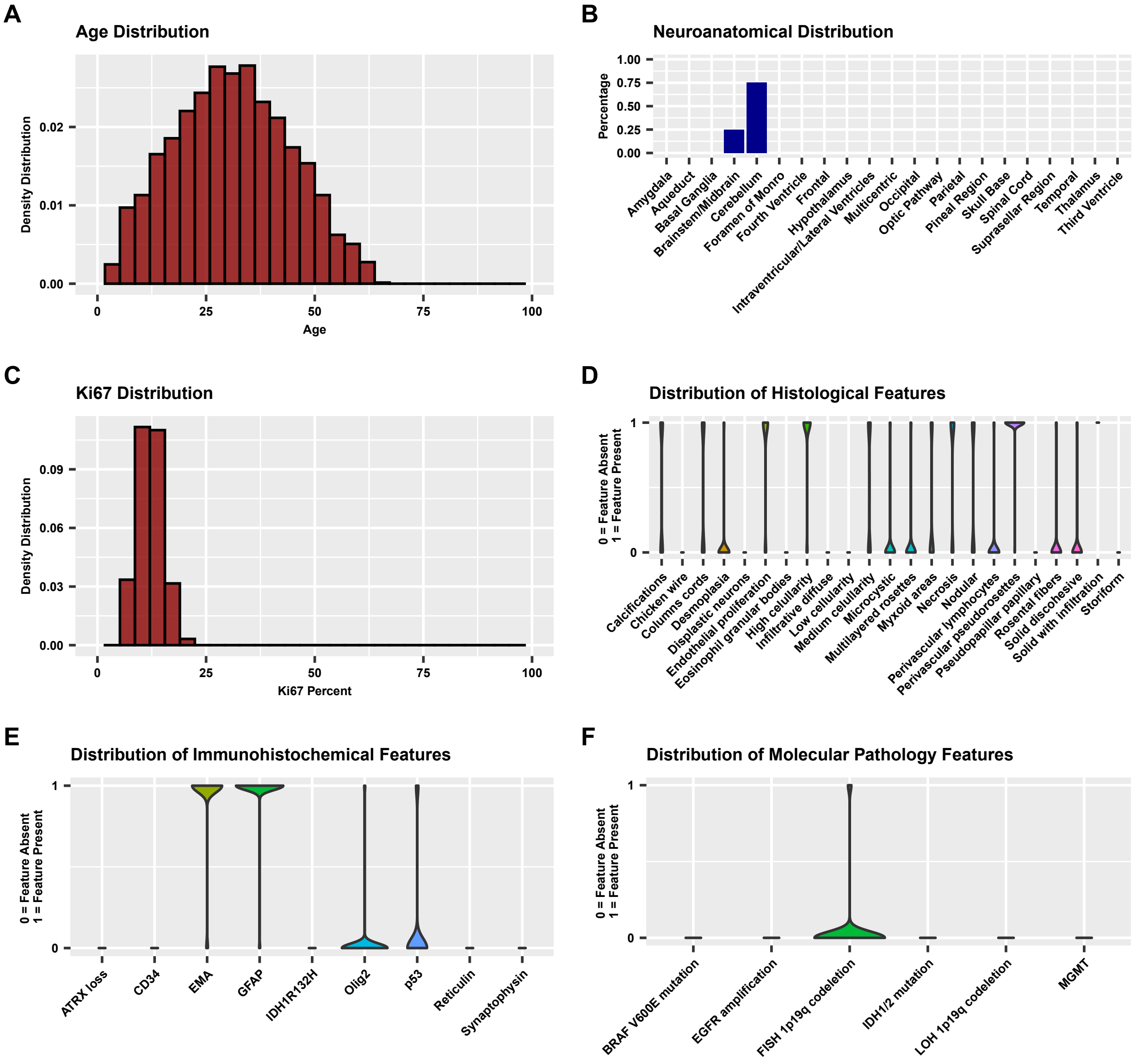

Figure S4. Anaplastic Ependymoma, Posterior Fossa B, WHO grade 3

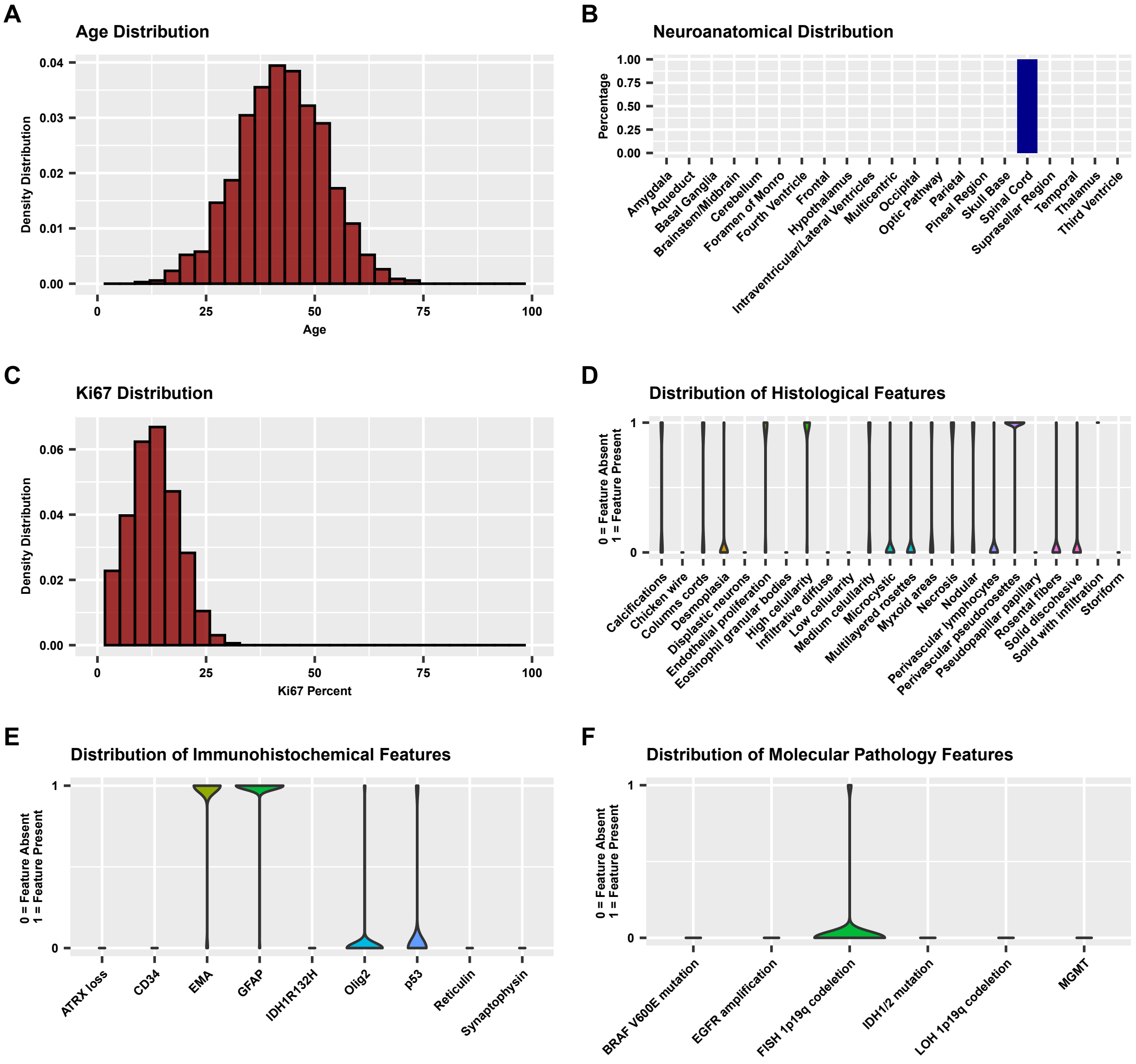

Figure S5. Anaplastic Ependymoma, Spine, WHO grade 3

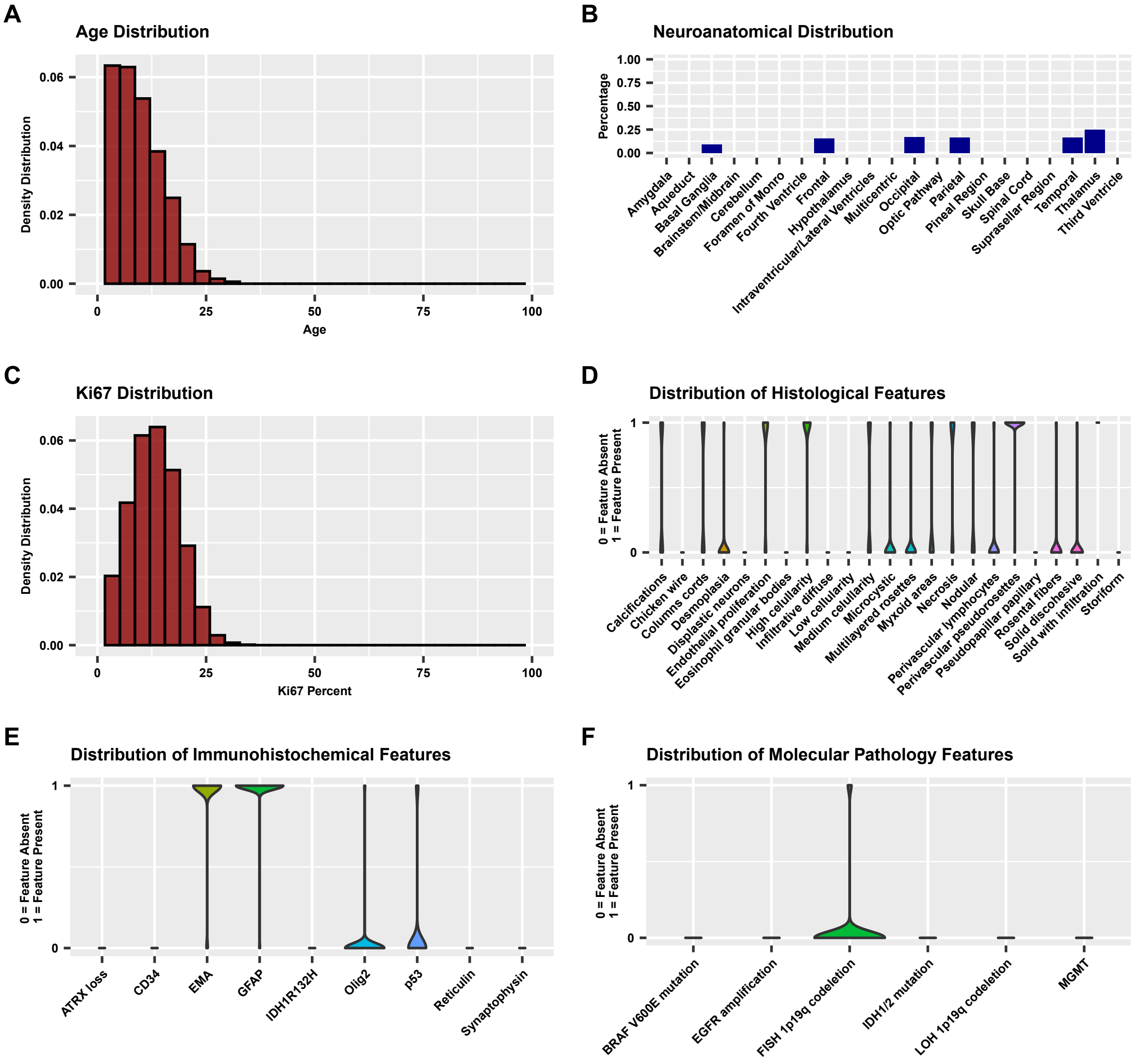

Figure S6. Anaplastic Ependymoma, Supratentorial−RELA, WHO grade 3

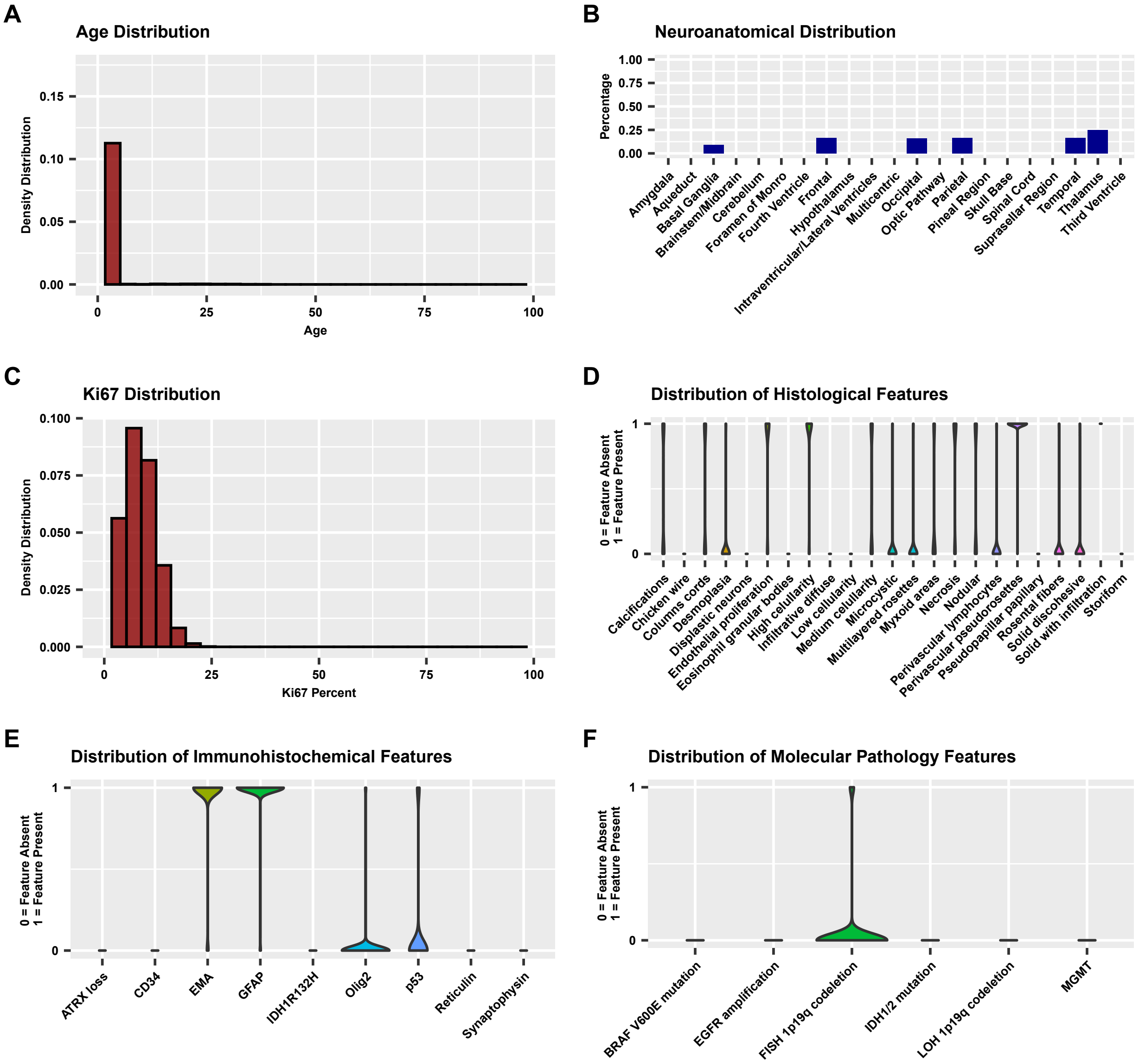

Figure S7. Anaplastic Ependymoma, Supratentorial−YAP, WHO grade 3

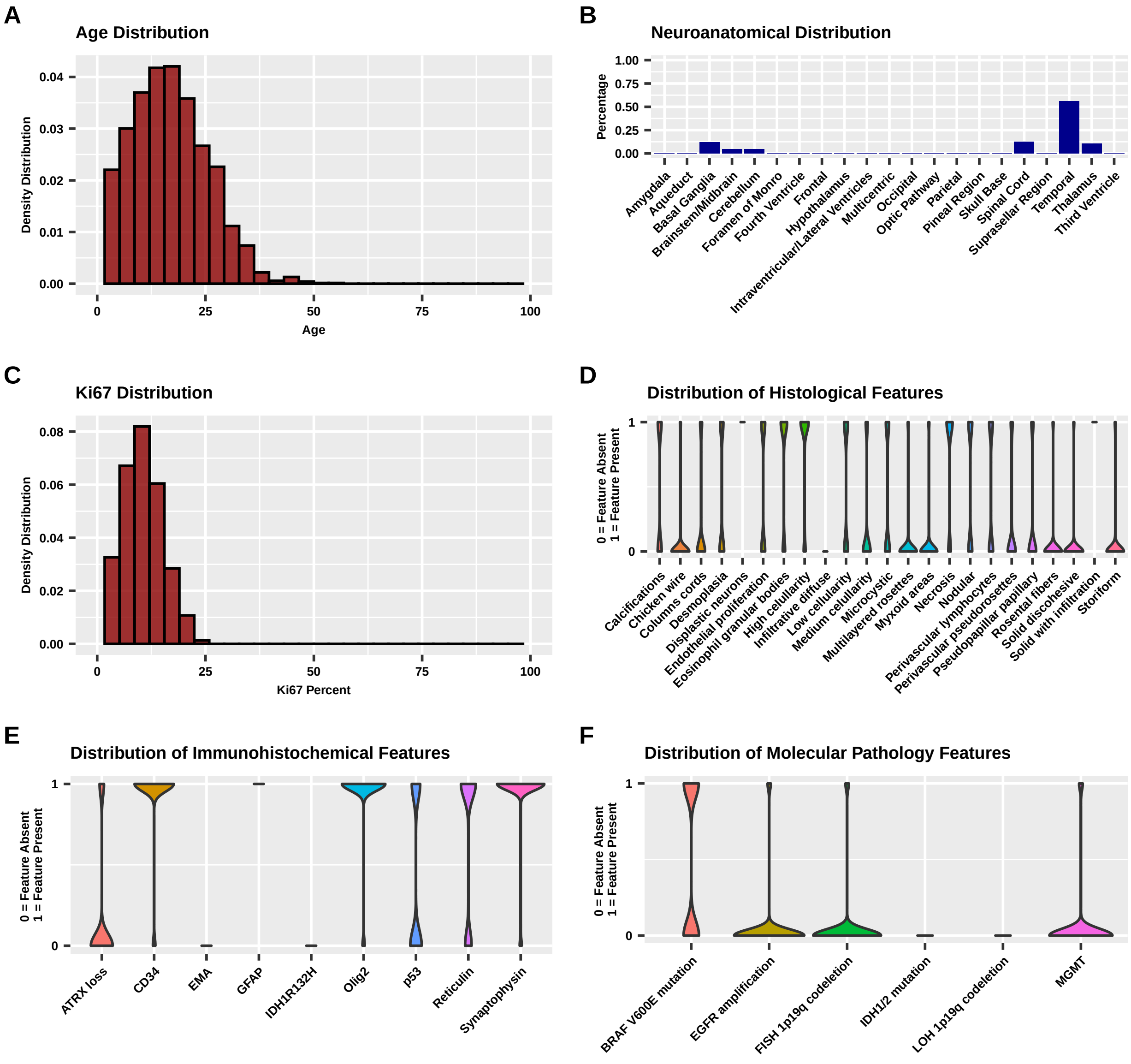

Figure S8. Anaplastic Ganglioglioma

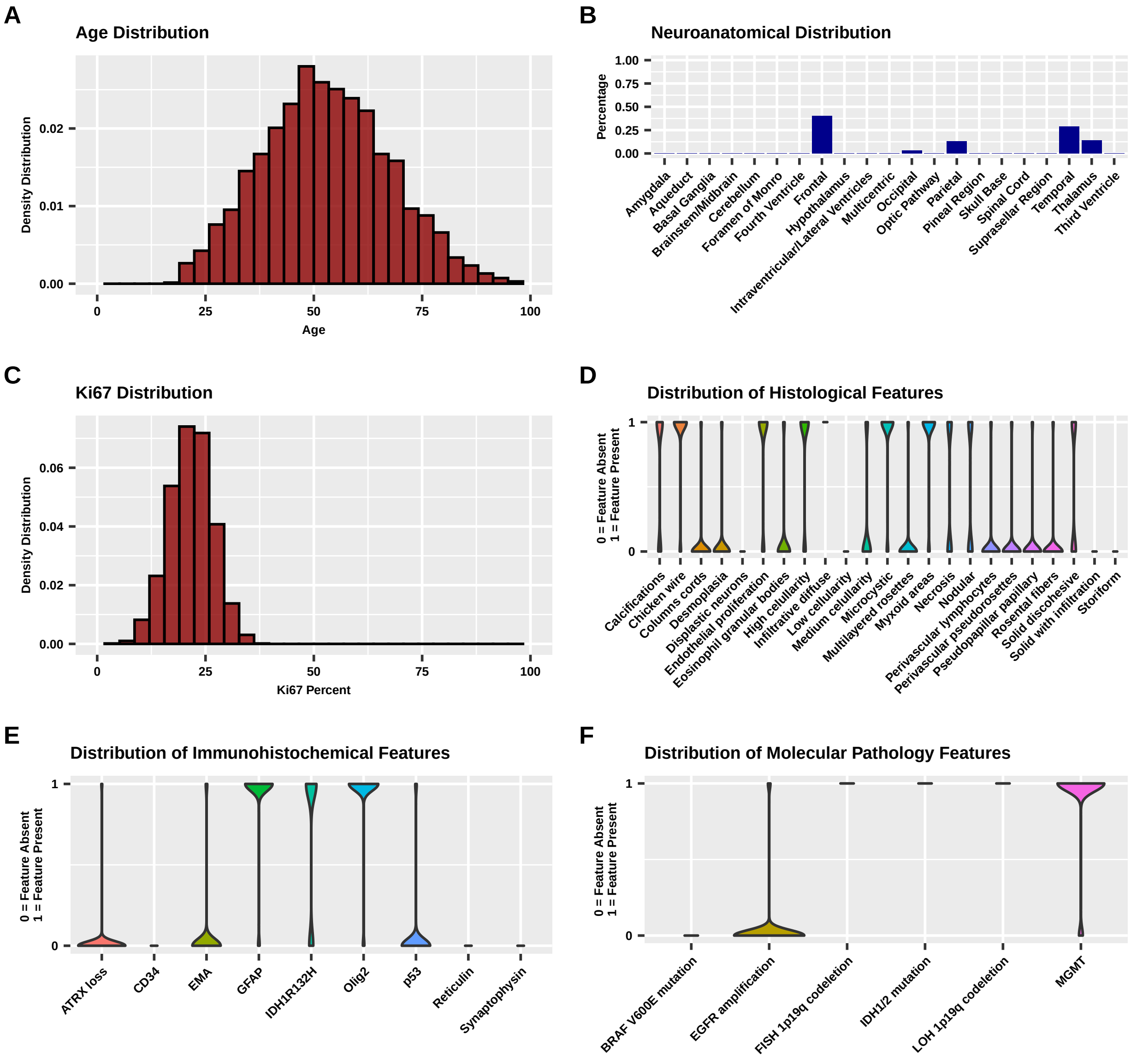

Figure S9. Anaplastic Oligodendroglioma

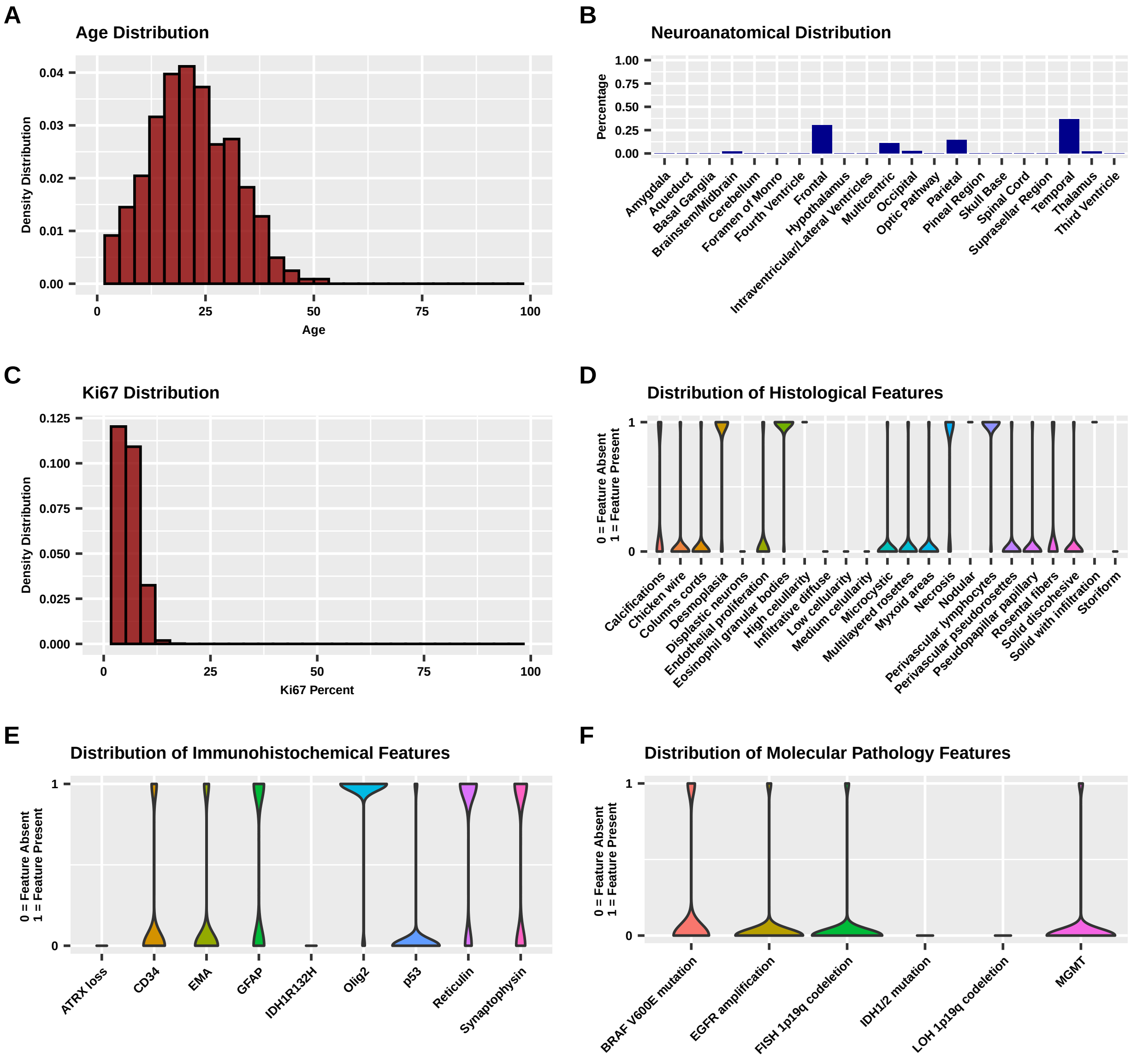

Figure S10. Anaplastic Pleomorphic Xanthoastrocytoma

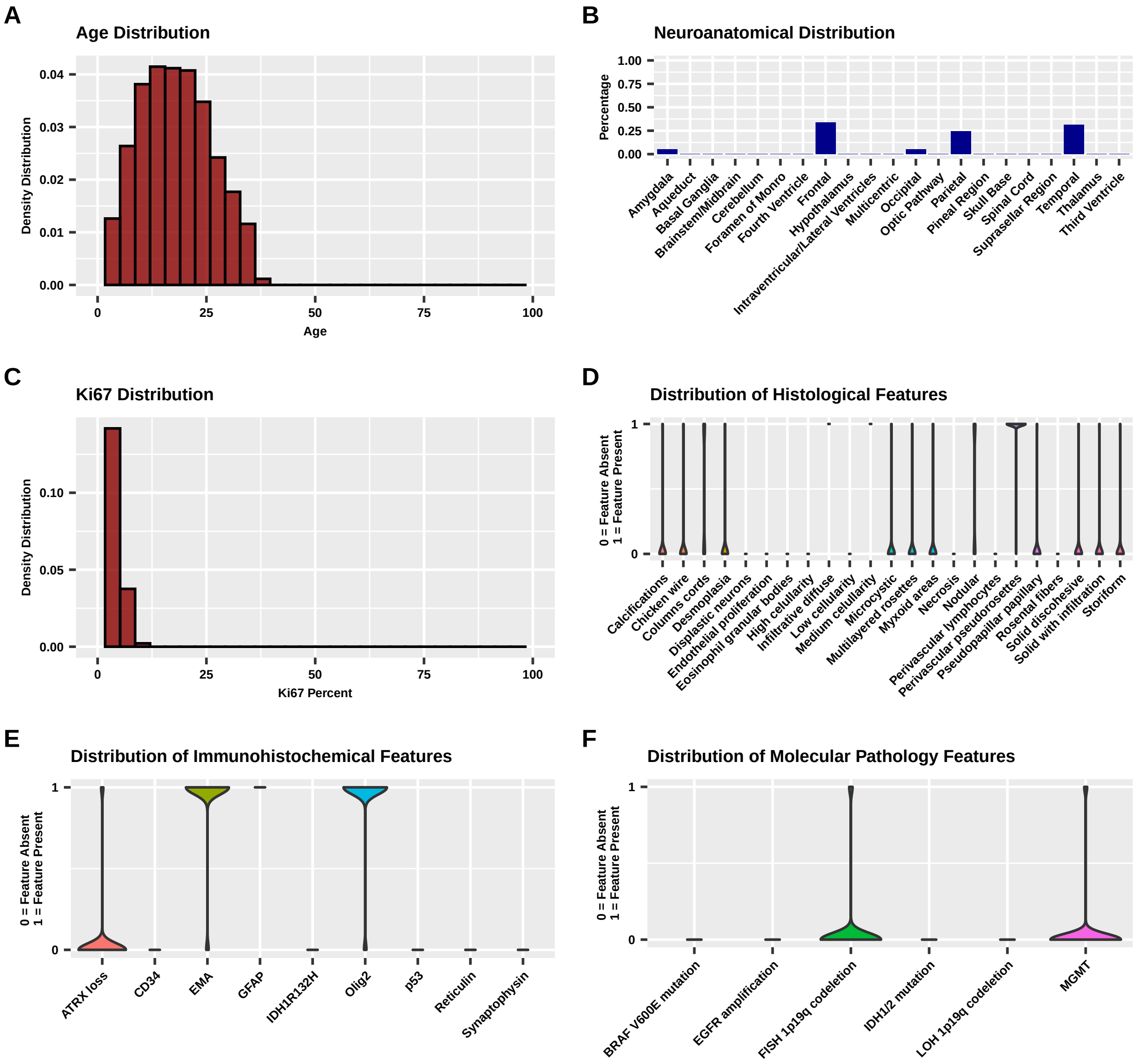

Figure S11. Angiocentric Glioma

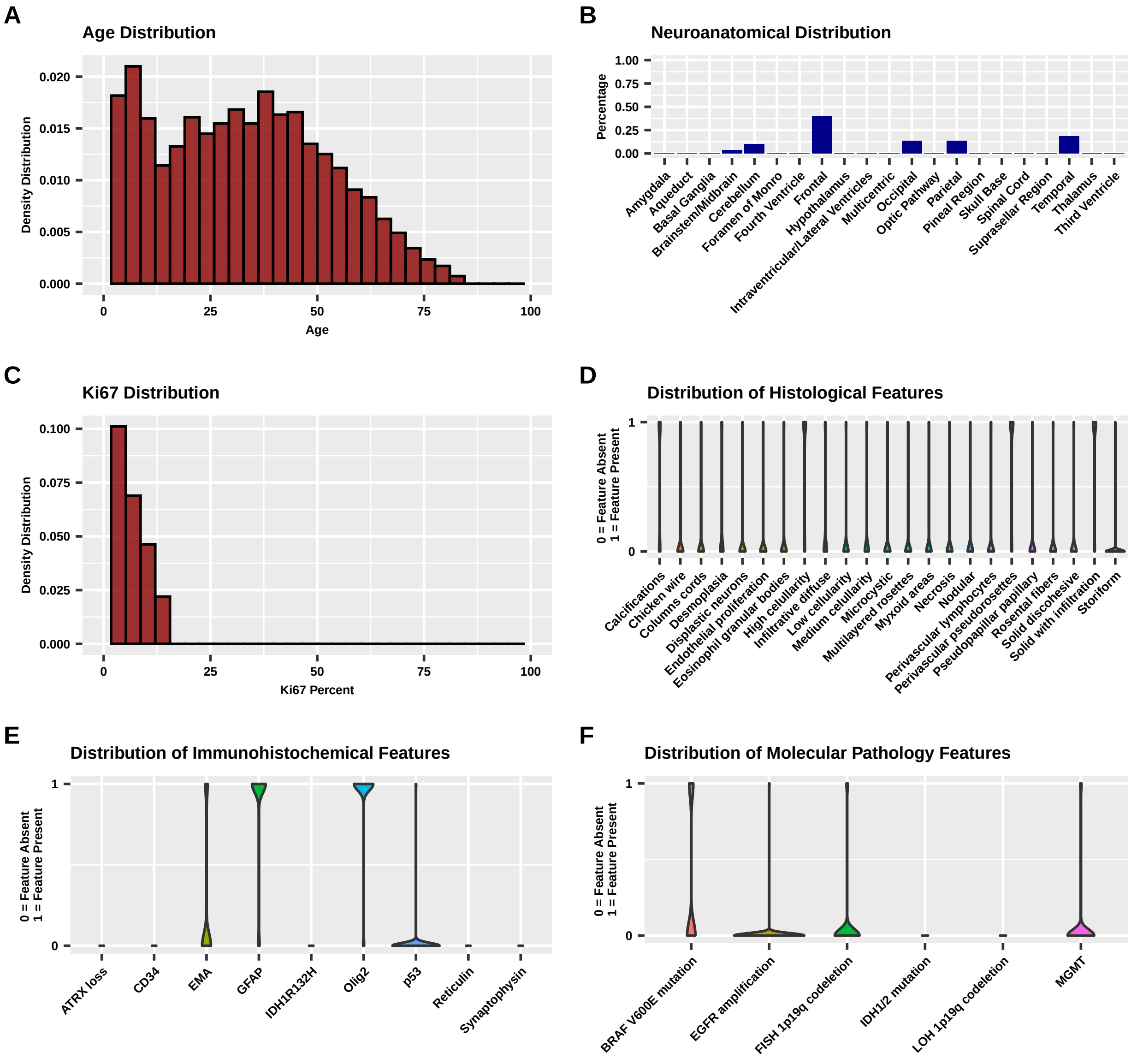

Figure S12. Astroblastoma

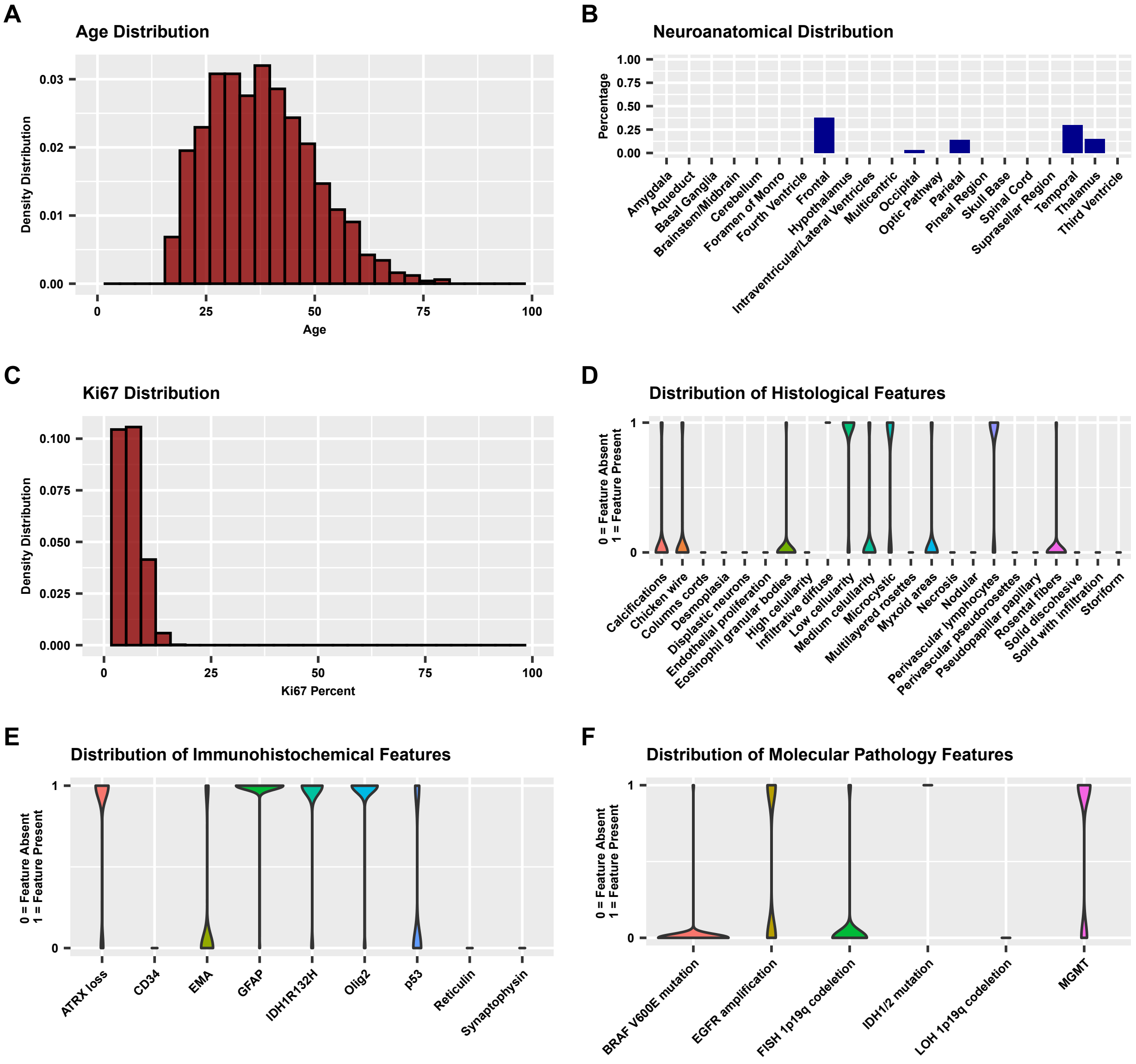

Figure S13. Astrocytoma, WHO grade 2, IDH−Mutated

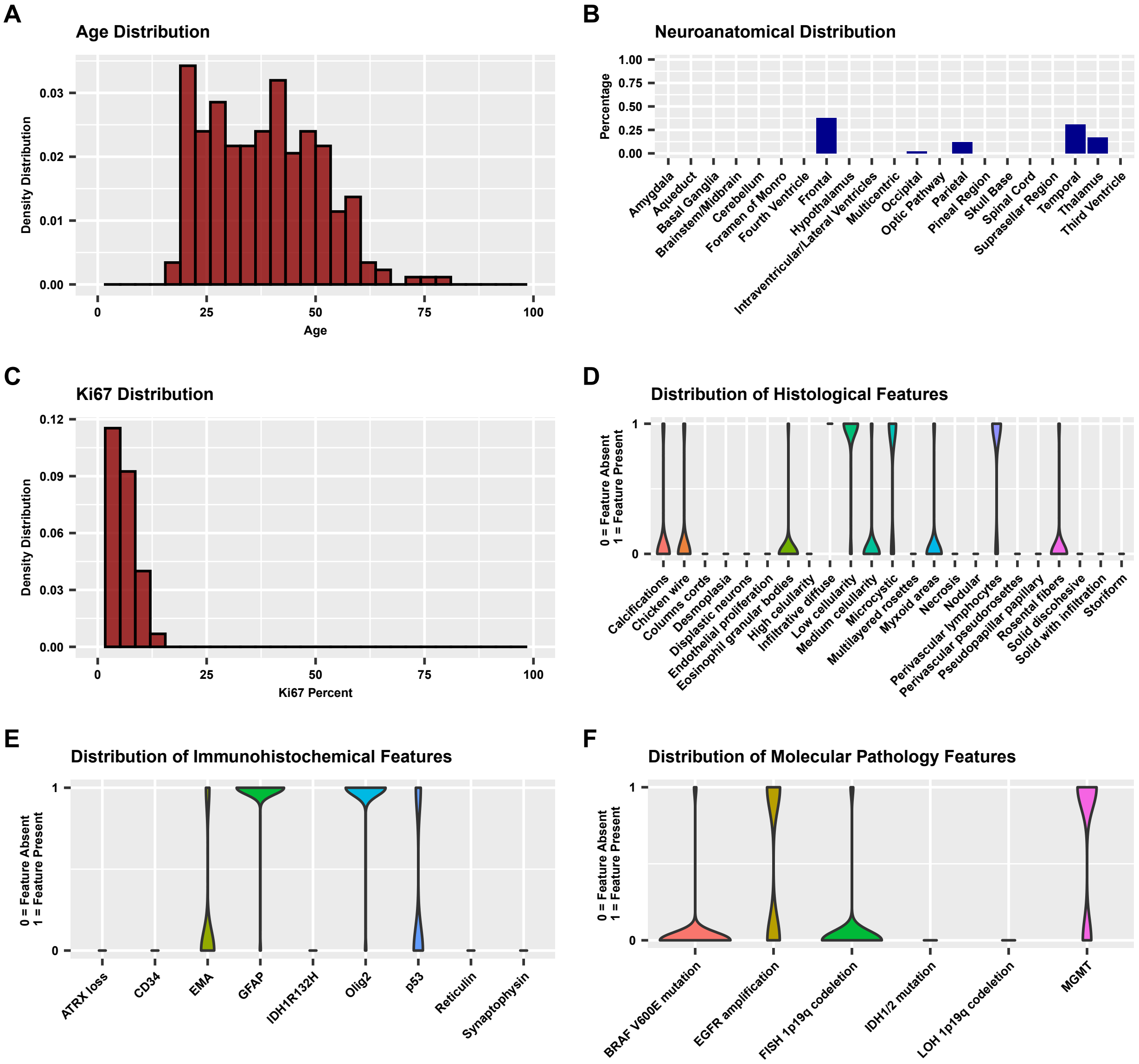

Figure S14. Astrocytoma, WHO grade 2, IDH−wild type

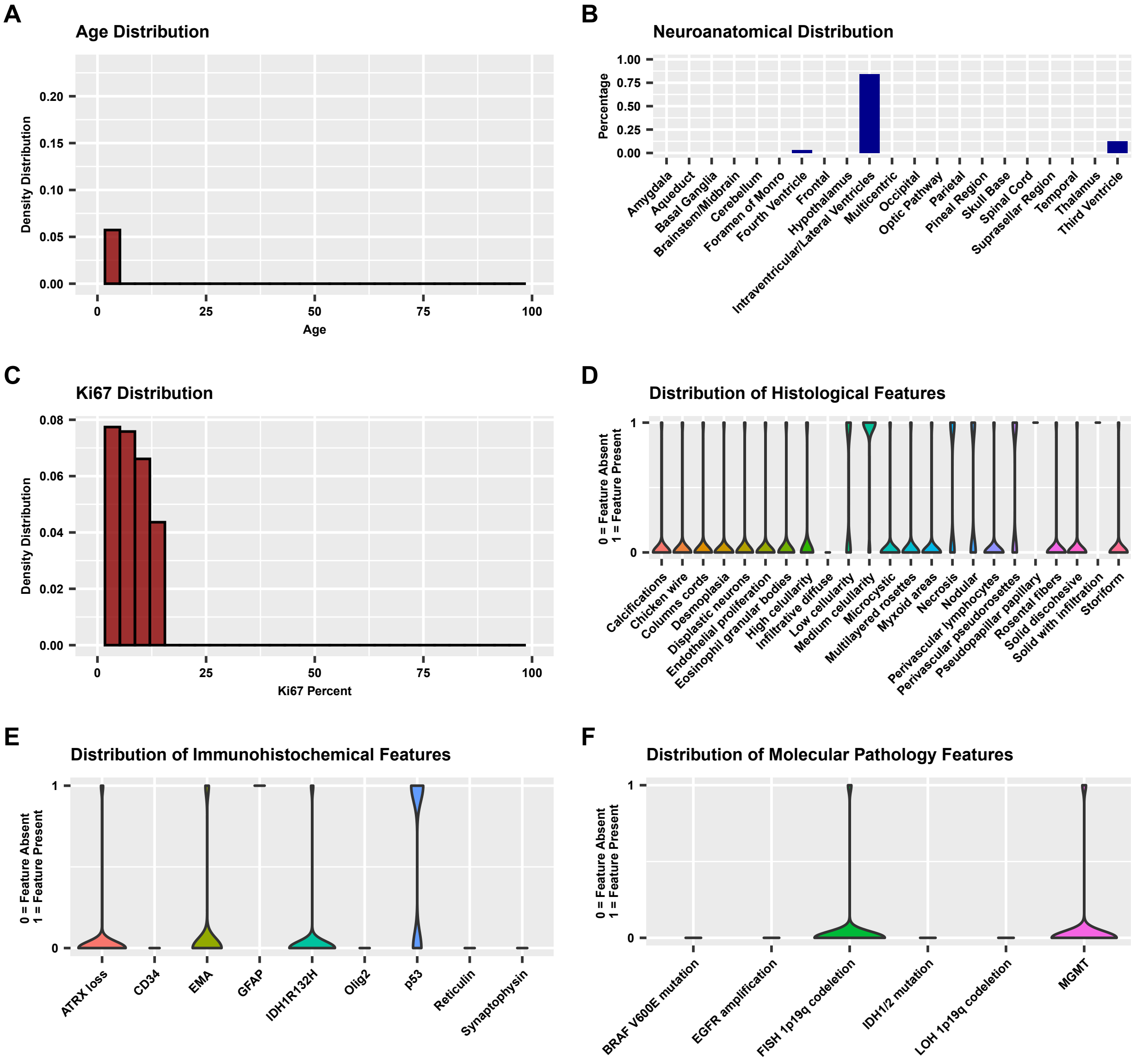

Figure S15. Atypical Choroid Plexus Papilloma, WHO grade 2

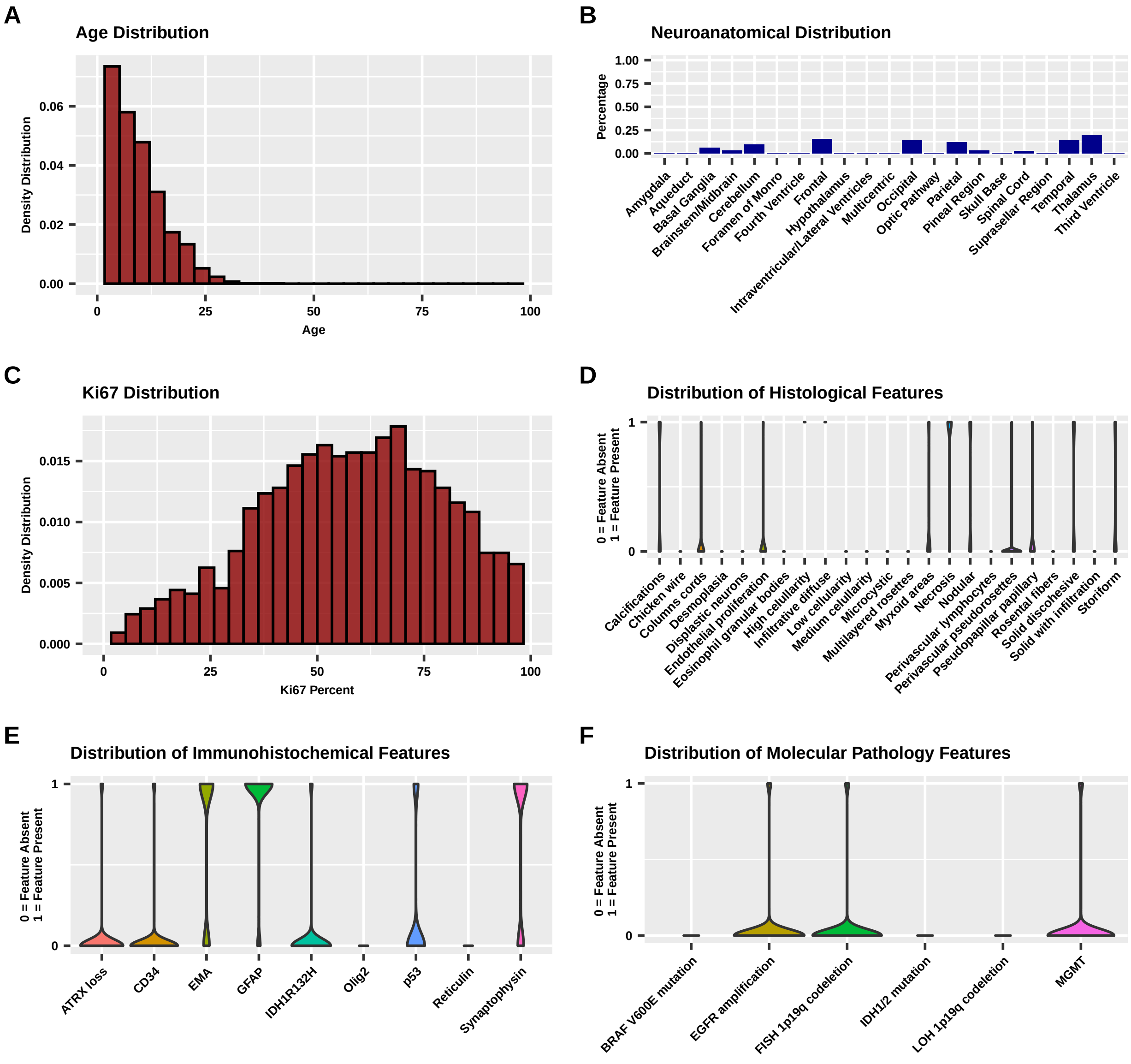

Figure S16. Atypical Rhabdoid Tumour

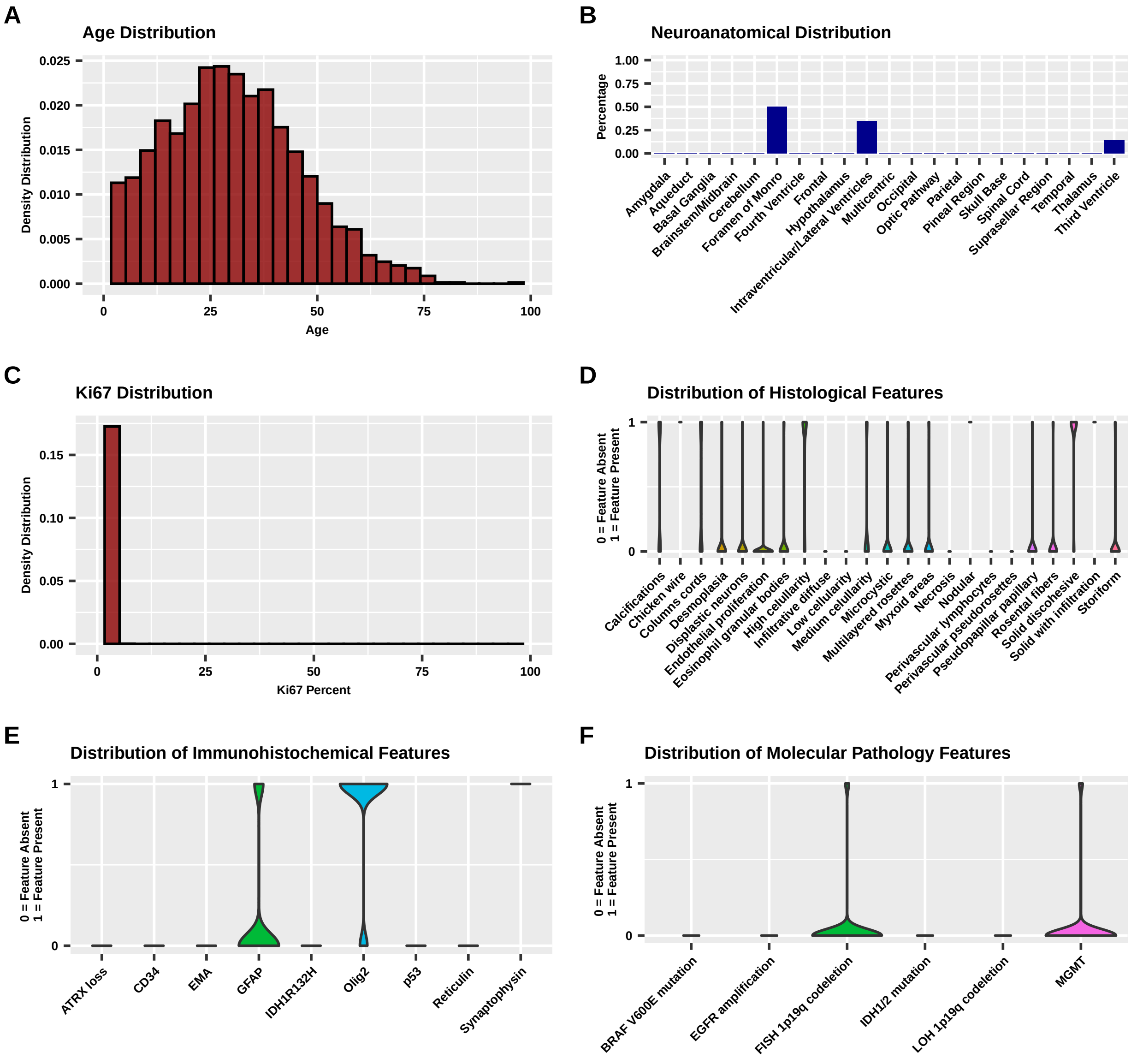

Figure S17. Central Neurocytoma

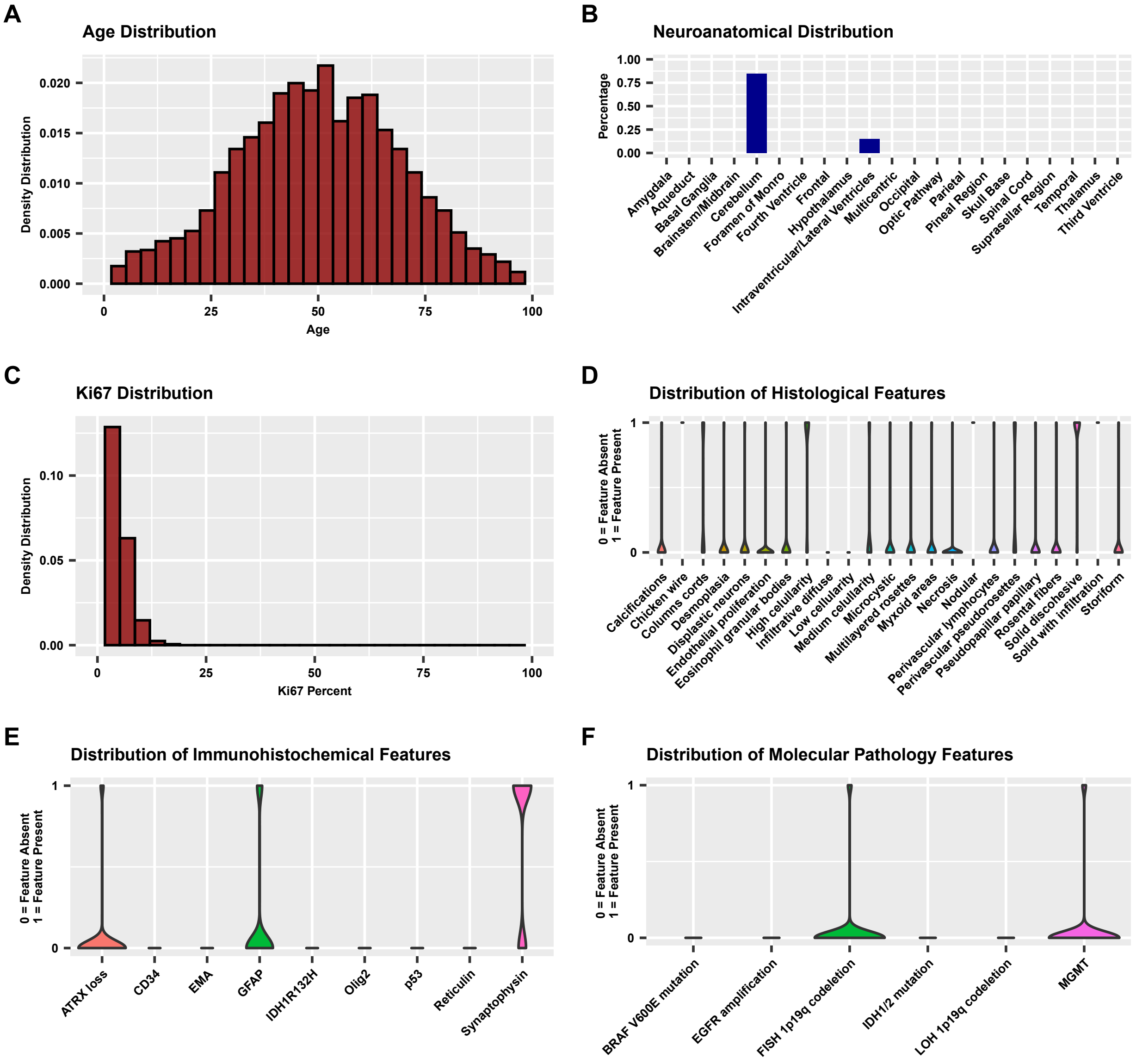

Figure S18. Cerebellar Liponeurocytoma, WHO grade 2

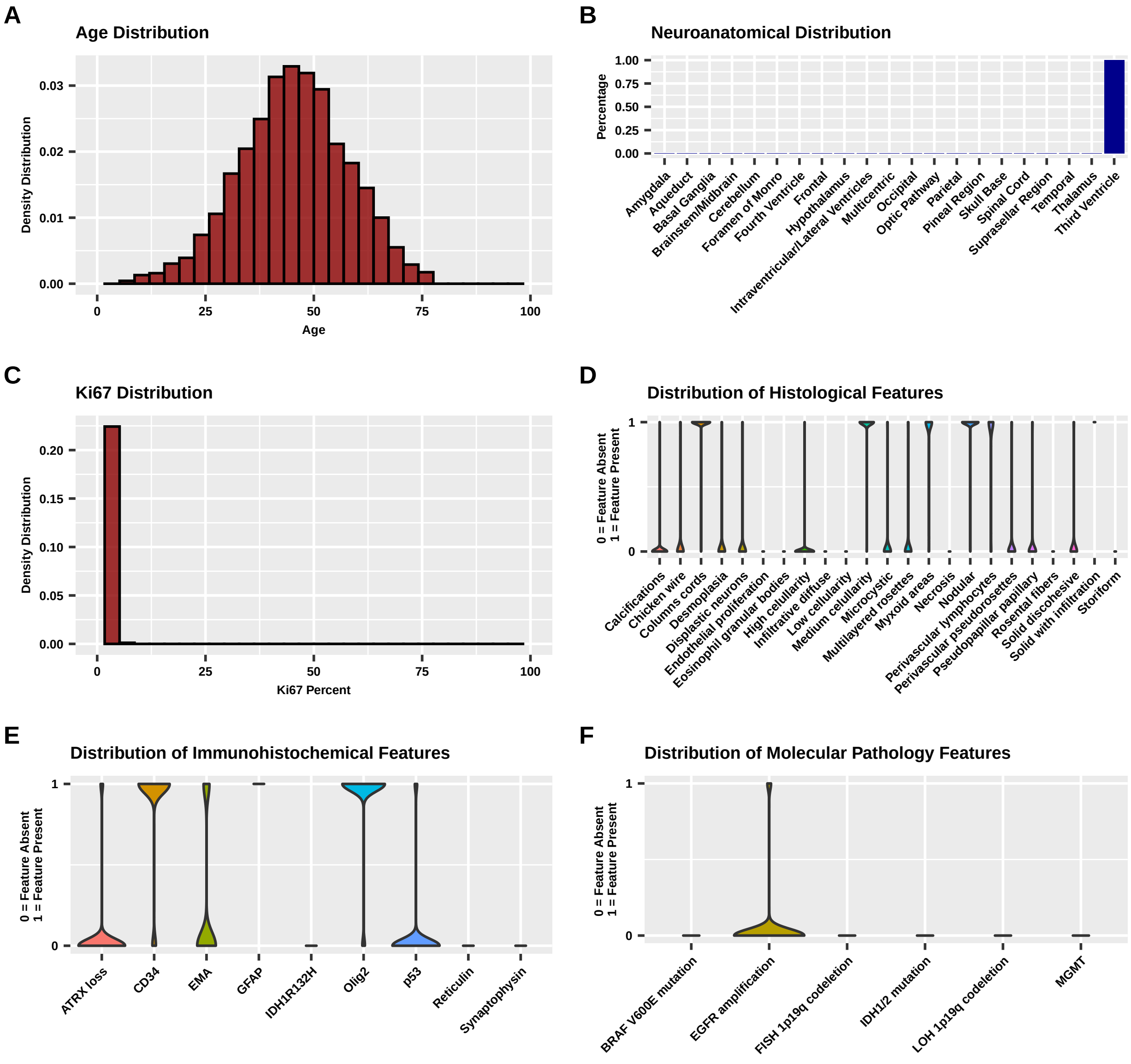

Figure S19. Choroid Glioma Of The Third Ventricle

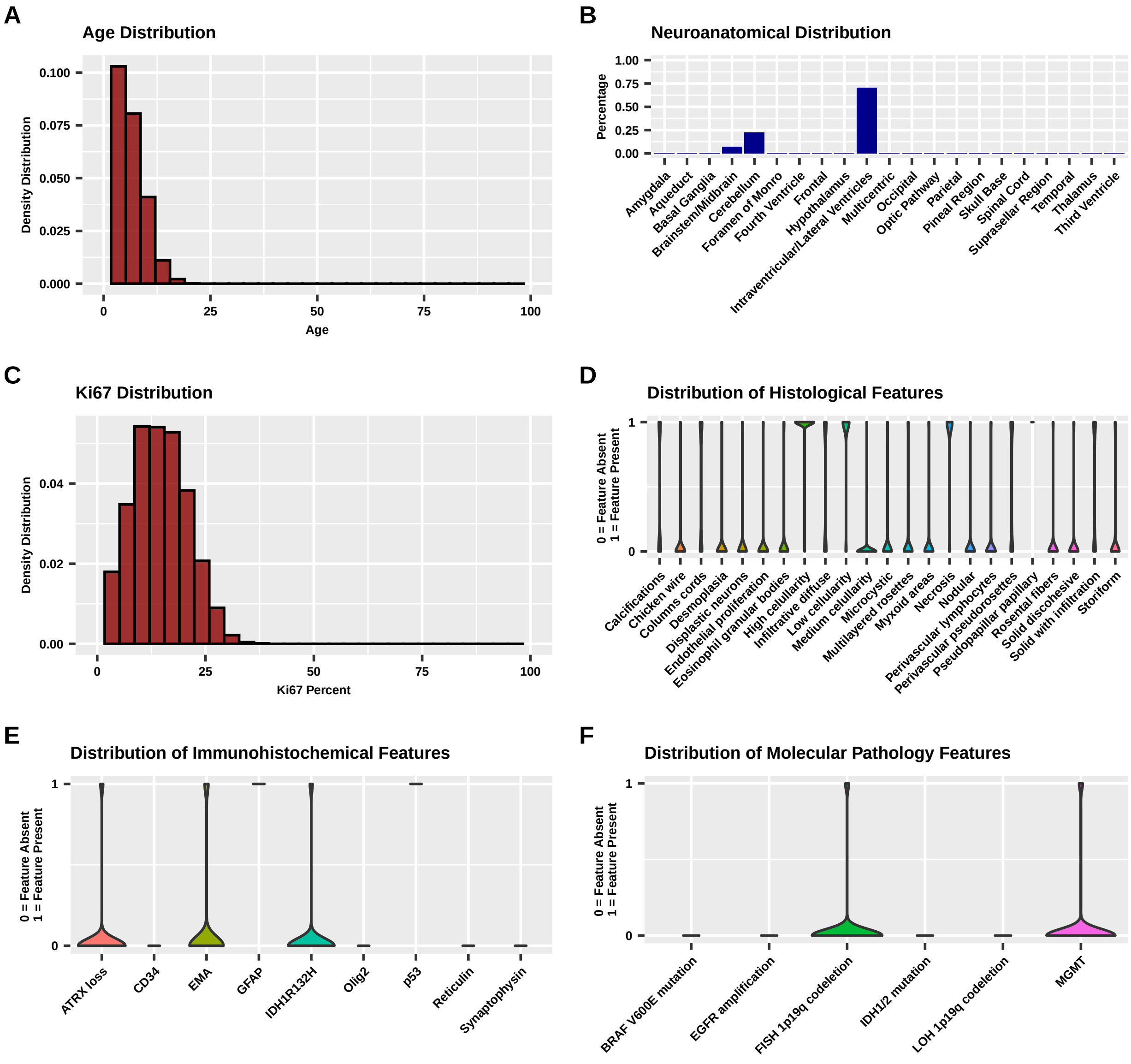

Figure S20. Choroid Plexus Carcinoma

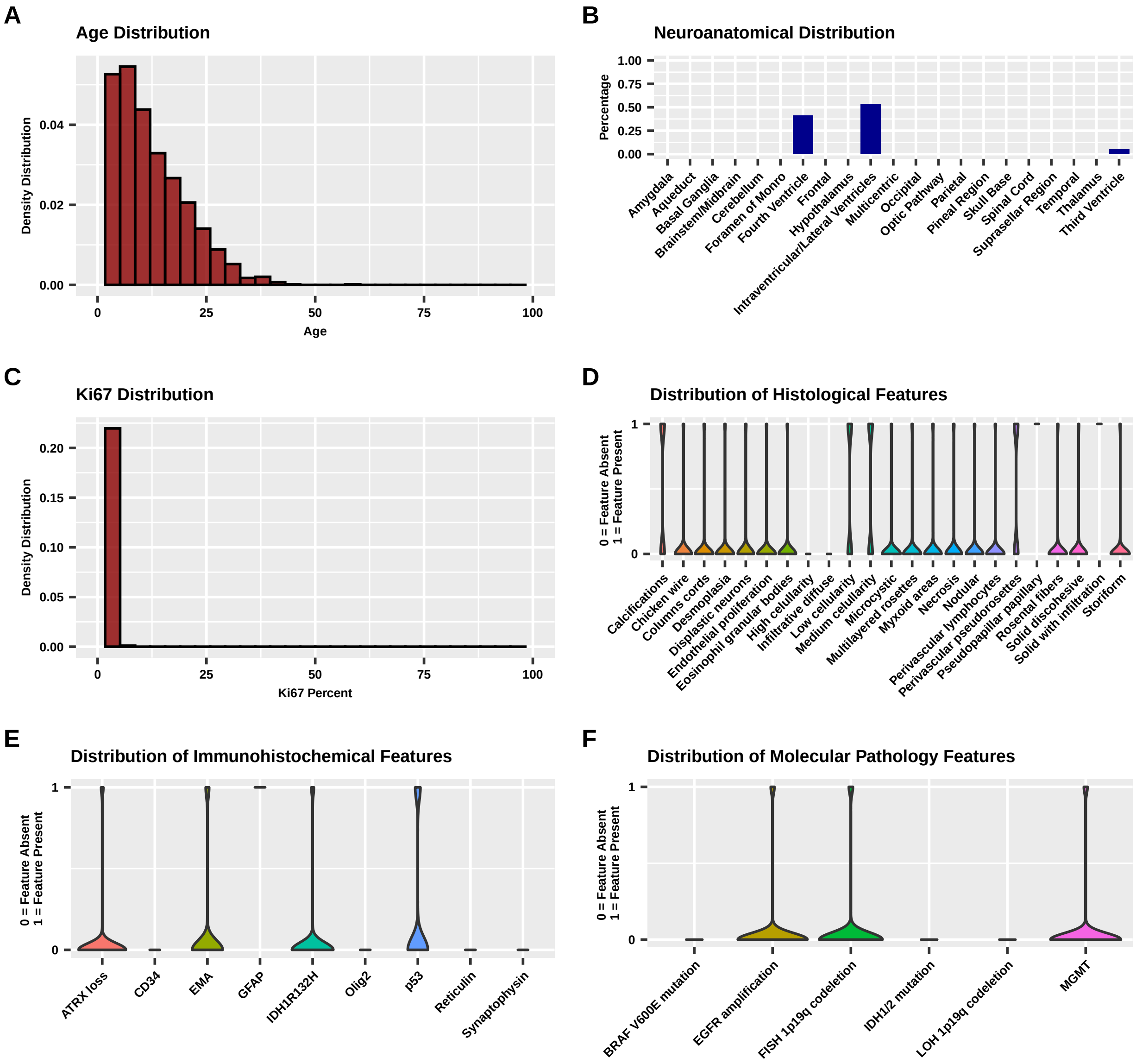

Figure S21. Choroid Plexus Papilloma

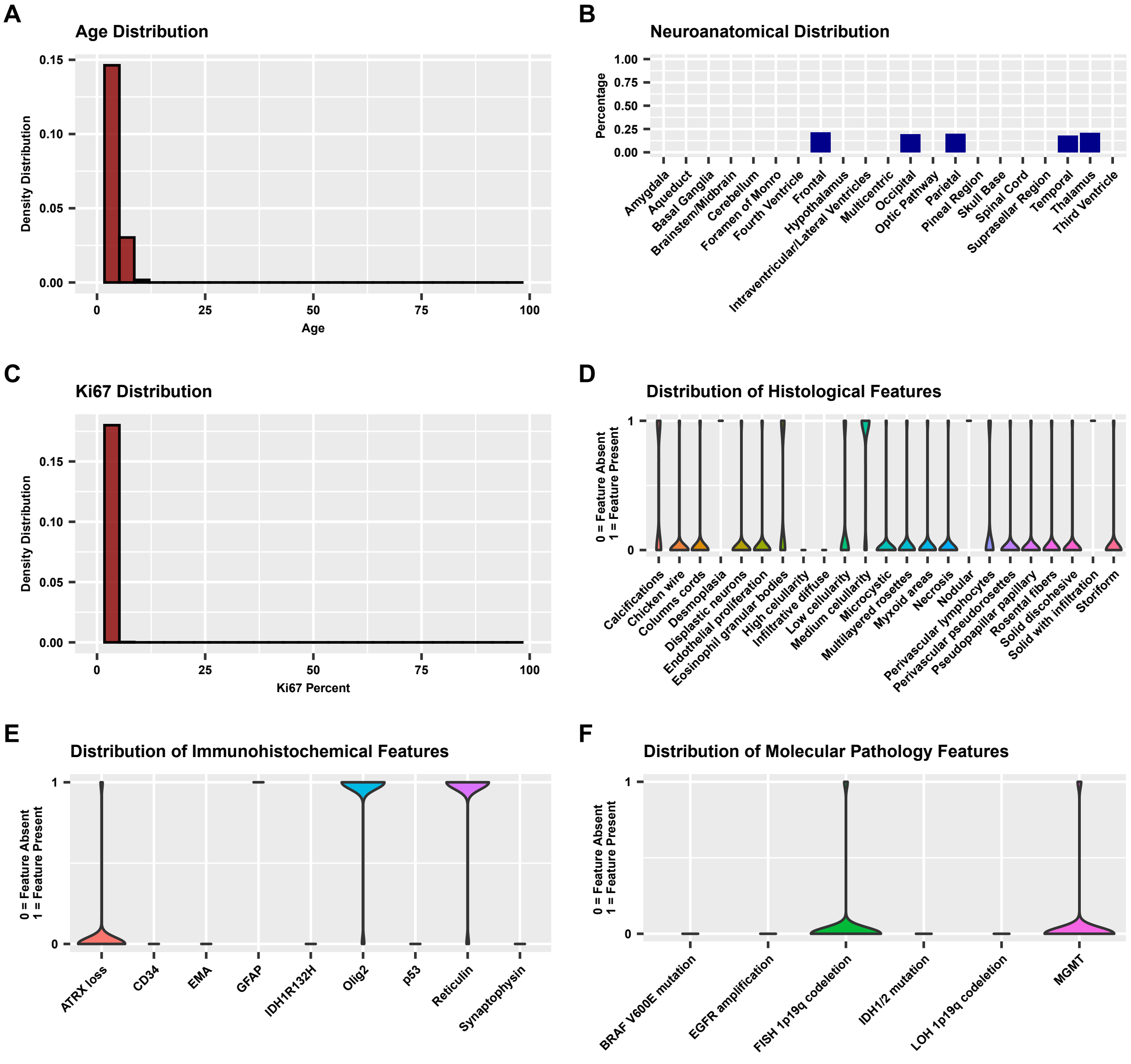

Figure S22. Desmoplastic Infantile Astrocytoma, WHO grade 1

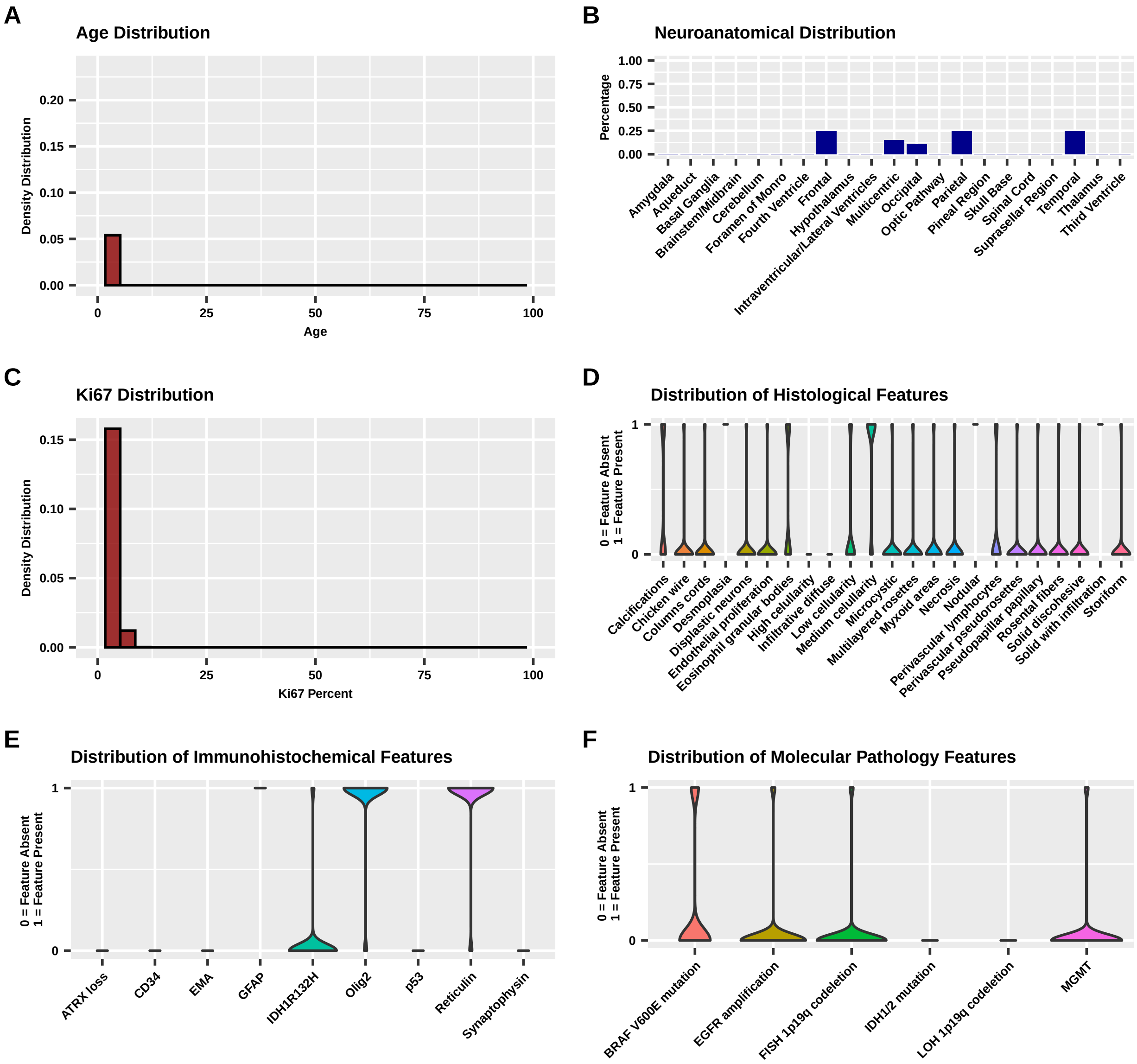

Figure S23. Desmoplastic Infantile Ganglioglioma

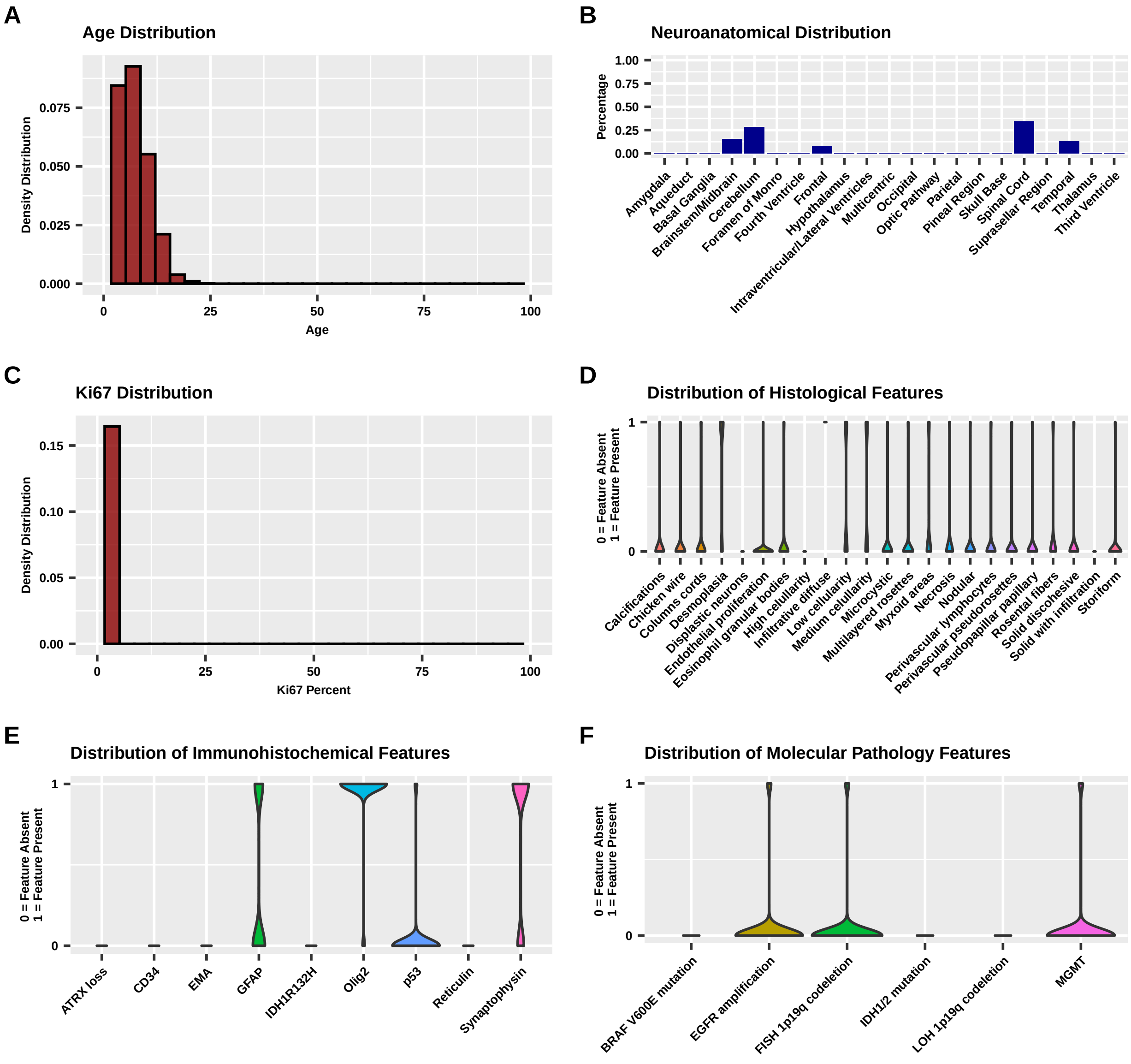

Figure S24. Diffuse Leptomeningeal Glioneuronal Tumour

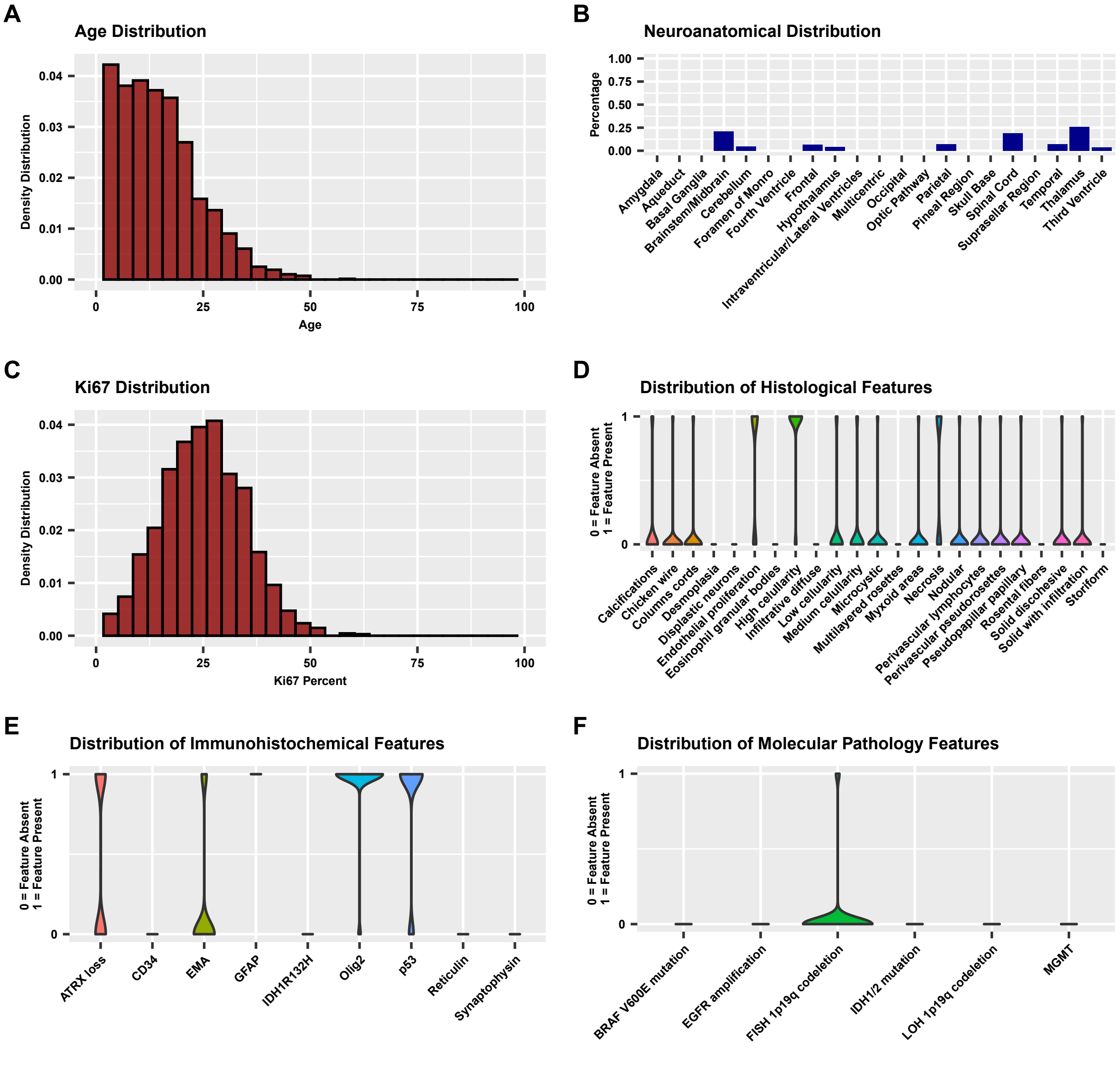

Figure S25. Diffuse Midline Glioma, WHO grade 4

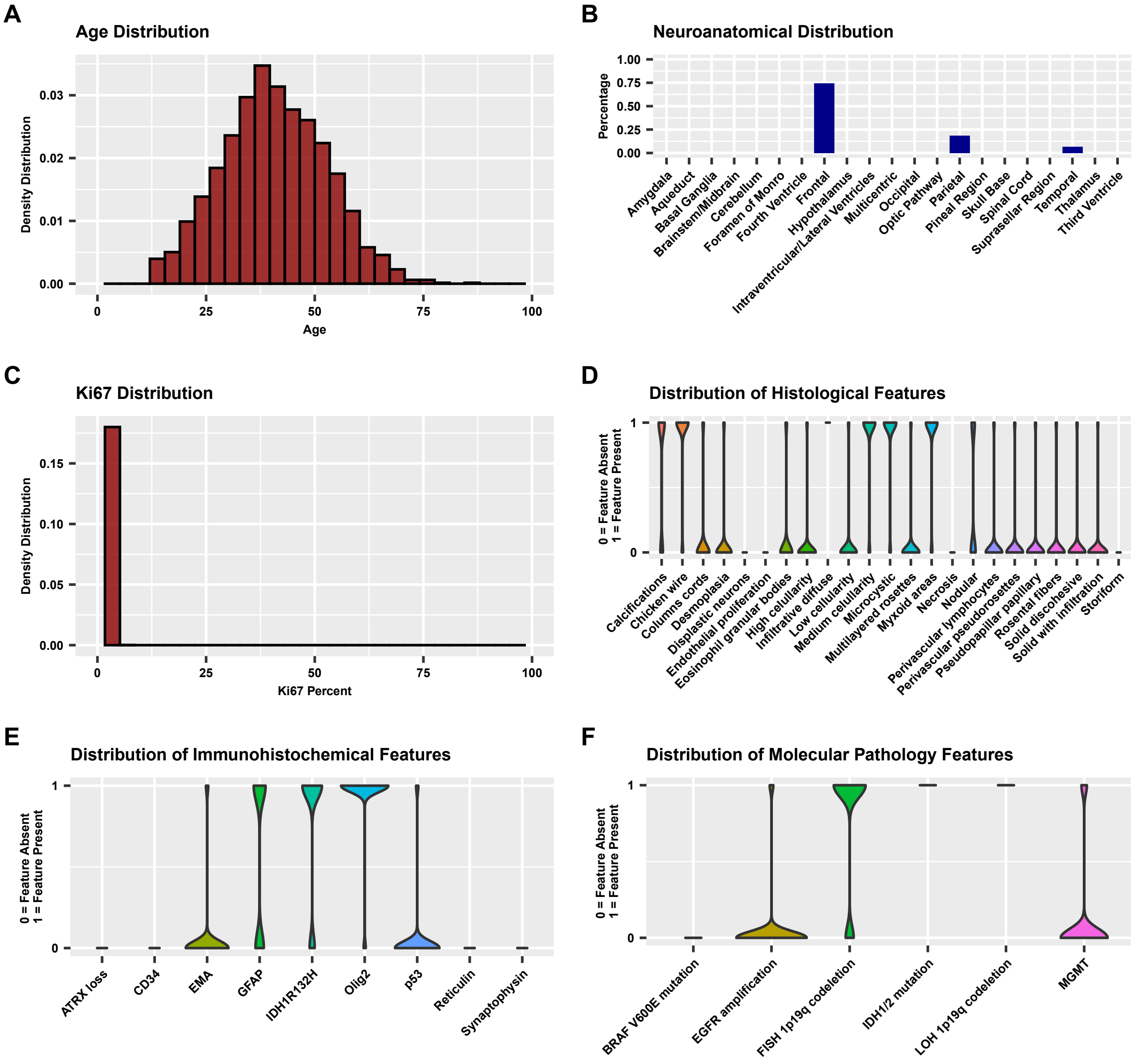

Figure S26. Diffuse Oligodendroglioma, WHO grade 2

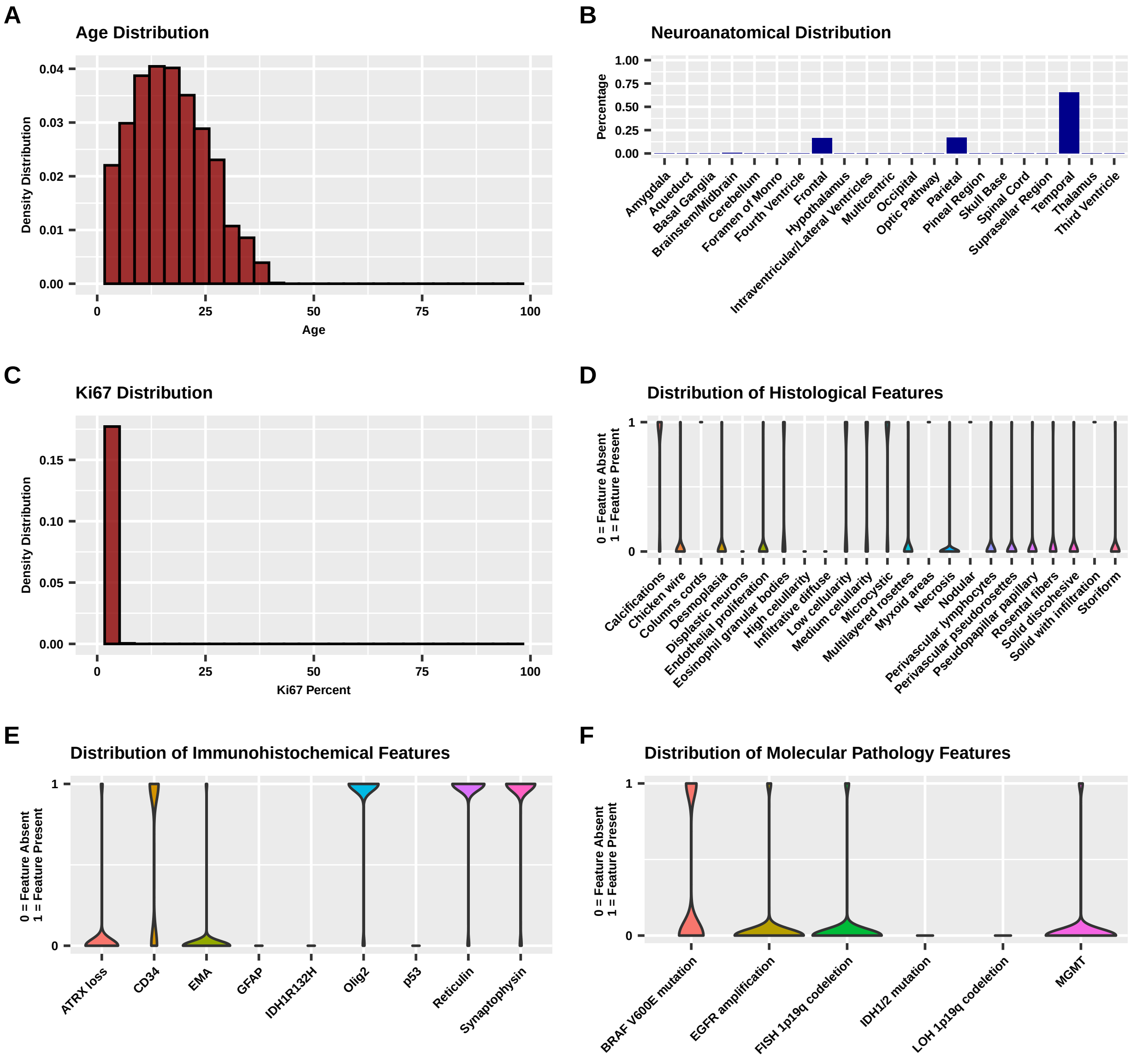

Figure S27. Dysembryoplastic Neuroepithelial Tumor

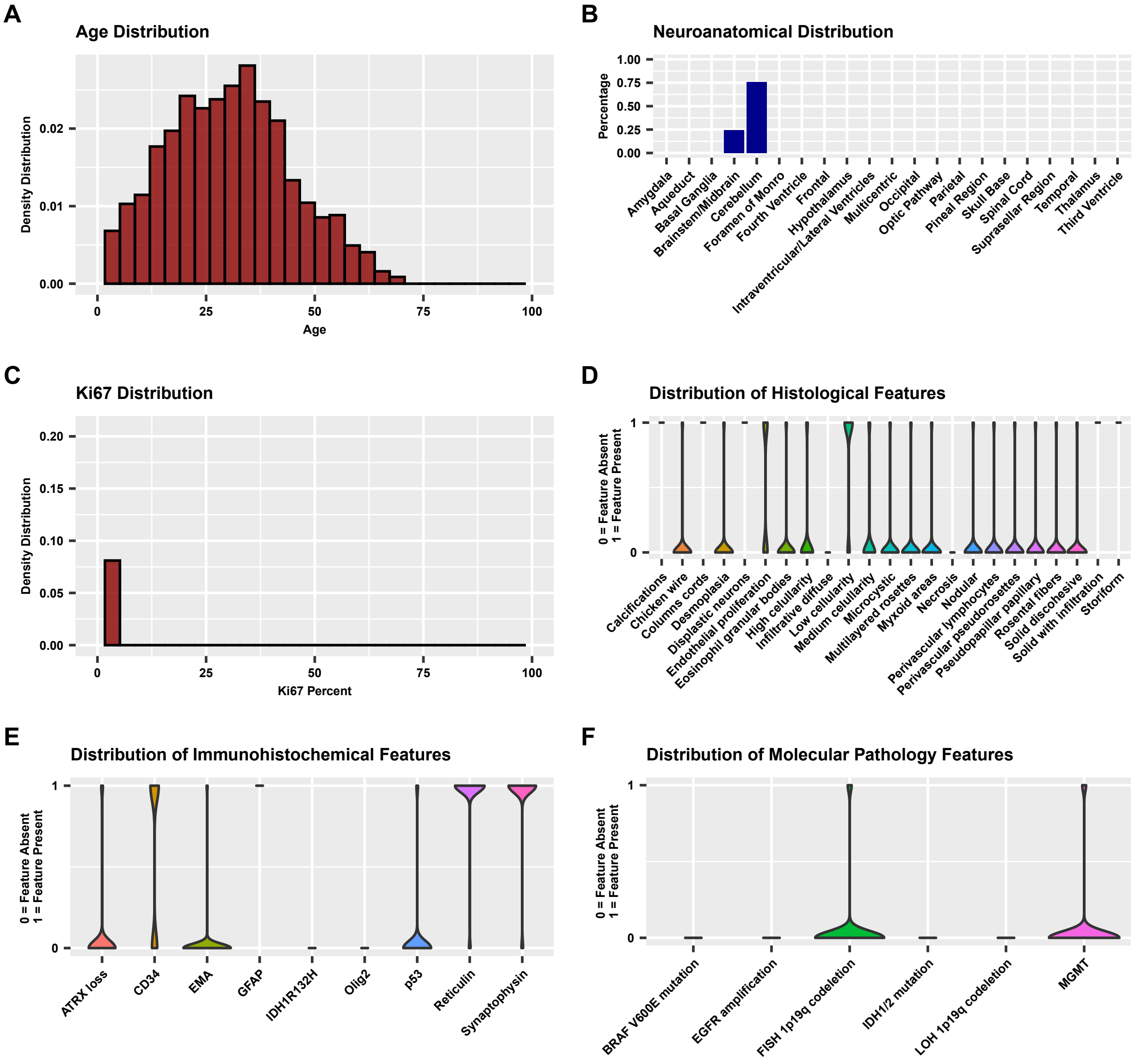

Figure S28. Dysplastic Cerebellar Gangliocytoma, WHO grade 1

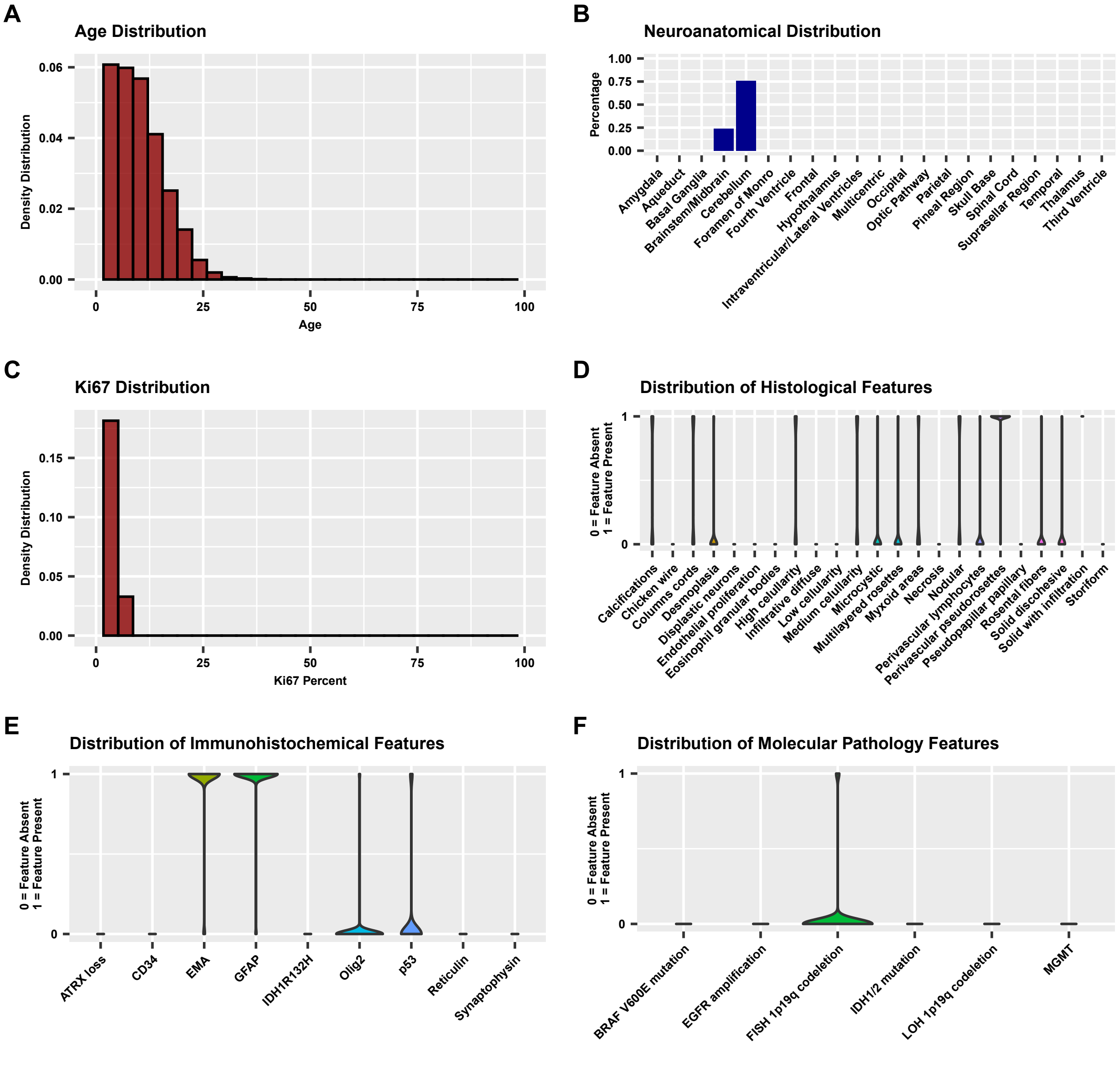

Figure S29. Ependymoma, Posterior Fossa A, WHO grade 2

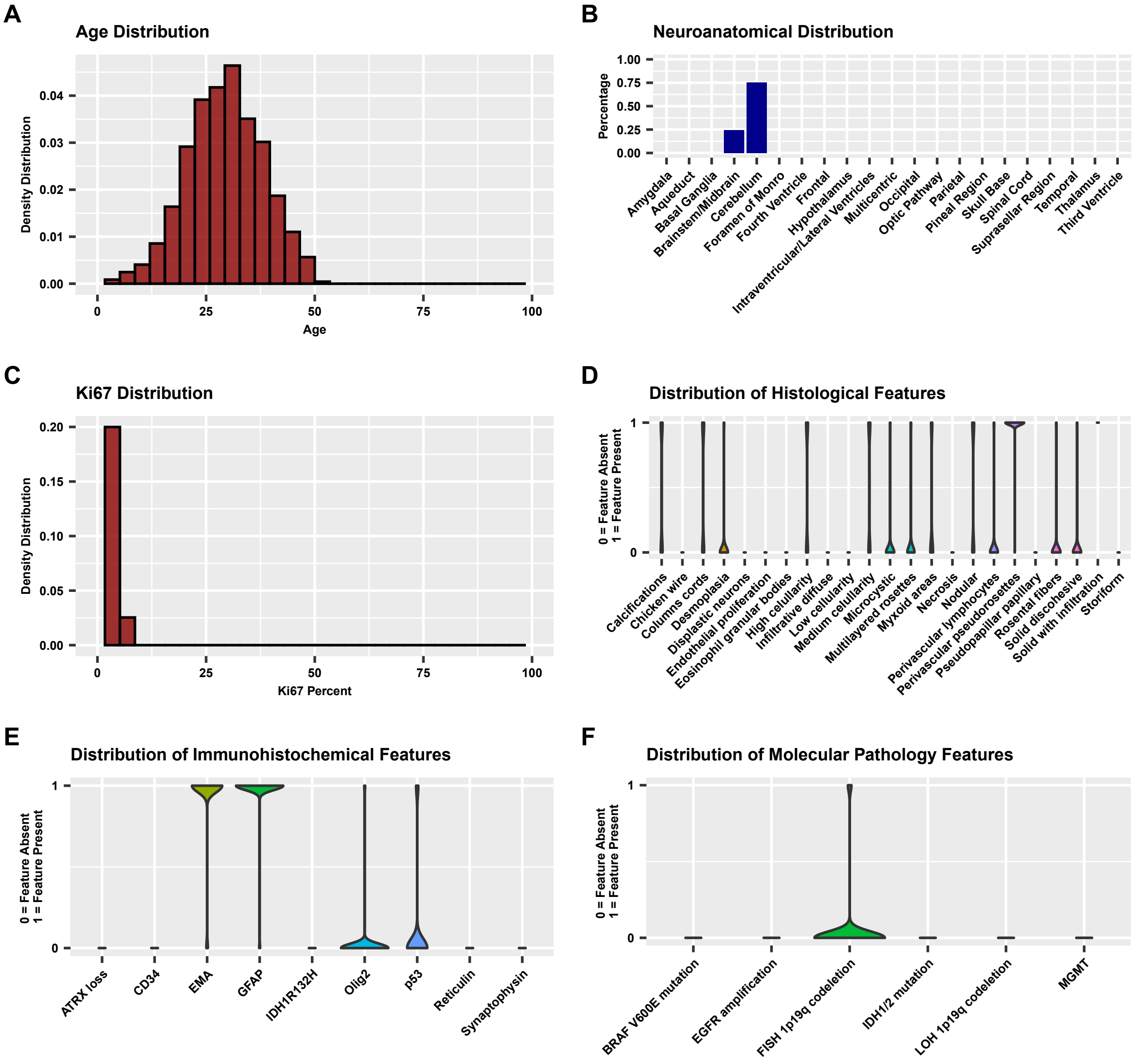

Figure S30. Ependymoma, Posterior Fossa B, WHO grade 2

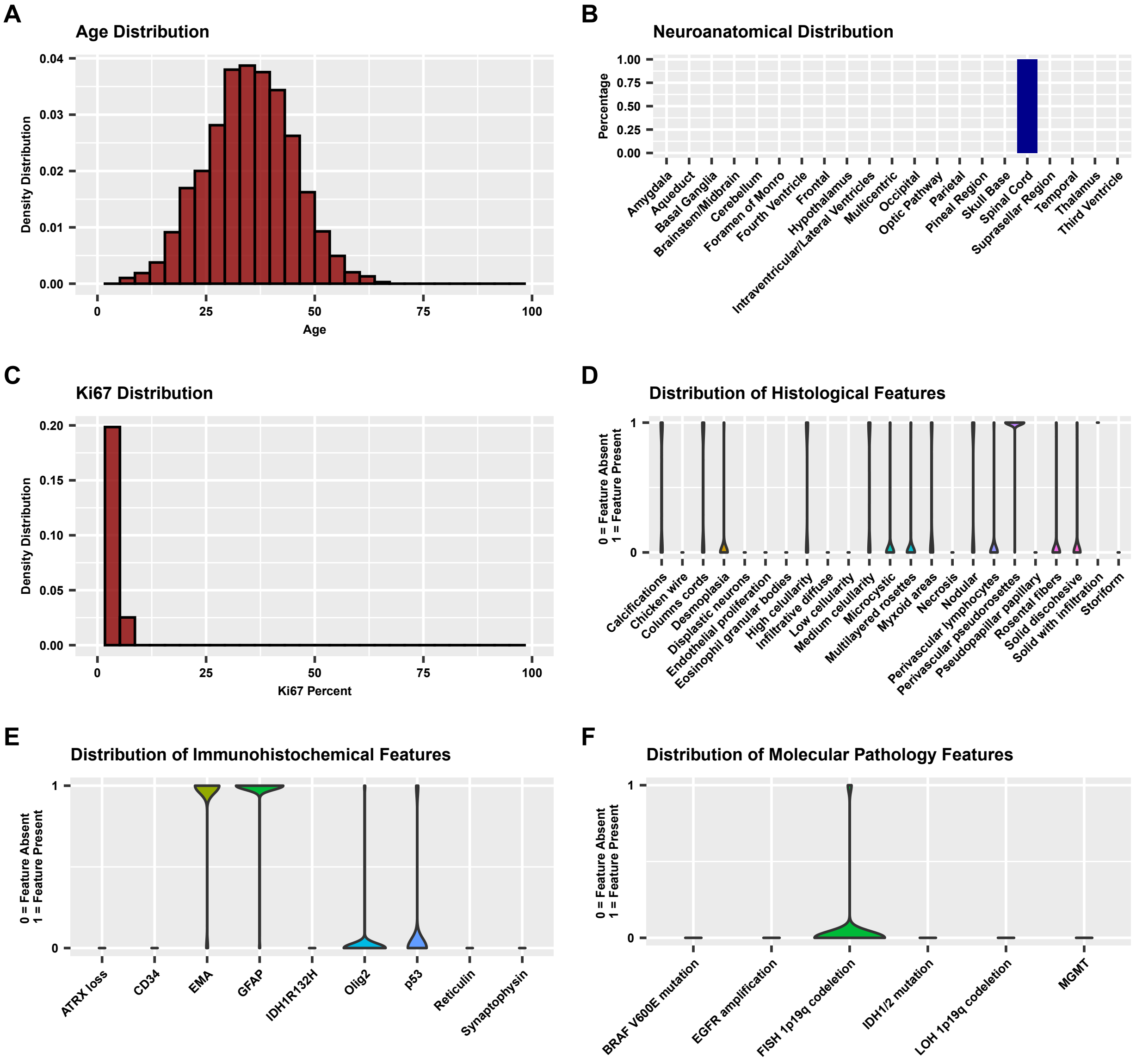

Figure S31. Ependymoma, Spine, WHO grade 2

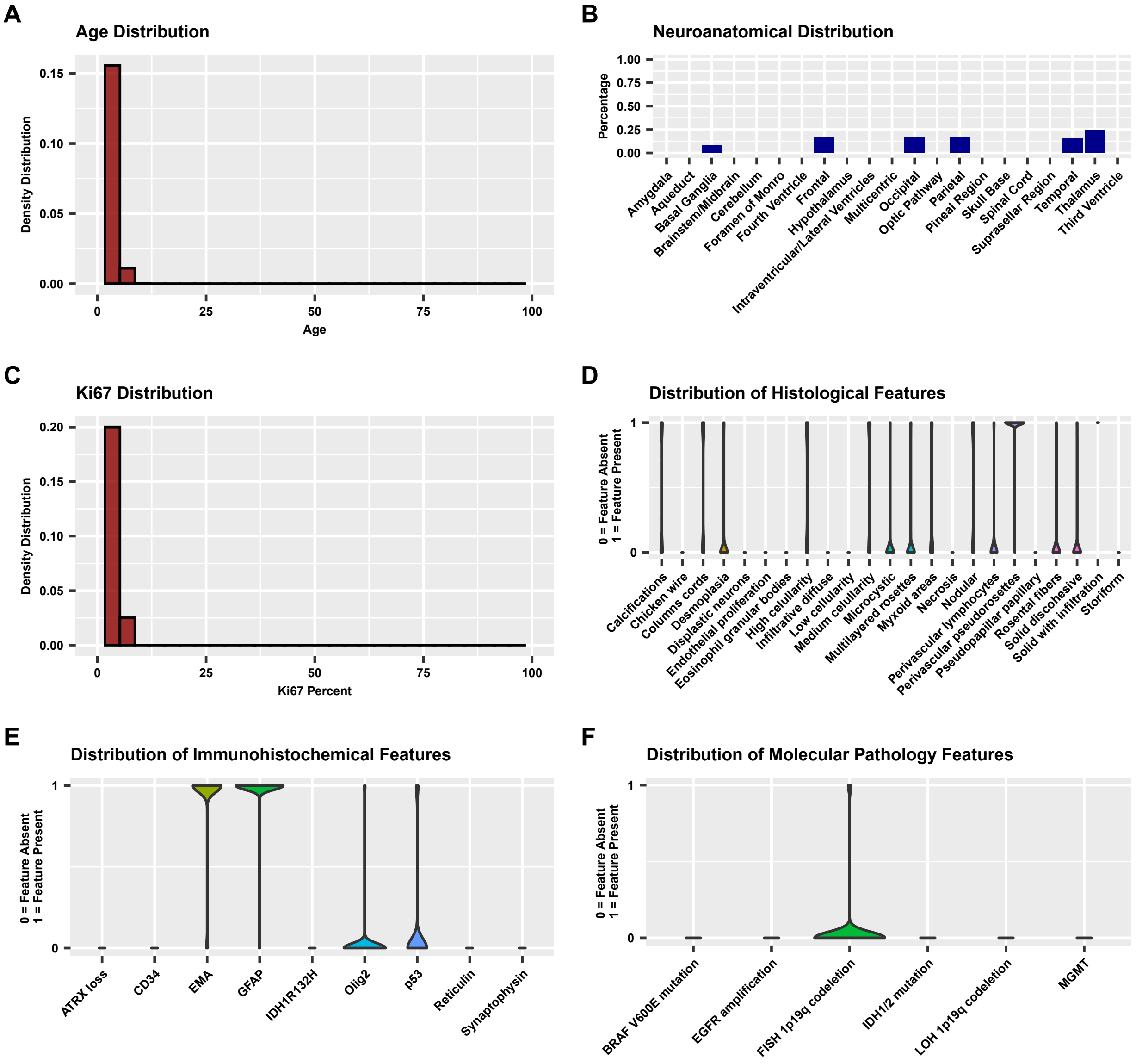

Figure S32. Ependymoma, Supratentorial−YAP, WHO grade 2

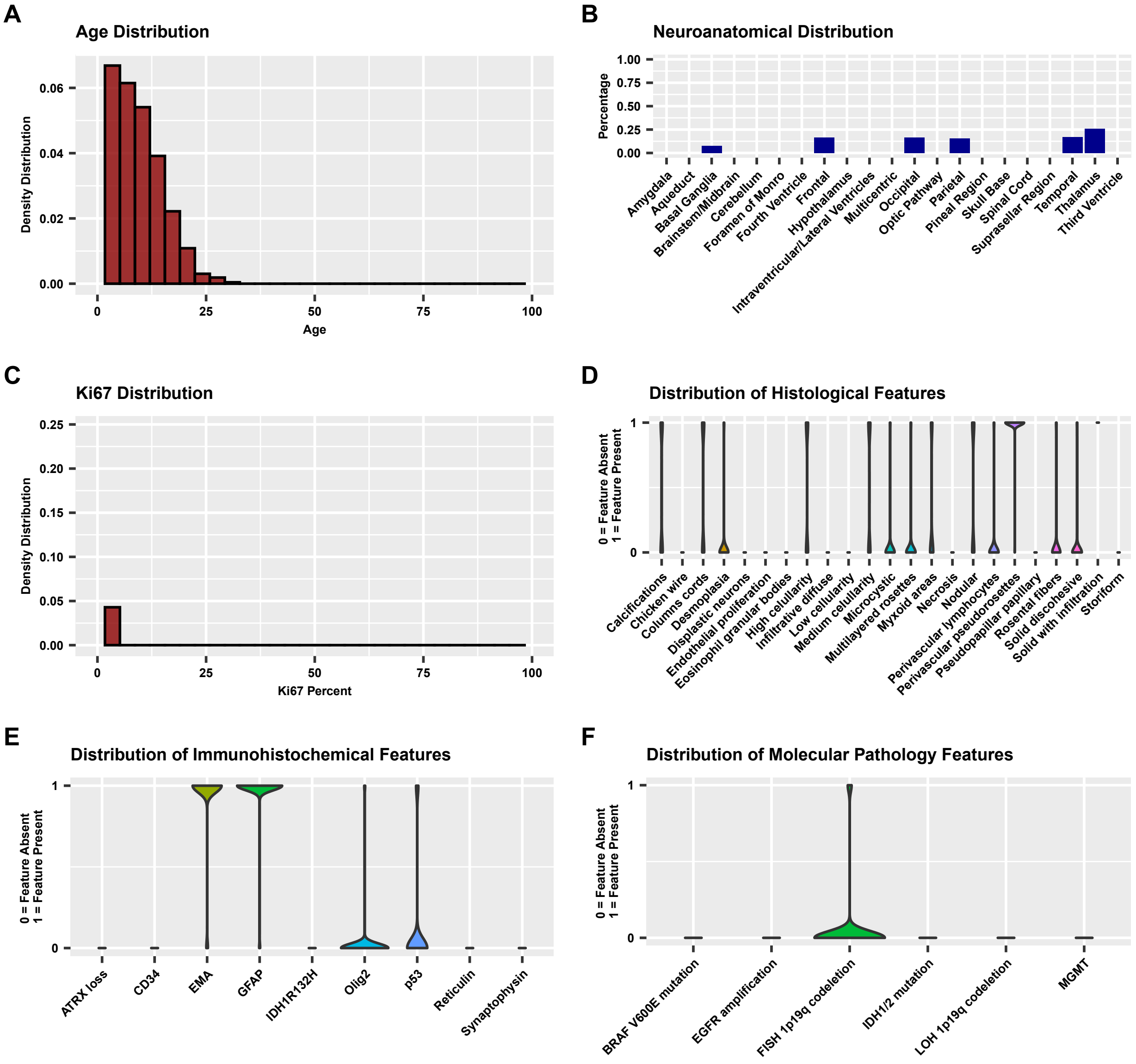

Figure S33. Ependymoma, Supratentorial, RELA, WHO grade 2

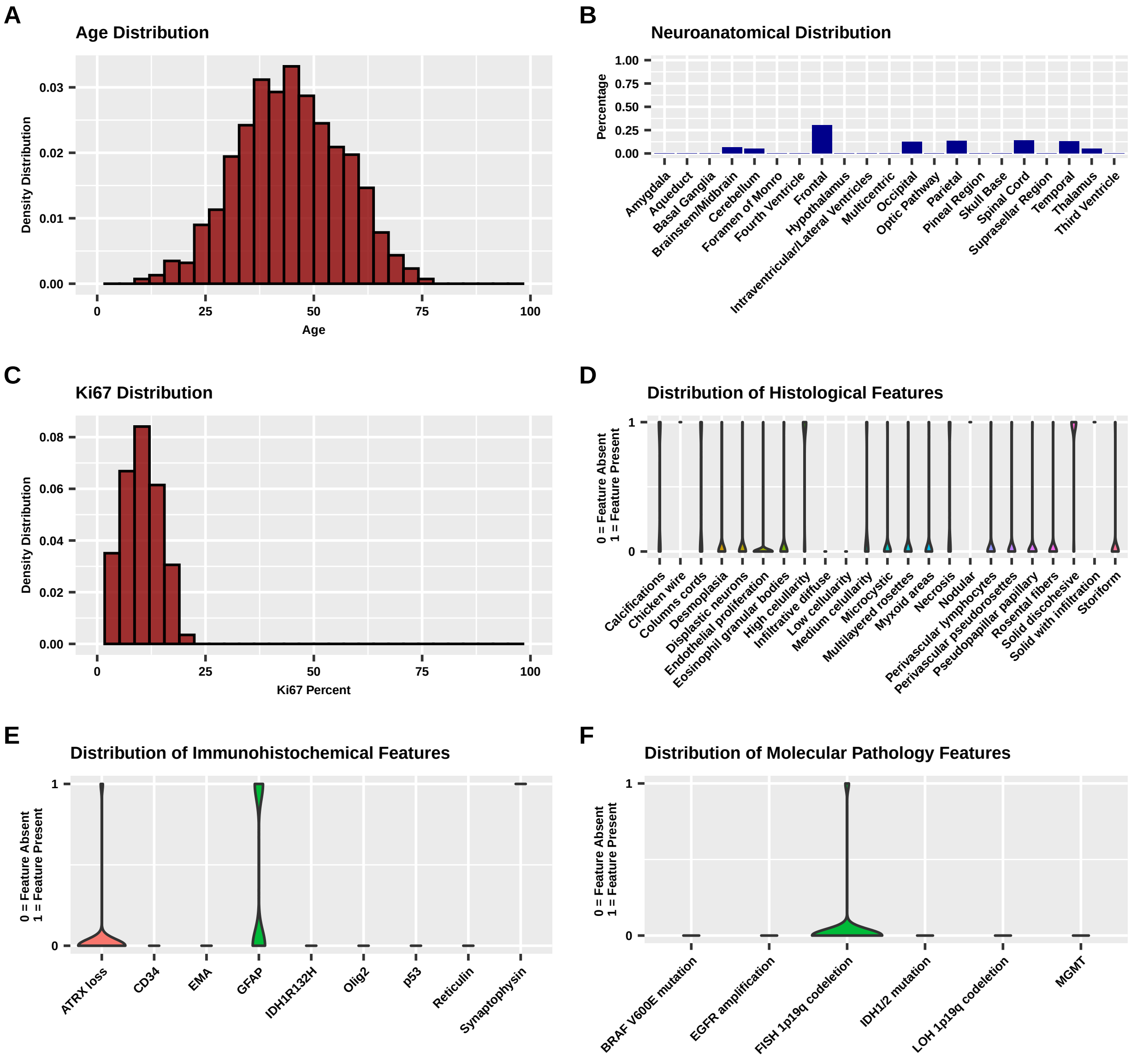

Figure S34. Extraventricular Neurocytoma

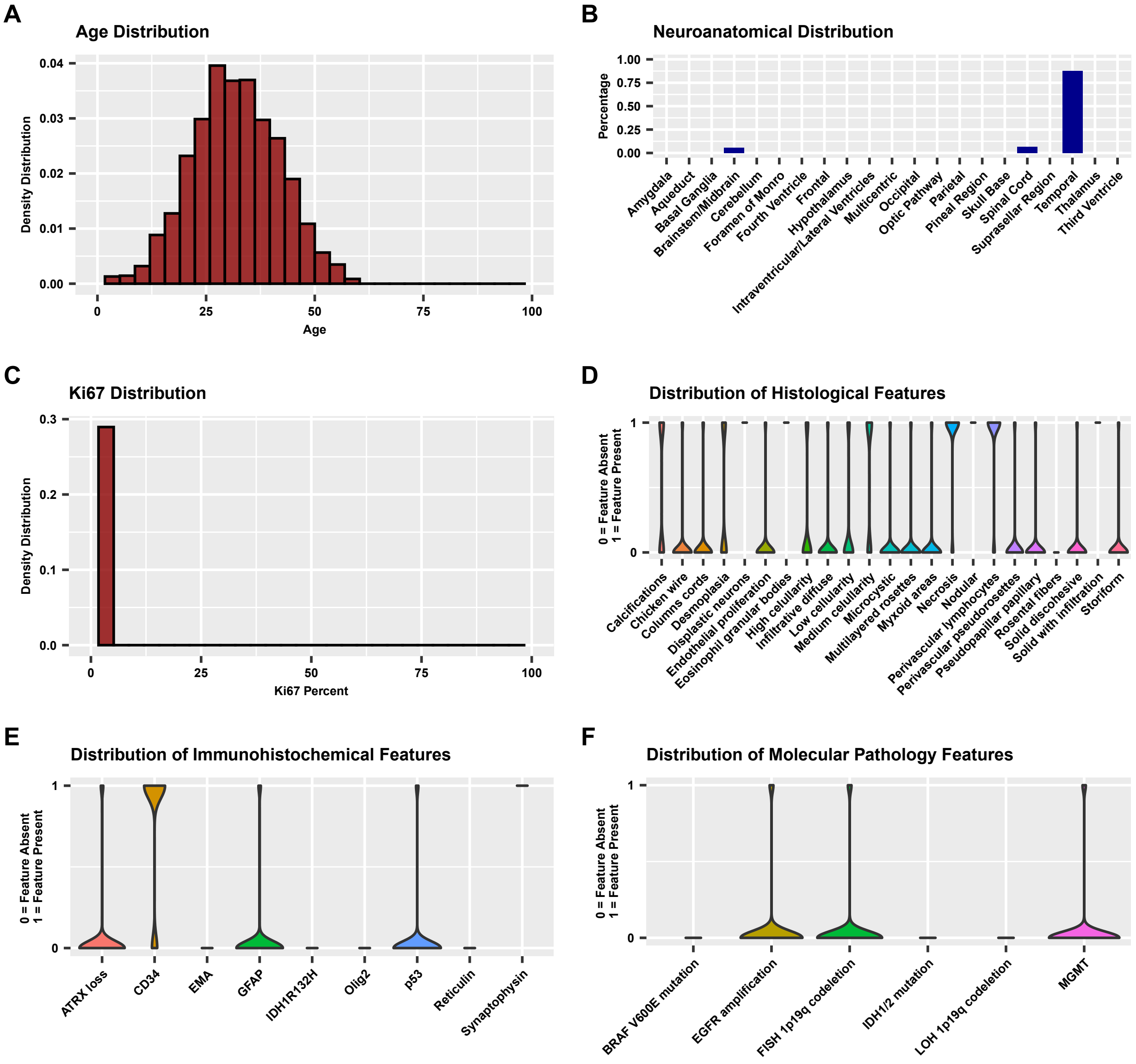

Figure S35. Gangliocytoma, WHO grade 1

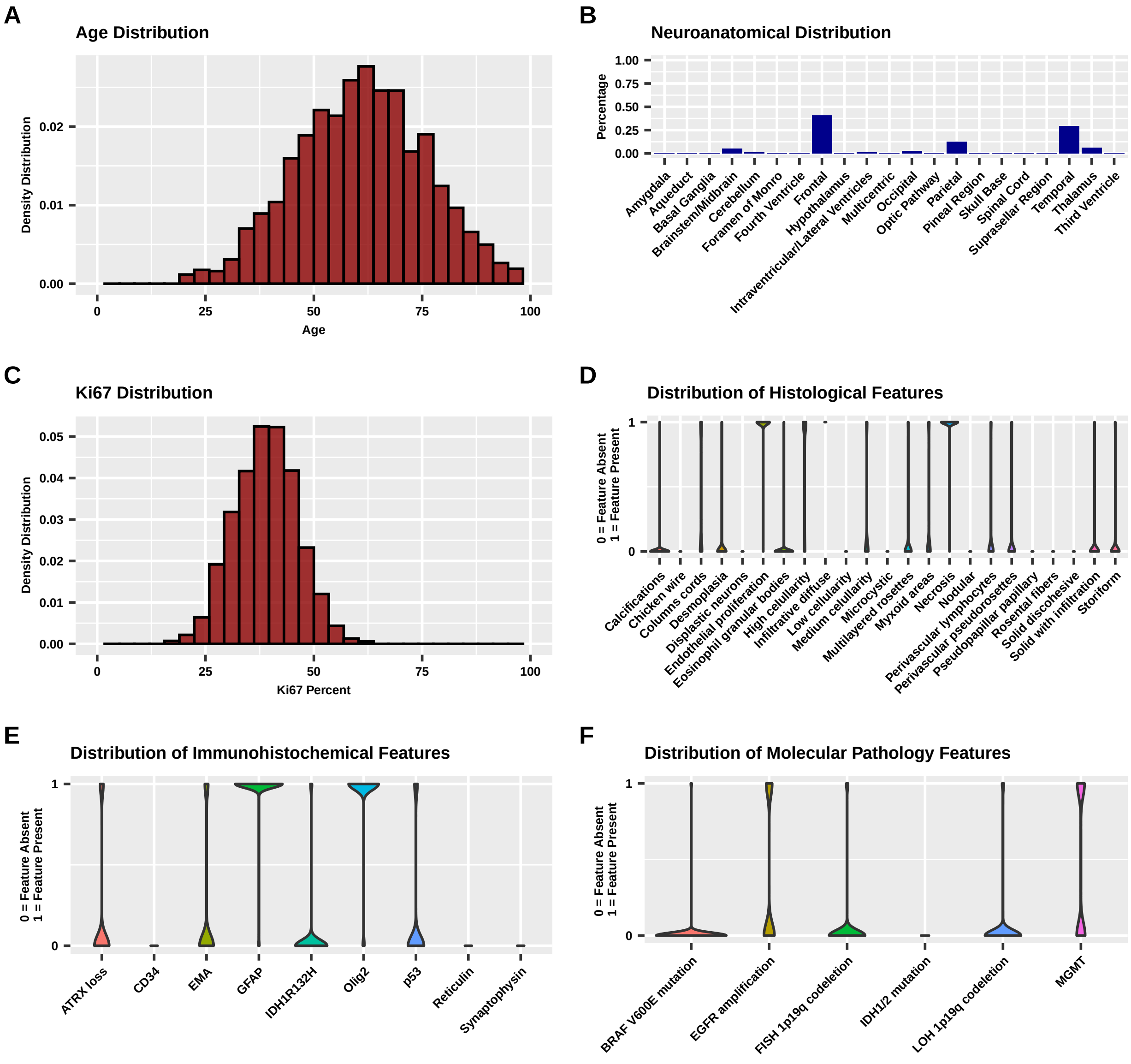

Figure S36. Glioblastoma

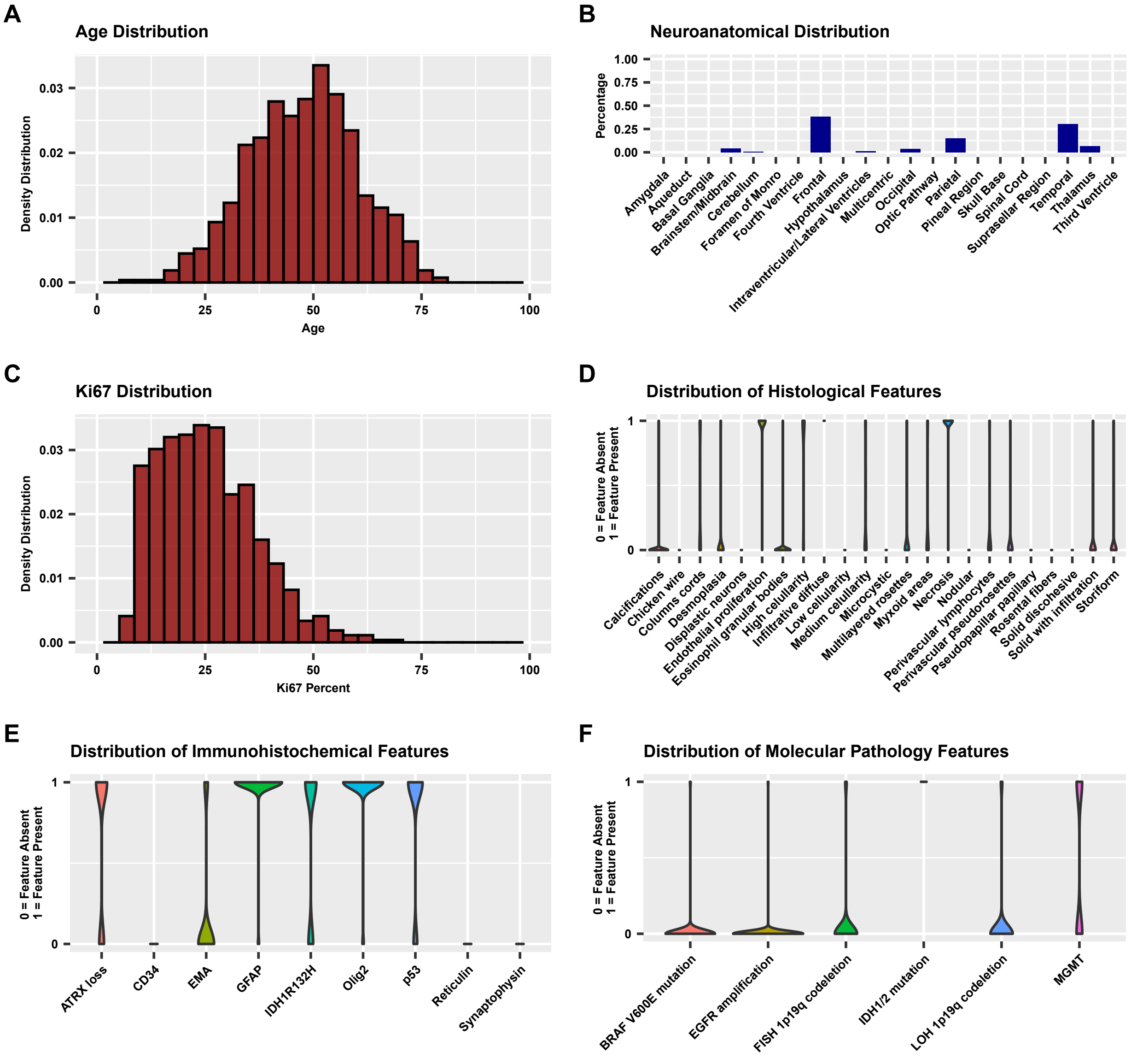

Figure S37. Glioblastoma, IDH−mutant, WHO grade 4

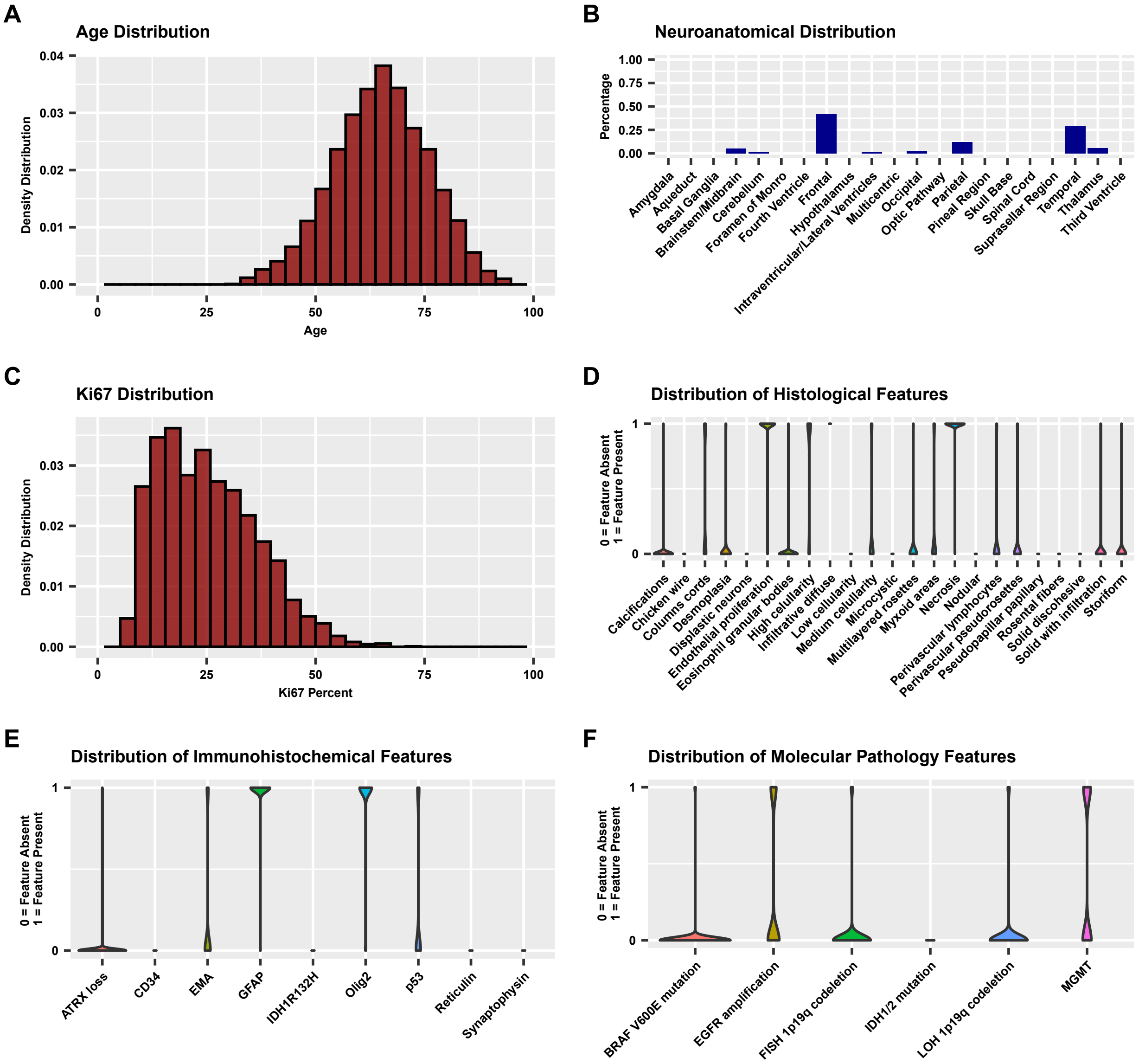

Figure S38. Glioblastoma, IDH−wild type, WHO grade 4

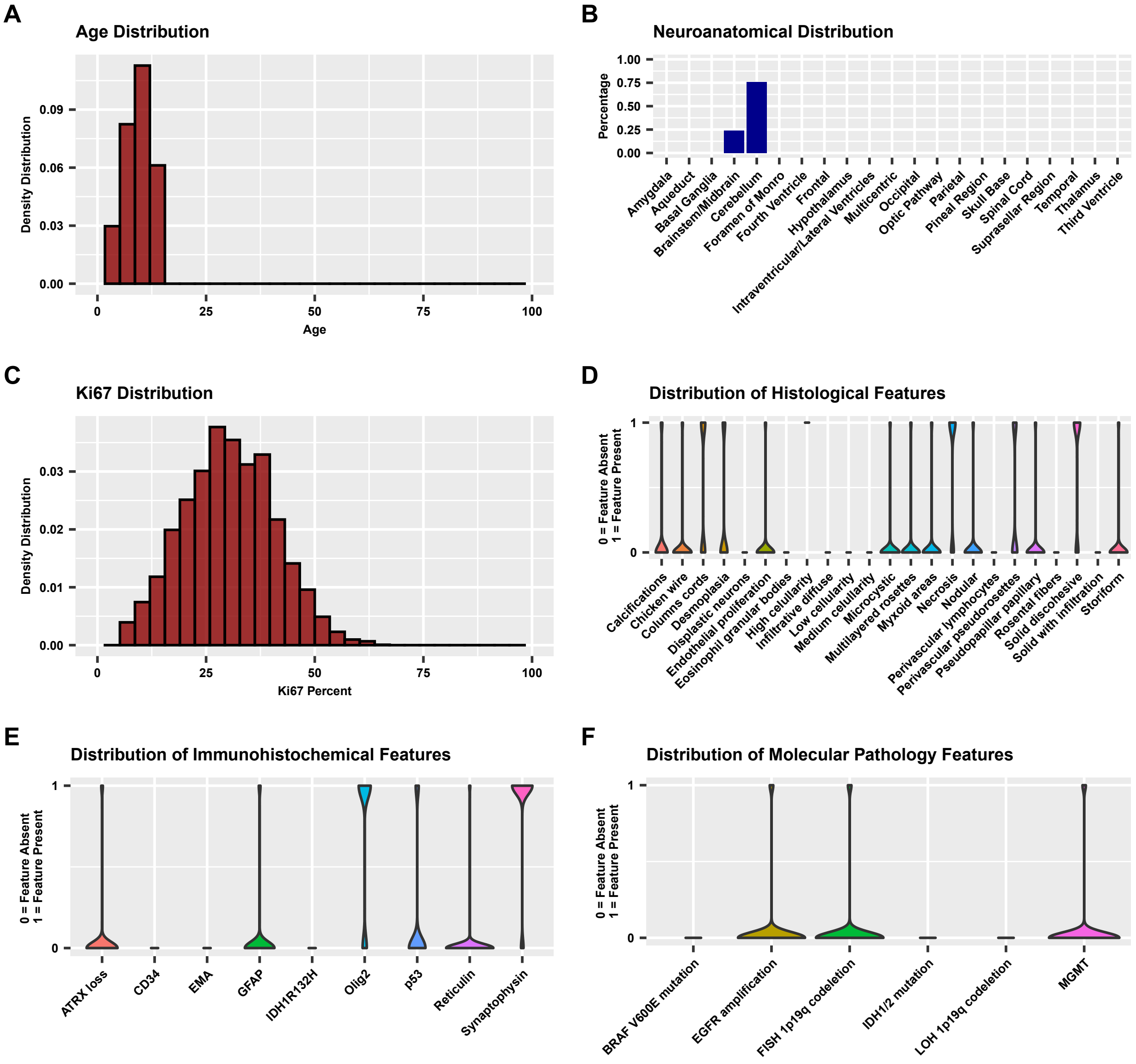

Figure S39. Medulloblastoma, non−WNT/non−SHH, WHO grade 4

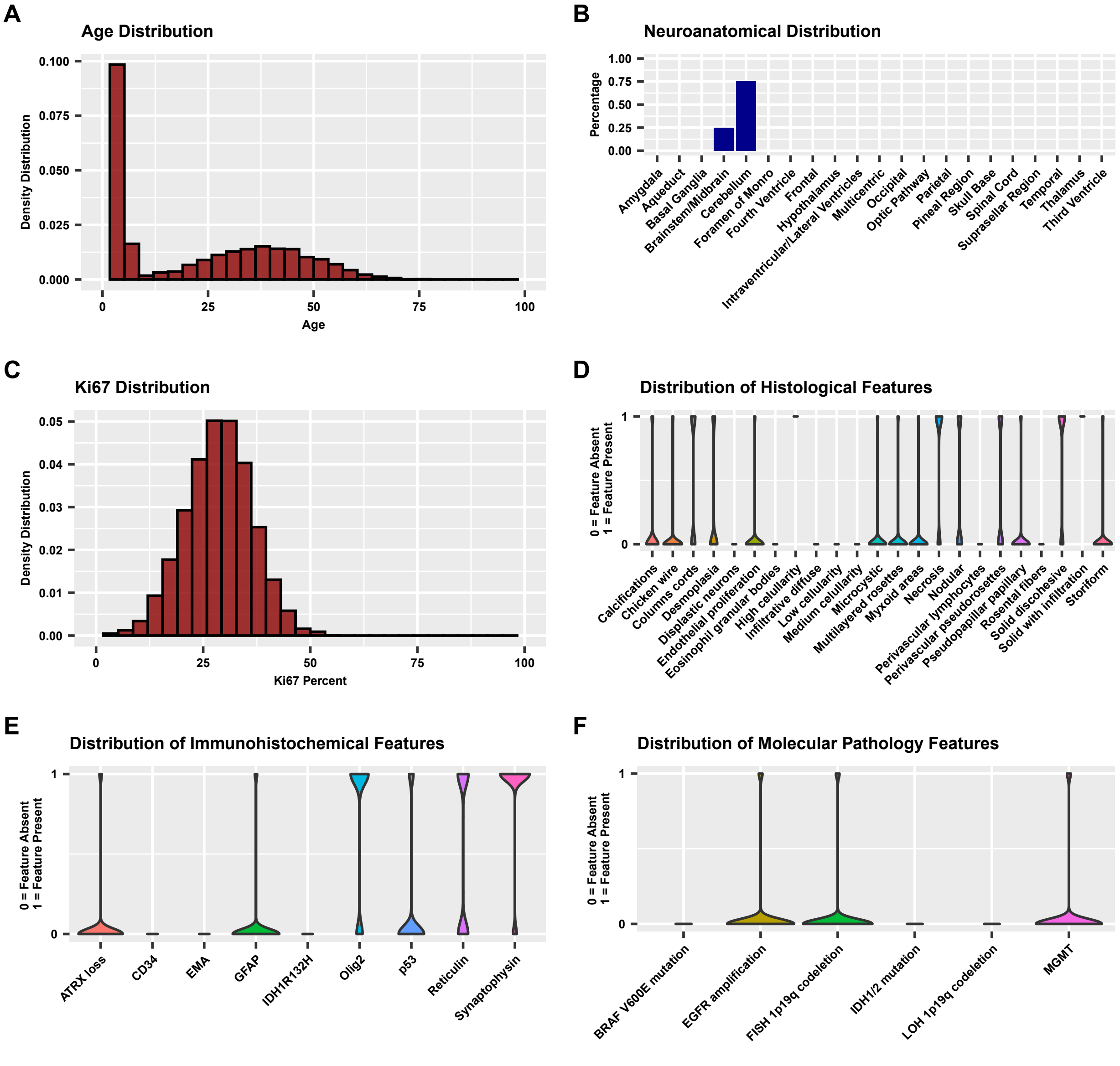

Figure S40. Medulloblastoma, SHH Class, WHO grade 4

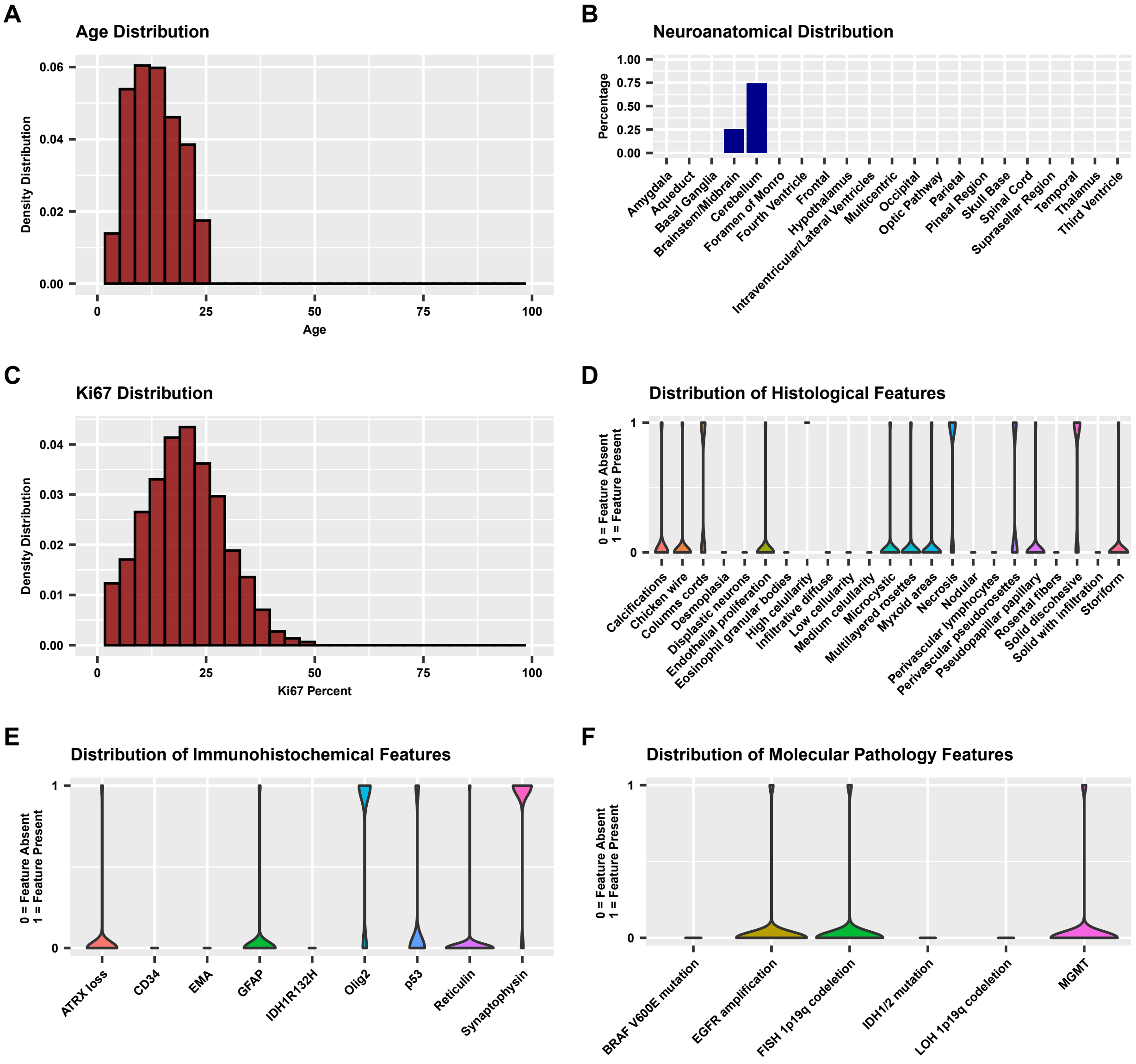

Figure S41. Medulloblastoma, WNT Group, WHO grade 4

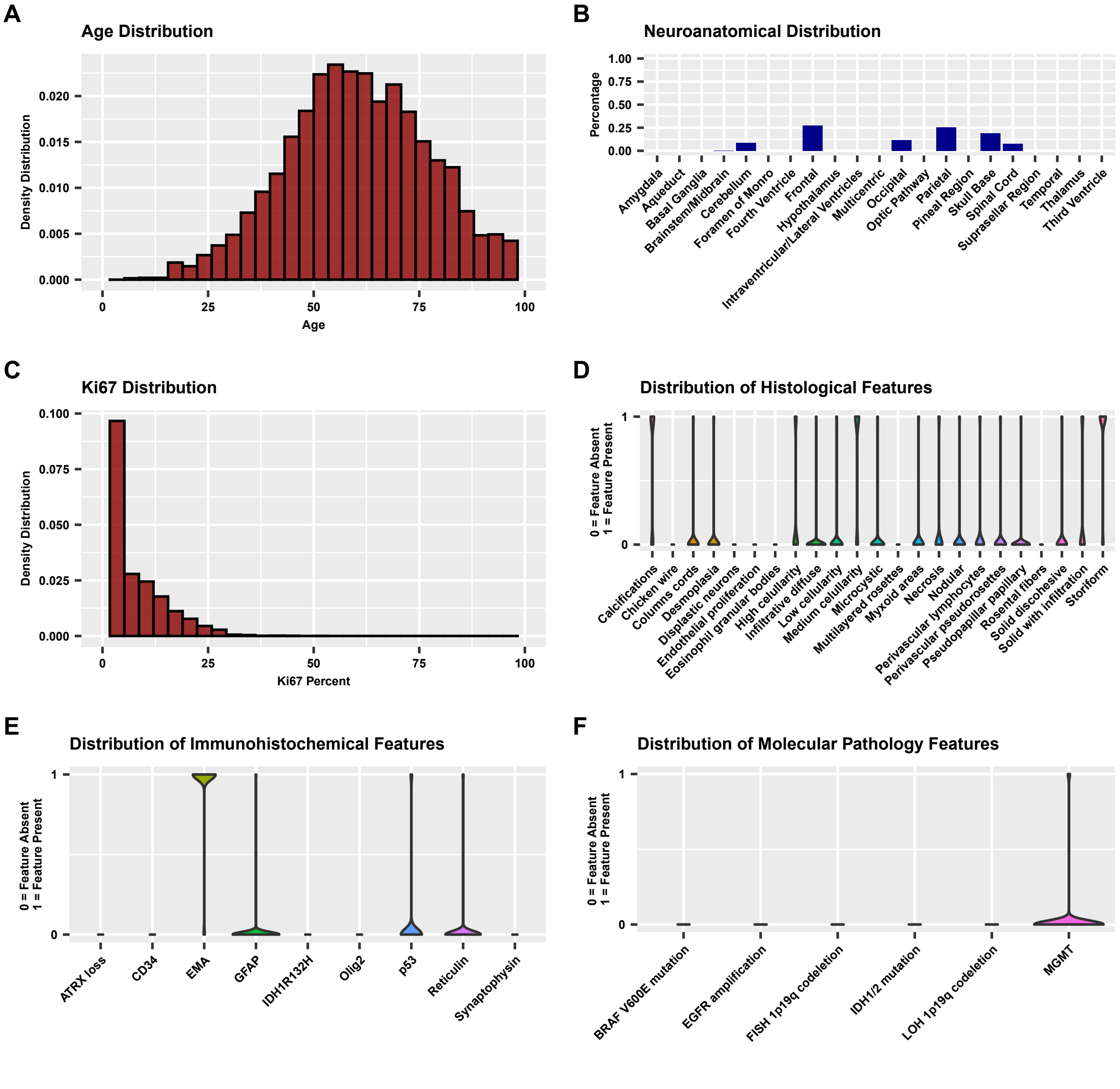

Figure S42. Meningioma,WHO grade 1

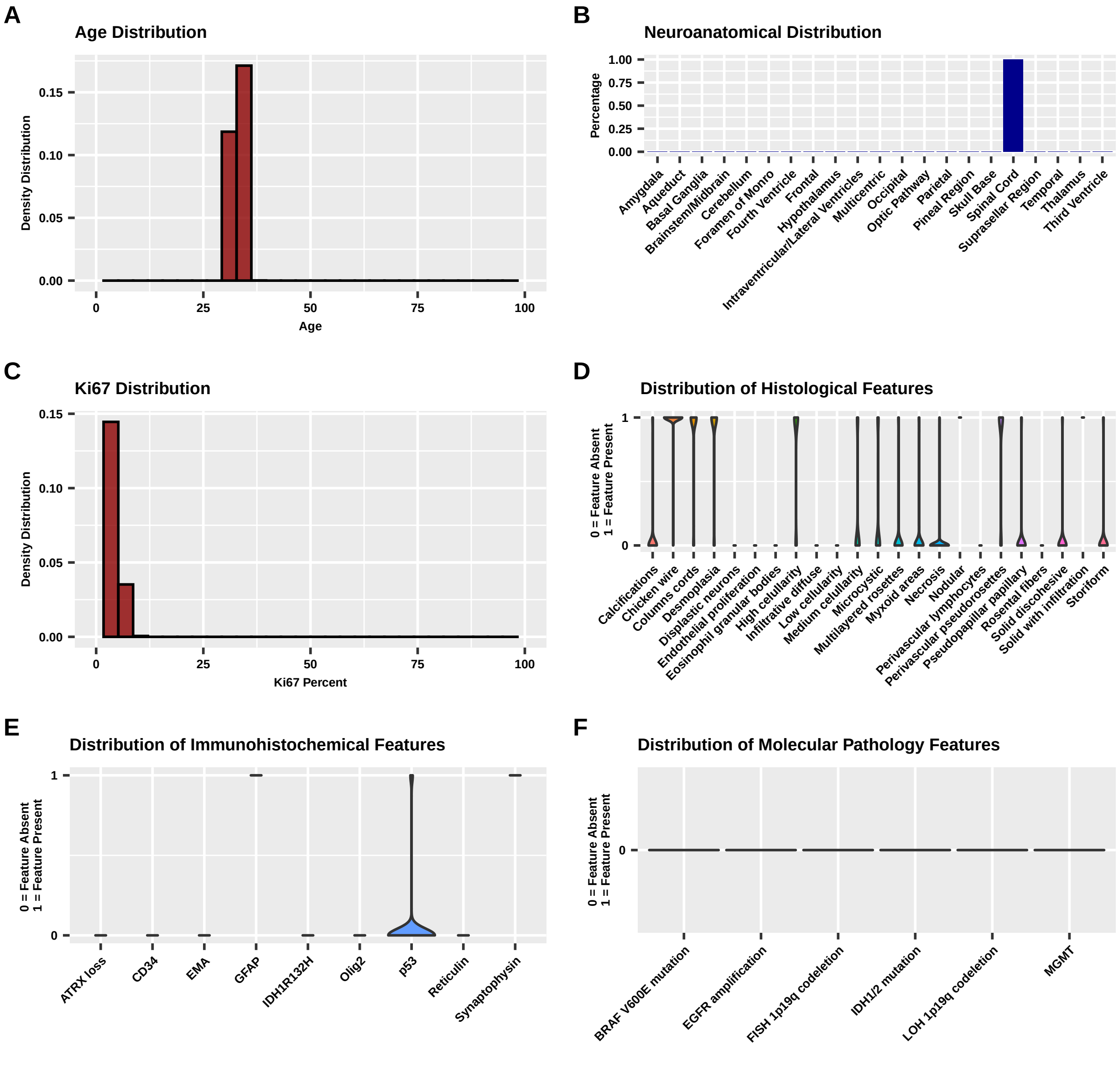

Figure S43. Paraganglioma

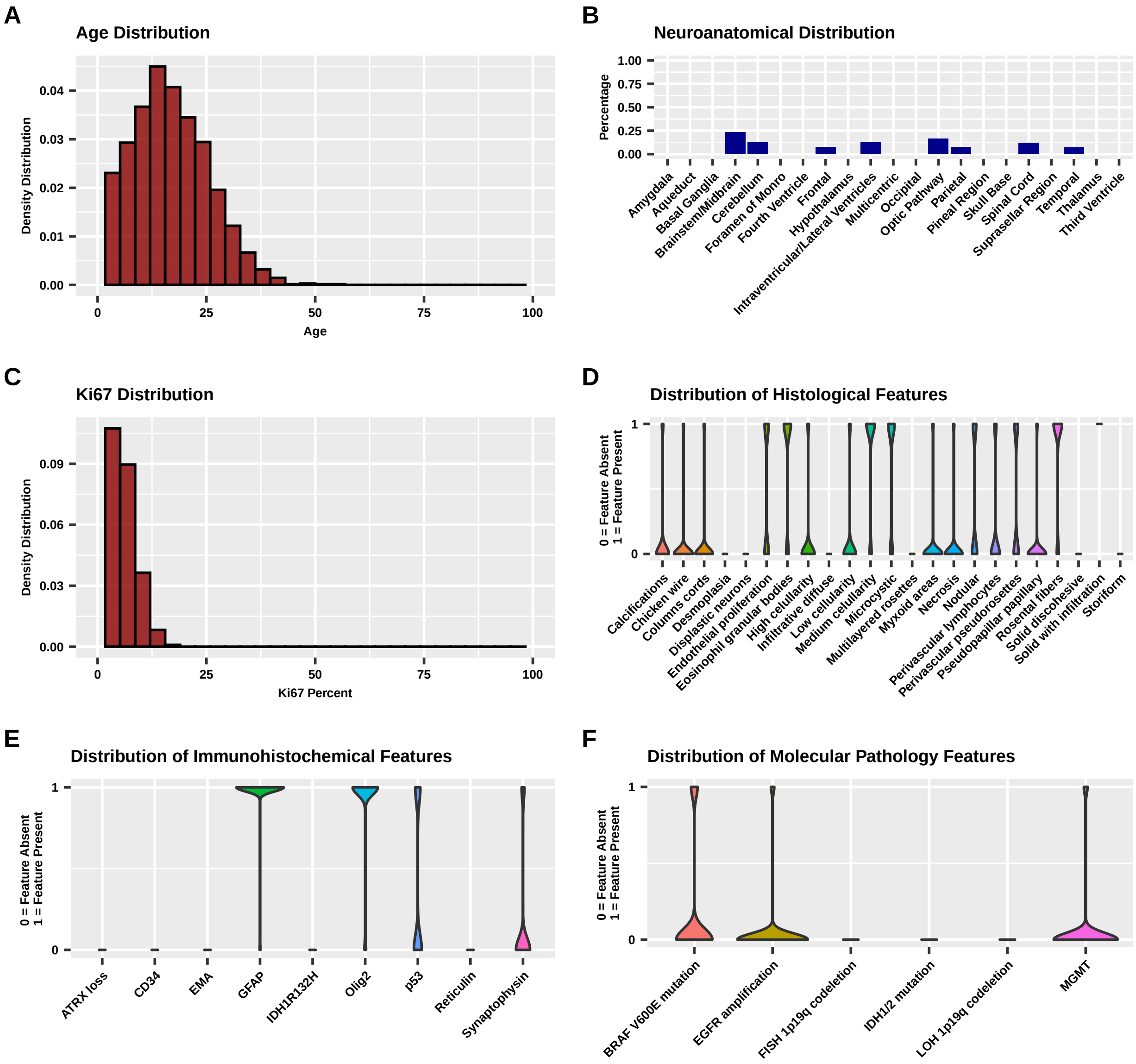

Figure S44. Pilocytic Astrocytoma

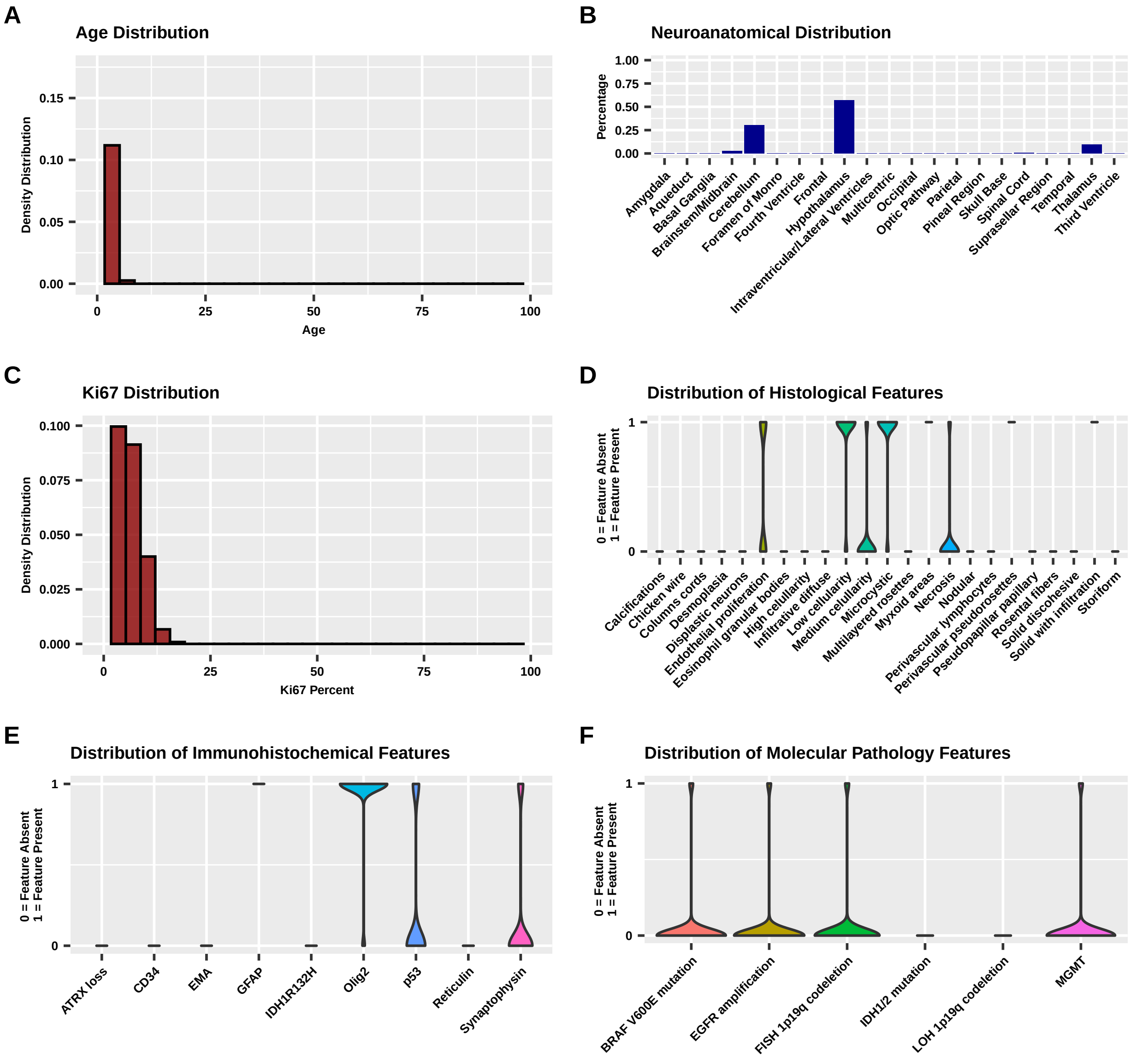

Figure S45. Pilomyxoid Astrocytoma

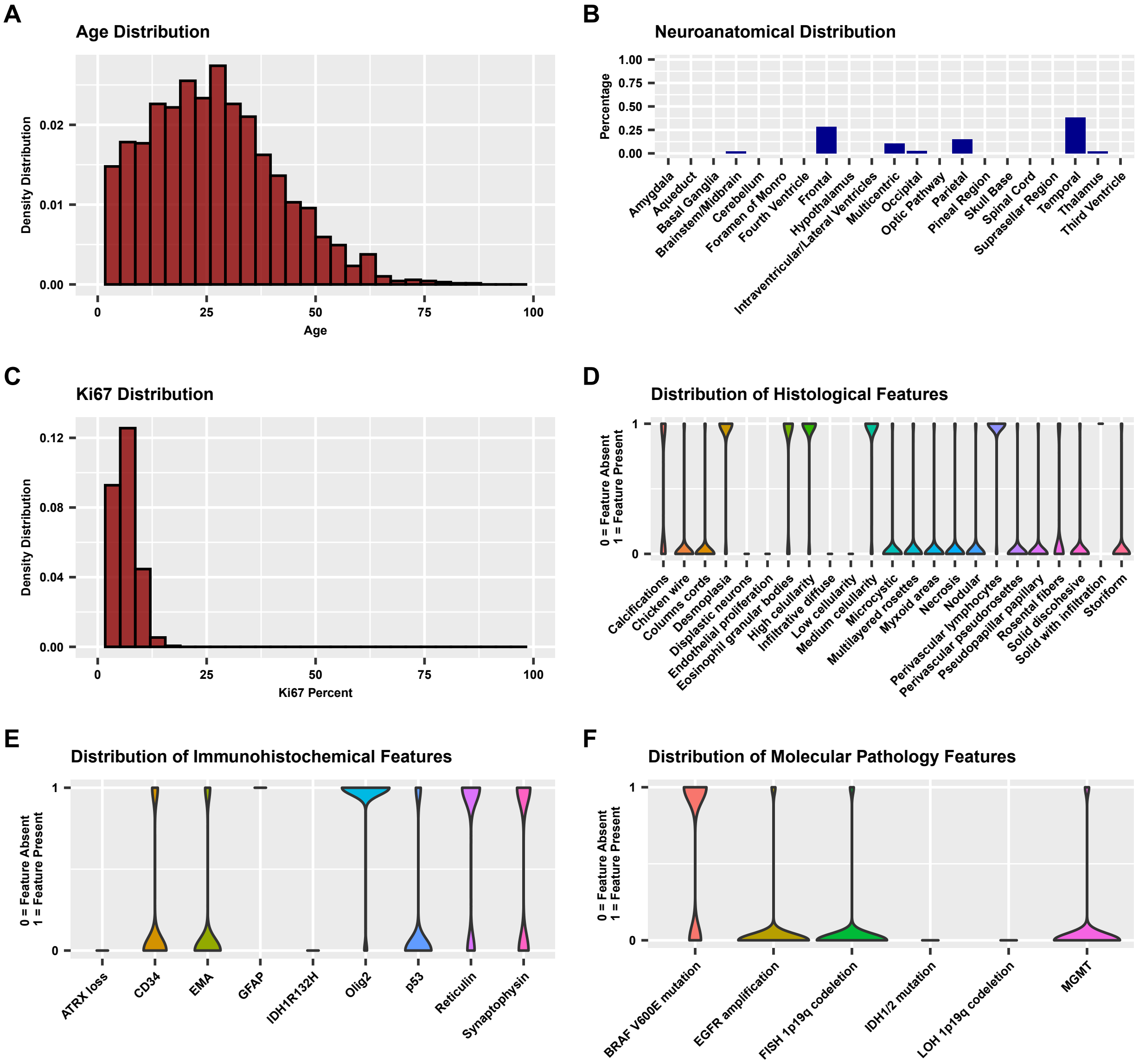

Figure S46. Pleomorphic Xanthoastrocytoma, WHO grade 2

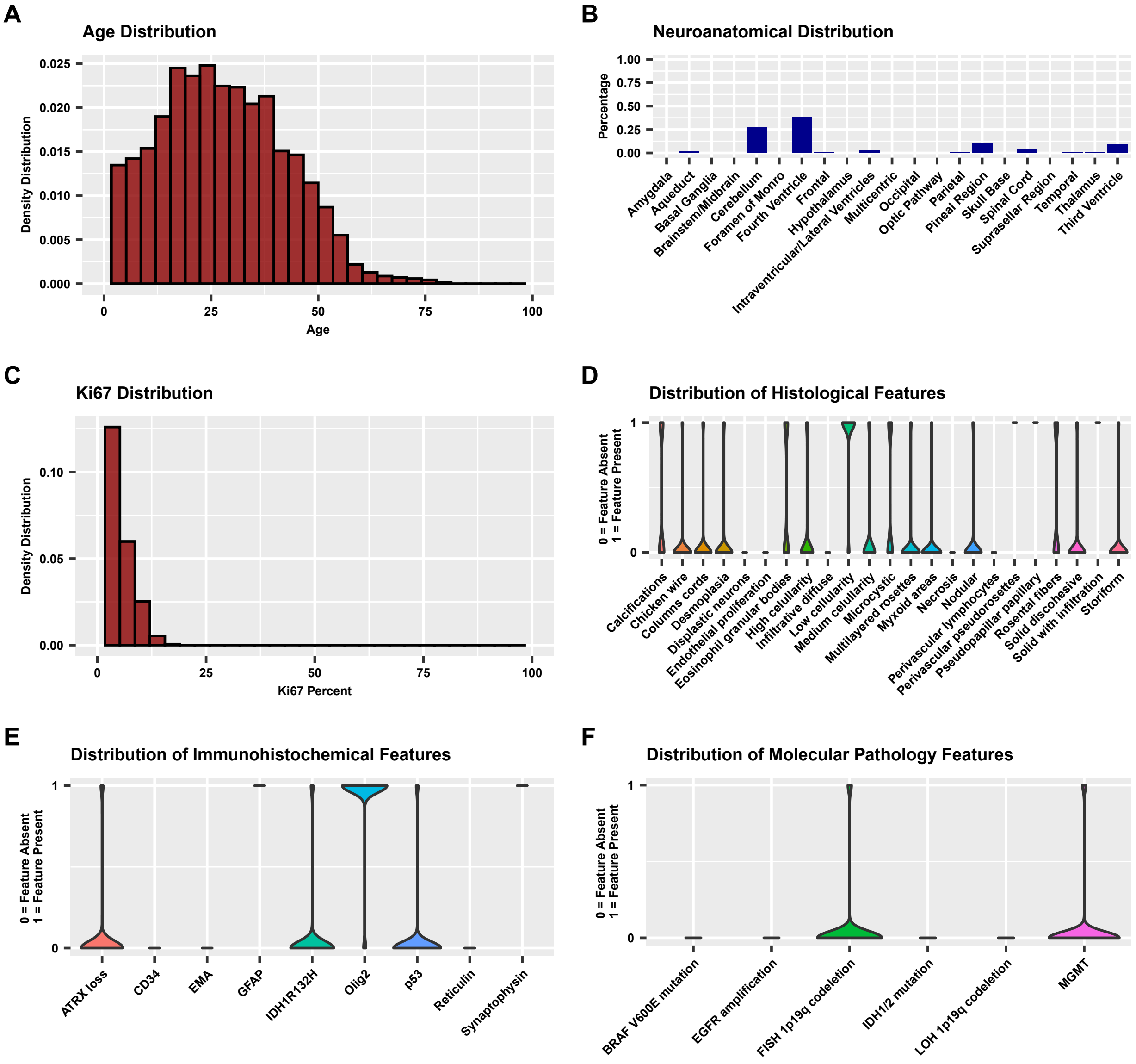

Figure S47. Rossete−forming Glioneuronl Tumour, WHO grade 1

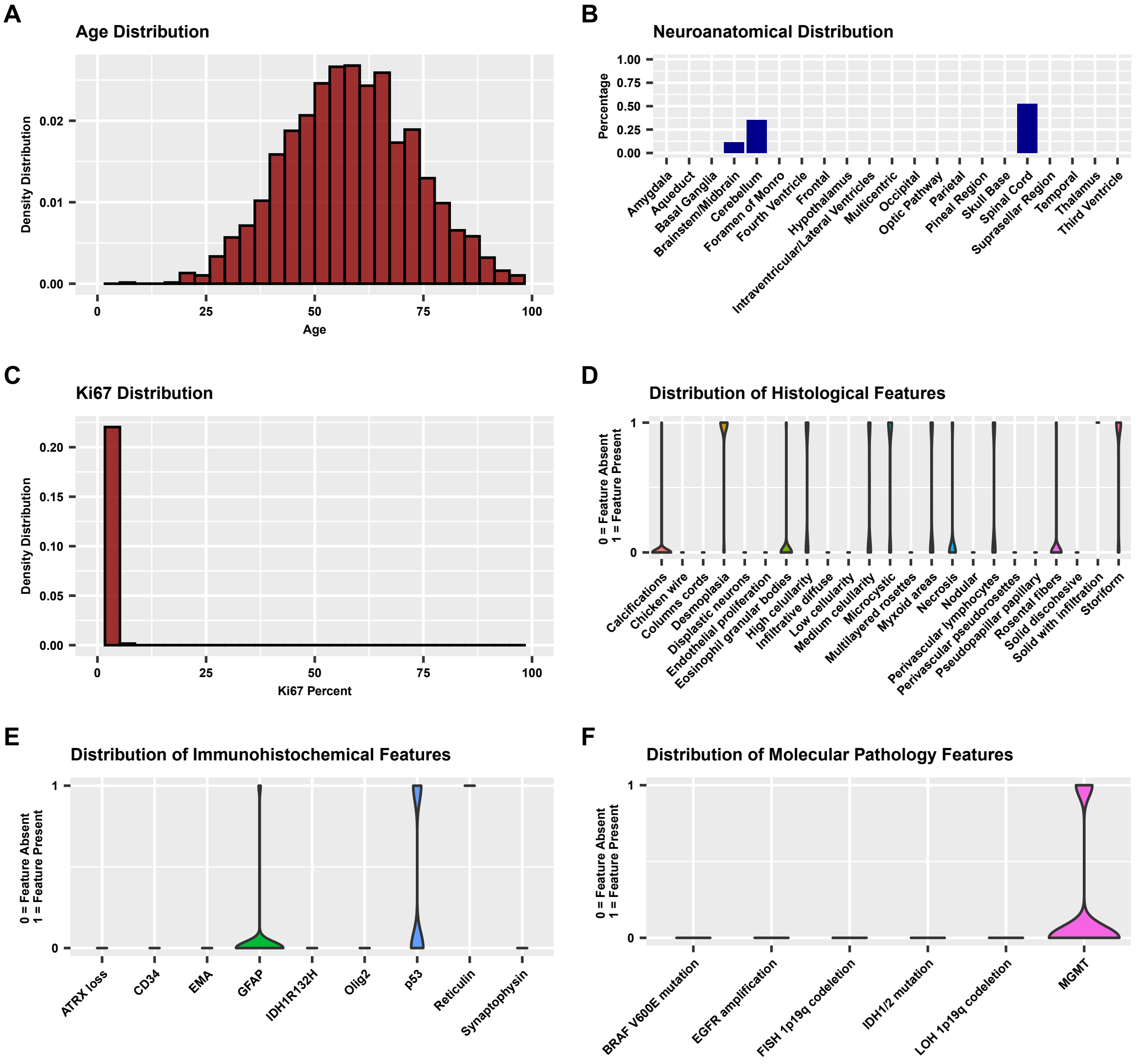

Figure S48. Schwannoma, WHO grade 1

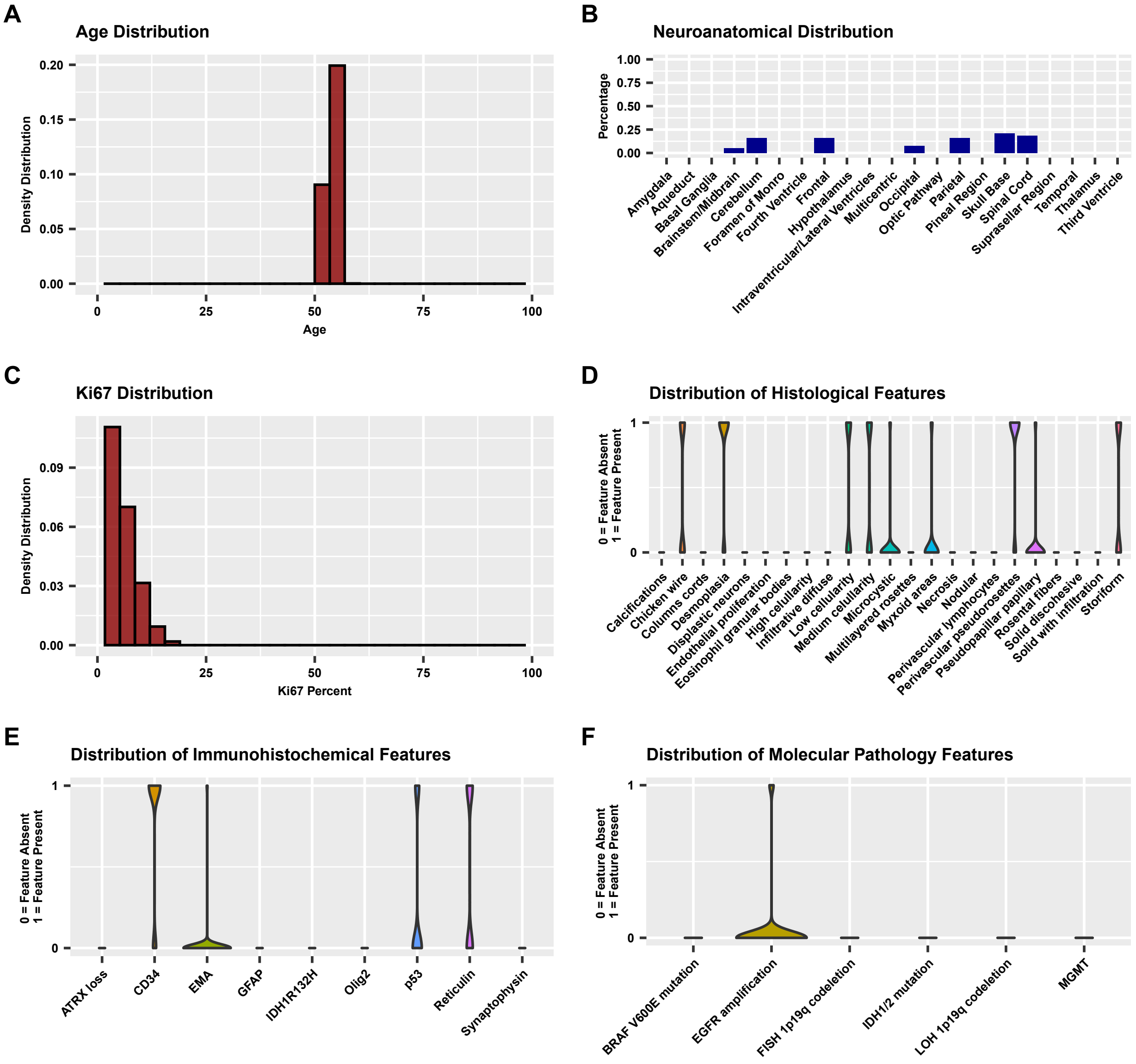

Figure S49. Solitary Fibrous Tumour/Hemangiopericytoma Grade 1

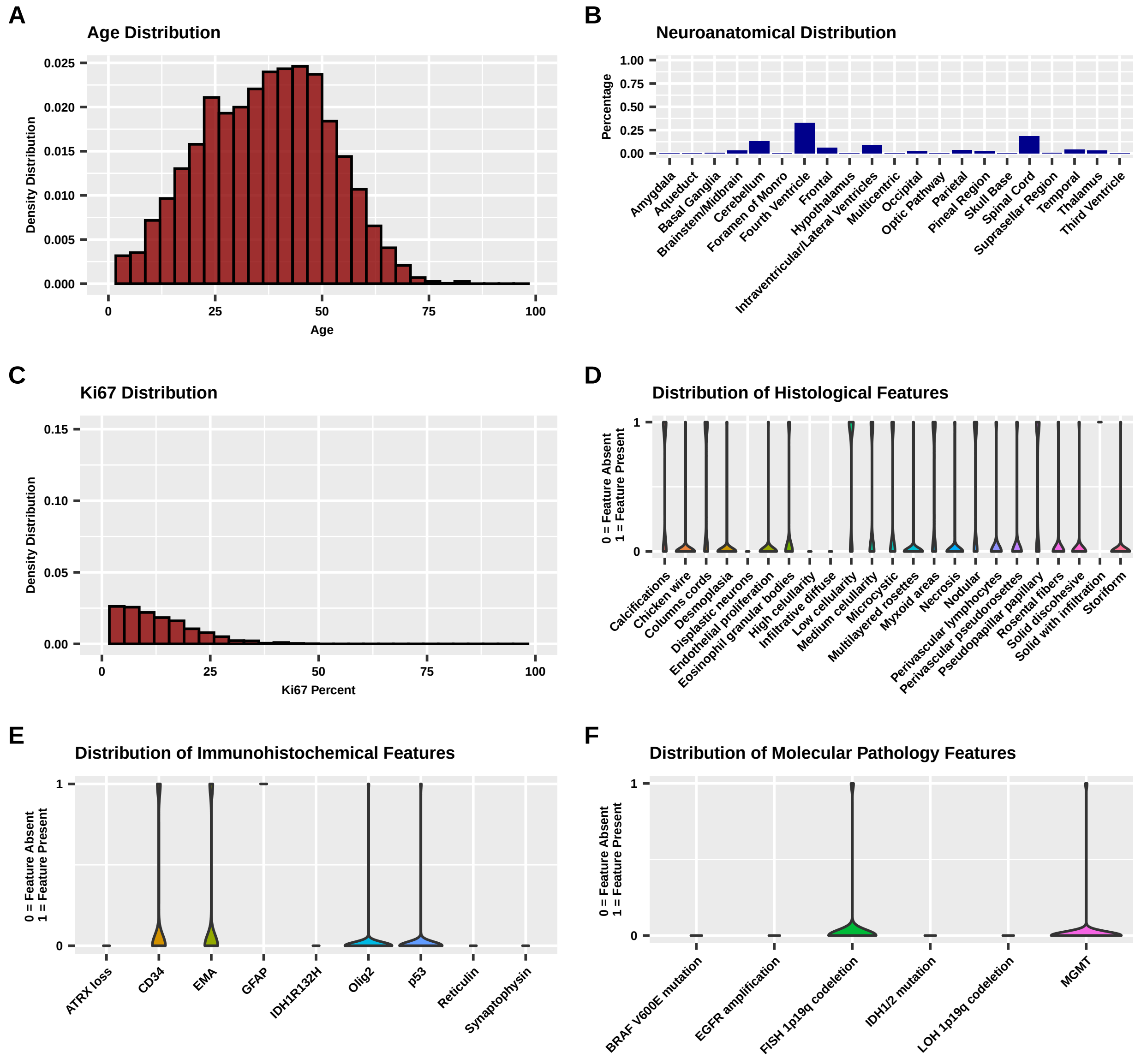

Figure S50. Subenpendymoma

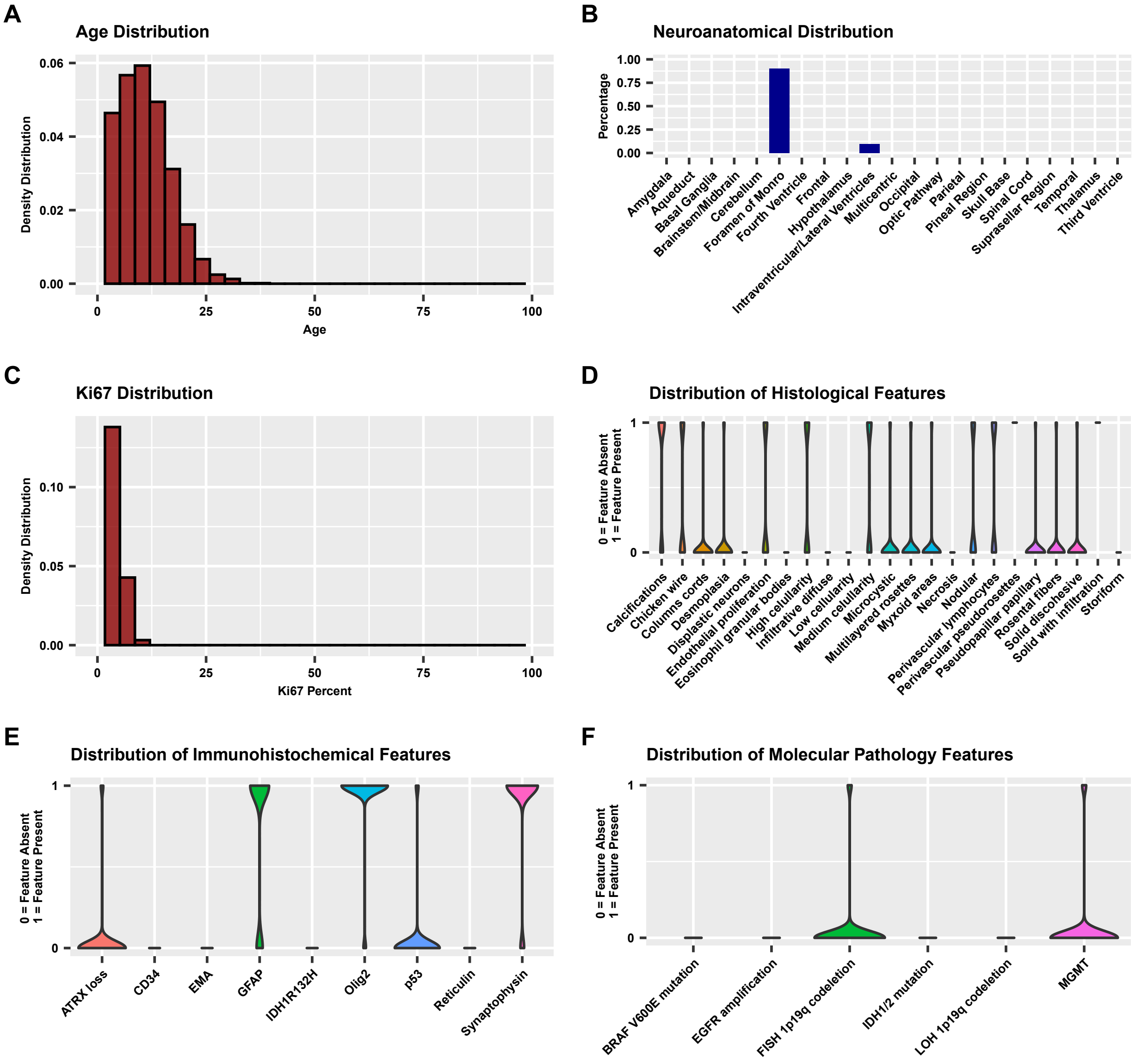

Figure S51. Supendymal Giant Cell Astrocytoma, WHO grade 1

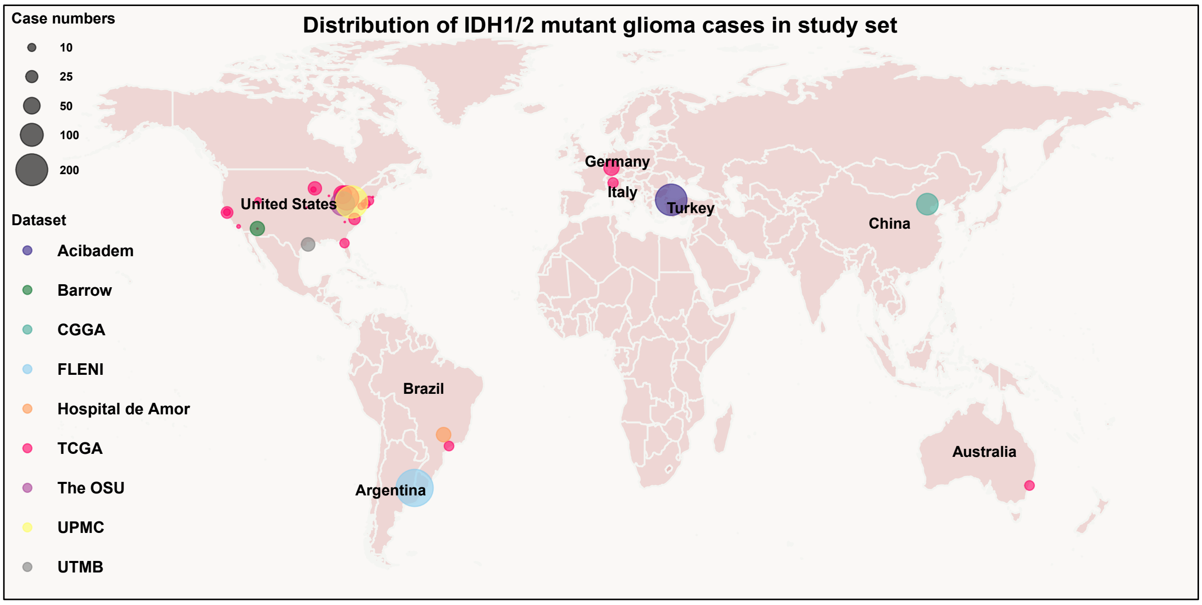

Figure S52. Distribution of cases in the global IDH mutant (Grade 2 and 3) dataset. (A) World map shows the distribution of the cases in the global dataset. TCGA study (purple circles) has cases from different areas of the world such as Italy, Germany, Australia, but mostly the United States. This overall distribution of global dataset shows a good variety of ethnicity and genetic background to represent a real-world data.

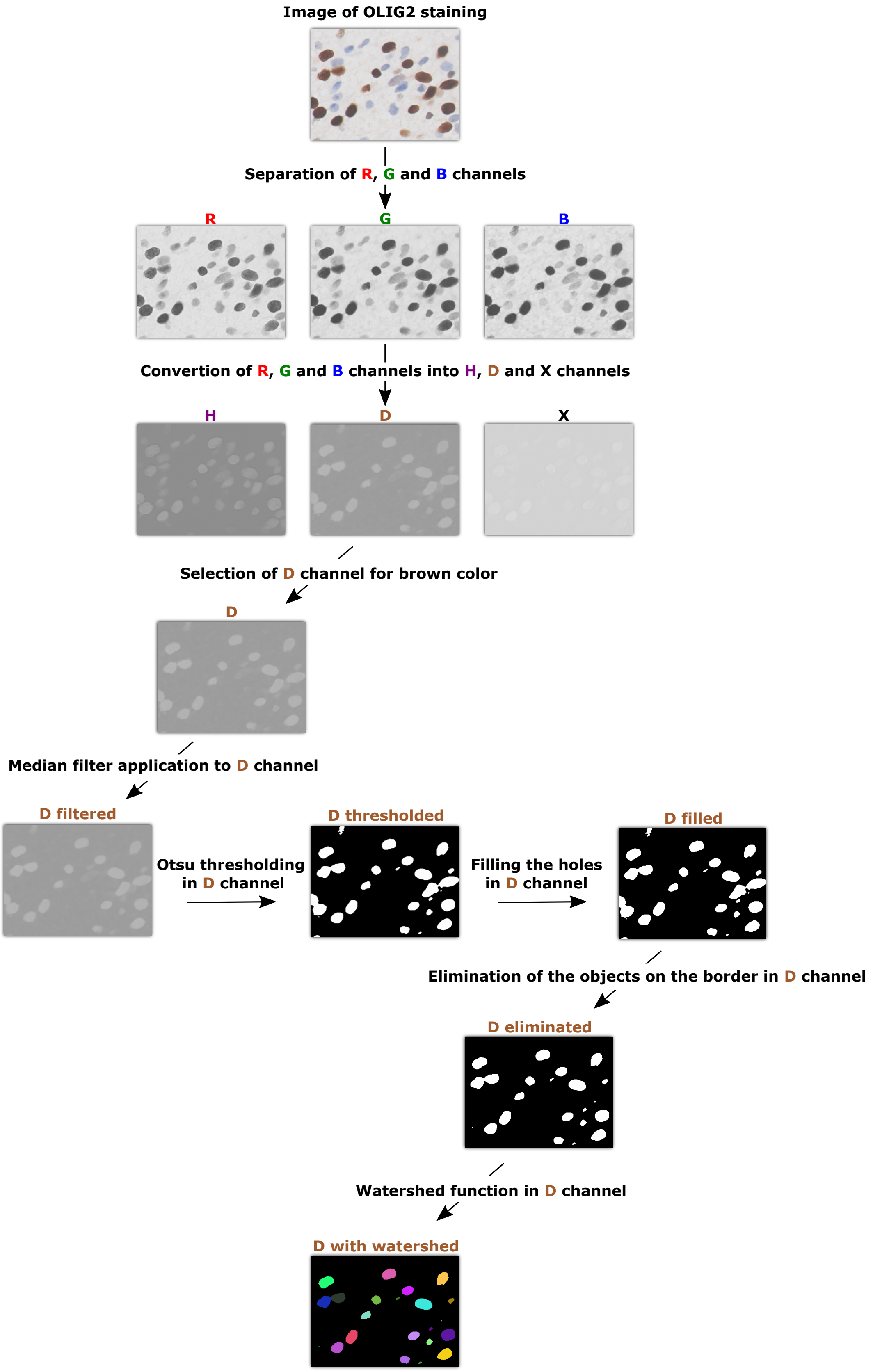

Fig. S53 Deconvolution with Otsu's thresholding and Watershed. The images were splitted into R, G and B channels. Then R (Red), G (Green) and B (Blue) channels were converted to H (Hematoxylin), D (DAB) and X channels. D channel was selected for brown color in OLIG2 staining. After application of median filtering, Otsu's thresholding was applied to the D channel. The holes in the nuclei were filled out and then the nu clei on the border were eliminated for a better representation of nuclei. As a last step, watershed function was applied for overlapping or contacting nuclei.

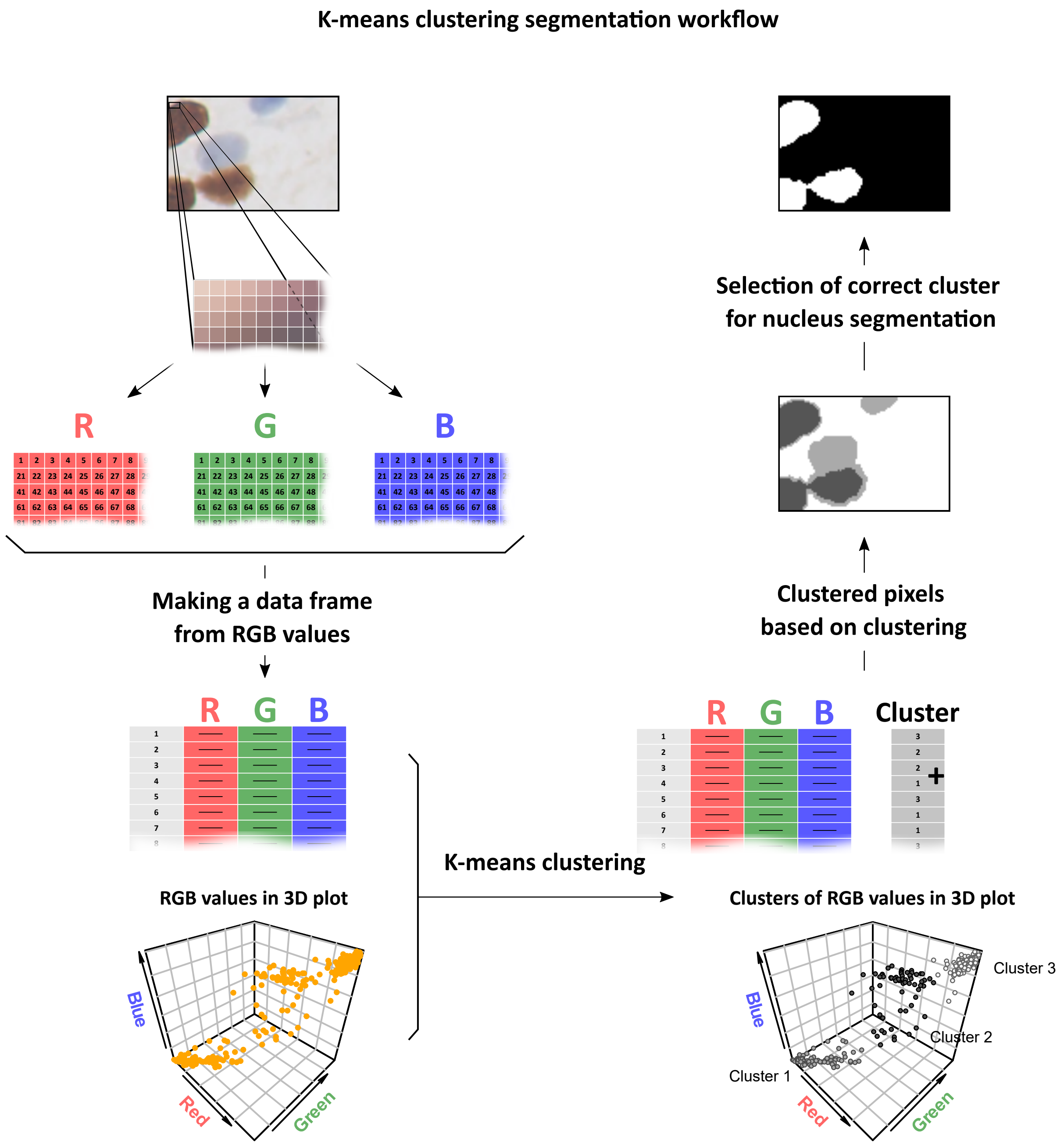

Fig. S54 K-means clustering segmentation. The images were converted to a data frame using R, G and B pixel intensity values. All pixels were clustered into 3 clusters using k-means clustering method. Each pixel was labeled as Cluster 1, Cluster 2, or Cluster 3. The cluster that contain specifically the nuclei was chosen as foreground and the other 2 clusters were accepted as background.

Sample Workflow for Cut-Cluster-Classify segmentation

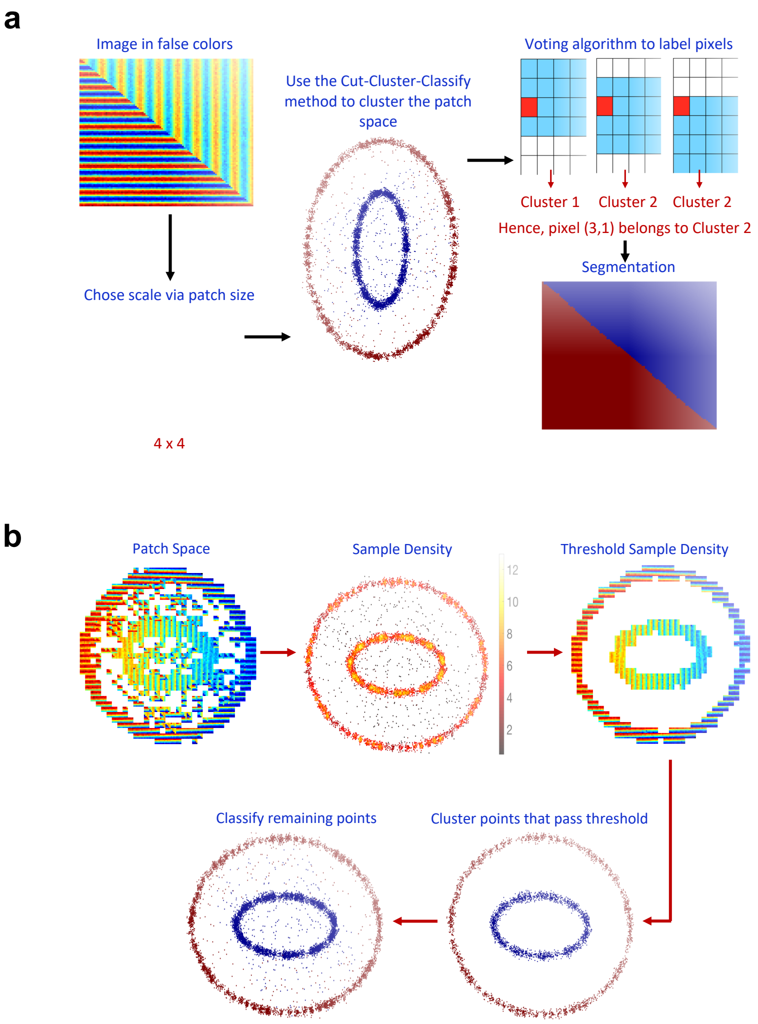

Fig. S55 Cut-Cluster-Classify (CCC) segmentation method. a All patches in the image are viewed as high dimensional points and a threshold is applied to the sample density (Cut) to get clusters. After clustering (Cluster), the patches below the threshold were classified based on the clusters (Classify). **b** Then all pixels were voted for clusters based on cluster result on overlapping patches. The cluster that contains nuclei was accepted as foreground and the other one was accepted as background. 5x5 patches were used for the segmentation.

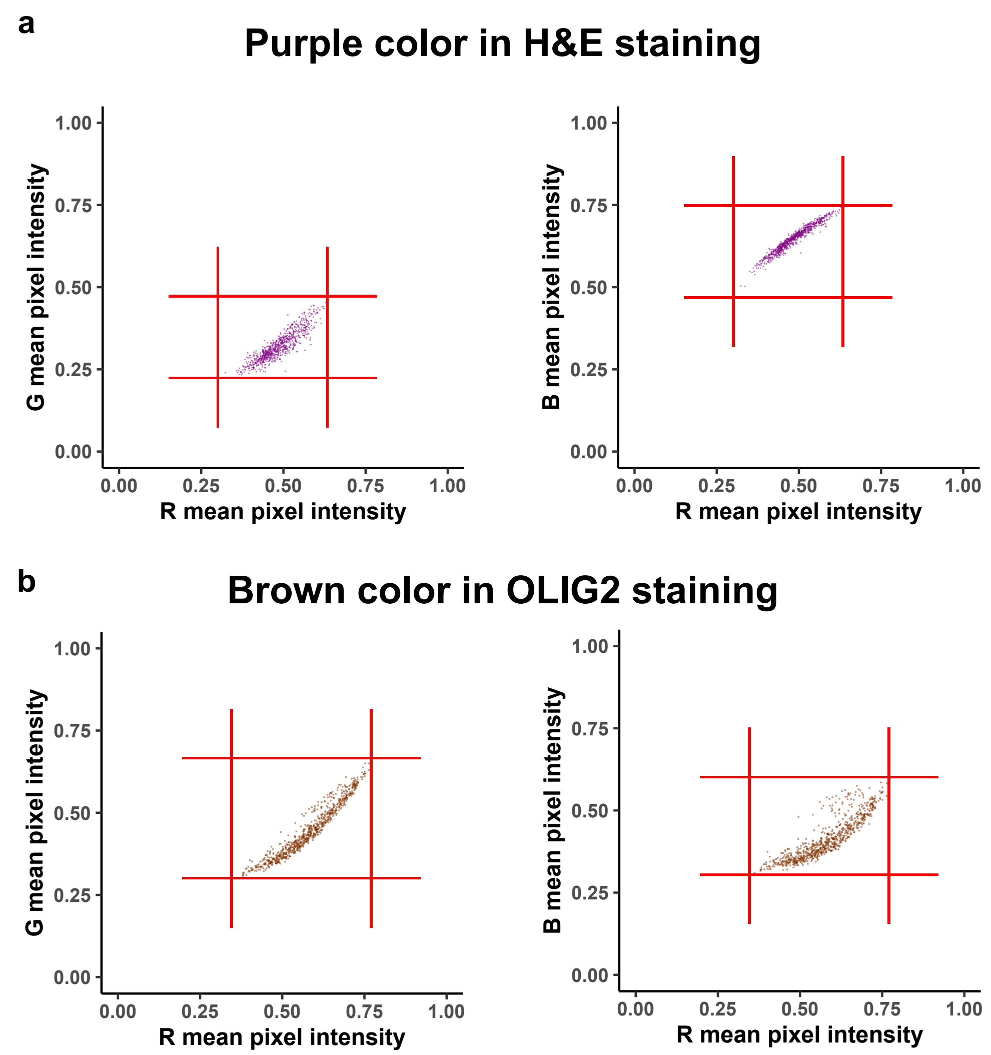

Fig. S56 Relationships of R channel pixel intensity values with G and B pixel intensity values for color of noise. Mean pixel intensity values of R channel are correlated to mean pixel intensity values of G and B channels. To better represent these spectrums, we used support vector machine (SVM) modeling to predict G and B values from R. The colors of noise are purple for H&E staining **(a)** and brown for OLIG2 staining **(b)**

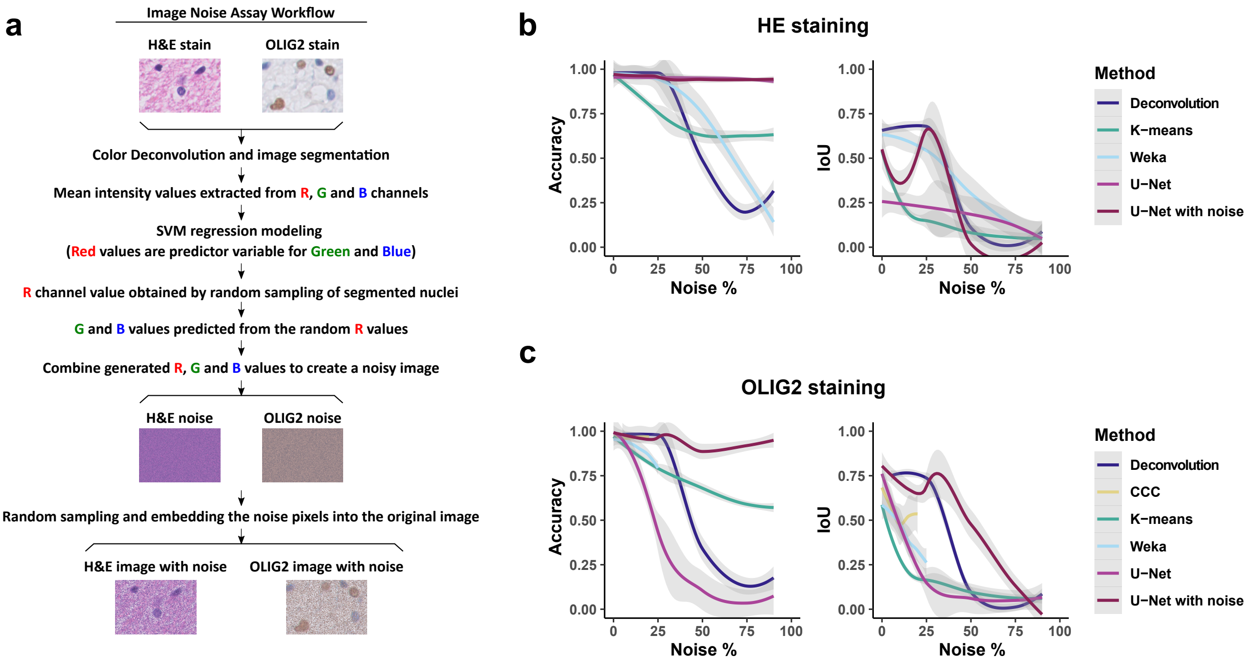

Fig. S57 Image noise assay workflow and comparison of supervised and unsupervised image segmentation methods for H&E and OLIG2 immunostaining. a An image noise assay was created for the fidelity evaluation of image segmentation methods to determine the best one for our purpose. We extracted the purple color for H&E staining and brown color for OLIG2 staining. A support vector model (SVM) was made to generate a noise similar to colors of the image. After generation of noise for a full image, the pixels were randomly sampled with certain percentages to implement the noise to the images. This workflow provided us to compare the performance of segmentation methods with different amount of noise. **b/c** We plotted segmentation performance by accuracy and intersection-over-union (IoU). Additionally, we added the U-Net trained with random noise generation as "U-Net with noise" to the comparison. In U-Net with noise, we trained a new model every time when we added noise to the image **b** In H&E staining, U-Net models show highest accuracies independent from the noise percentage. On the other hand, U-Net method shows low IoU, when U-Net with noise has a better IoU values. Overall deconvolution method has the U-Net with noise methods have the highest performance regarding accuracy and IoU. **c** In OLIG2 staining, similar to the H&E, deconvolution and U-Net with noise methods have the highest performance regarding accuracy and IoU. Despite deconvolution method has a sharp decrease after 25% noise, U-Net with noise maintained high IoU even after 25% noise.

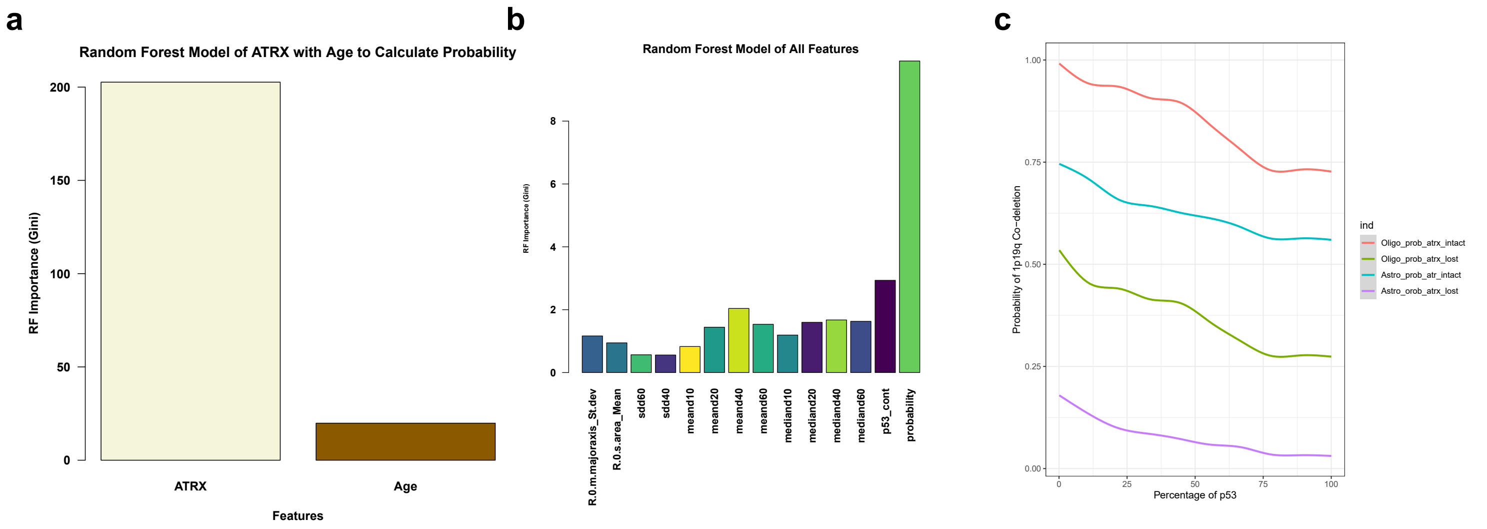

Fig. S58 . **a** Gini importance of RF model for the probability of 1p19q codeletion using ATRX and age. **b** Gini importance of final RF model used for the 1p19q codeletion in validation data set. **c** Simulation of the model with different p53 percentages showing 1p19q codeletion probability in different scenarios.
