# Supplementary material for: Information theory approaches to improve glioma diagnostic workflows in surgical neuropathology
Source: Brain Pathol. 2022 Jan 10;32(5):e13050. doi: 10.1111/bpa.13050 (PMC9425010; doi:10.1111/bpa.13050)
Supplement: Supplementary file 3 — Supplementary Material TABLE S2 Validation set data [file BPA-32-e13050-s002.docx]

**Information theory approaches to improve glioma diagnostic workflows in surgical neuropathology**

Lokman Cevik^1#^, Marilyn Vazquez Landrove^2#^, Mehmet Tahir Aslan^1^, Vasilii Khammad^3^, Francisco Jose Garagorry Guerra^4^, Yolanda Cabello-Izquierdo^1^, Wesley Wang^1^, Jing Zhao^5^, Aline Paixao Becker^1^, Catherine Czeisler^1^, Anne Costa Rendeiro^6^, Lucas Luis Sousa Véras^6^, Maicon Fernando Zanon^7^, Rui Manuel Reis^7,8^, Marcus de Medeiros Matsushita^9^, Koray Ozduman^10^, M. Necmettin Pamir^10^, Ayca Ersen Danyeli^11^, Thomas Pearce^12^, Michelle Felicella^13^, Jennifer Eschbacher^14^, Naomi Arakaki^15^, Horacio Martinetto^15^, Anil Parwani^1^, Diana L. Thomas^1^*, José Javier Otero^1^*

#Equal contribution

*Co-corresponding authors.

^1^ Department of Pathology, The Ohio State University Wexner Medical Center, Columbus, OH, USA

^2^ Mathematical Biosciences Institute, The Ohio State University, Columbus, OH, USA

^3^ Peoples' Friendship University of Russia, Moscow, Russia

^4^ Universidad de la República, Facultad de Medicina, UdeLaR, Cátedra de Anatomía Patológica, Hospital de Clínicas Manuel Quintela, Uruguay

^5^ Department of Biomedical Informatics, The Ohio State University College of Medicine, Columbus, OH, USA

^6^ Pathology Department, Barretos Cancer Hospital, Barretos, São Paulo, Brazil

^7^ Molecular Oncology Research Center, Barretos Cancer Hospital, Barretos, Brazil

^8^ Life and Health Sciences Research Institute (ICVS), School of Medicine, University of Minho, Braga, Portugal

^9^ MultiPat Laboratório de Anatomia Patológica, Campinas, São Paulo, Brazil

^10^ Department of Neurosurgery, Acibadem MAA University, Istanbul, Turkey

^11^ Department of Pathology, Acıbadem University School of Medicine, Istanbul, Turkey

^12^ Division of Neuropathology, Department of Pathology, University of Pittsburgh Medical Center, Pittsburgh, PA, USA

^13^ Division of Neuropathology, Department of Pathology, University of Texas Medical Branch, Galveston, Texas, USA

^14^ Department of Pathology, Barrow Neurological Institute - St. Joseph's Hospital and Medical Center, Phoenix, AZ, USA

^15^ Departamento de Neuropatología y Biología Molecular, Instituto de Investigaciones Neurológicas Dr Raúl Carrea (FLENI), Buenos Aires, Argentina

**Supplemental Materials and Methods**

**Glioma simulation model:** The purpose of this simulation was to determine which features contained the most information in rendering diagnoses. In order to organize the different tumor types, the most updated version of the WHO Classification of Tumors of the Central Nervous System in 2016 was taken to set up a Microsoft Excel table coined affectionately by Dr. Garragorry as “La Tabla”. Global cellularity is subdivided between low cellularity, medium cellularity, and high cellularity as per our experience. Pattern of growth is split into infiltrative diffuse and solid with infiltration. Architectural pattern is divided into nodular (nests), solid discohesive, cords or columns, chicken-wire, pseudopapillary, microcystic, myxoid areas, perivascular pseudorosettes, multilayered rosettes, storiform and desmoplasia. Type of cells according to frequency is subcategorized into astrocytic like (fibrillary, gemistocytic, piloid), resemble subependymal glia (including physal-cells), rounded cells with clear halo (including neurocytic cells), giant multinucleated cells, rhabdoid cells (cells with large eosinophilic cytoplasm, with rounded nuclei with prominent nucleoli), ganglion cells (large cells, with only one nuclei, usually euchromatic), lipidized cells (includes multivacuolated and focal accumulation of lipid-laden cells), small rounded blue cells and epitheloid cells. Low, medium, and high nuclear pleomorphism fall within the nuclear pleomorphism’s category. In some additional features dysplastic neurons (neurons that look like mature neurons but are binucleated), rosenthal fibers, eosinophil granular bodies, perivascular lymphocytes as well as calcifications are found. Referred to Ann-Mayo classification is divided into, mitosis, endothelial proliferation, and necrosis. Immunohistochemistry is subdivided between GFAP, ATRX loss, p53, IDH1 R123H, INI1 (loss stain) and Ki67. Finally, molecular data is divided into the presence or absence of IDH1 and IDH2 mutation, EGFR amplification, MGMT promoter methylation, BRAF V600E mutation, 1p/19q codeletion FISH and 1p/19q codeletion LOH. The majority of the information was found within the 2007 and 2016 version of the [1-4] WHO Classification of Tumors of the Central Nervous System and the CBTRUS [5, 6] and the references therein. References used for age included: [5-23]. Additional references for gender included: [5, 6, 24-29]. Additional references for neuroanatomical site included: [20, 30-49]. Additional references for immunohistochemical and molecular features included: [4, 25, 50-82]. The data structure of La Tabla included neuroanatomical site coded as a factor, age and ki67 coded as continuous numbers, and for all other features they were coded as dummy variables with 0 = negative and 1 = positive. Sex was coded as 0 = male and 1 = female. La Tabla is uploaded as a .R list object into a public repository (http://doi.org/10.6084/m9.figshare.16493964). For each tumor type modelled (51 tumor types in total), we generated 5 different plots. (A) shows a histogram of the modelled age, (B) shows the percentage of tumors in specific neuroanatomical sites, (C) histogram of the modelled ki67 labelling index, (D) violin plot of the distribution of the histological features, (E) violin plot of the immunohistochemical features of ATRX, GFAP, IDH1R132H, and P53. (F) Distribution of the molecular features BRAFV600E, EGFR amplification, 1p/19q co-deletion by FISH, 1p/19q co-deletion by chromosomal microarray, and MGMT promoter methylation status.

**Real world datasets for information theory validation:** The numbers of available cases for the calculations in nine different datasets were summarized in **Supplemental Table 2** including publicly available The Cancer Genome Atlas (TCGA) and Chinese Glioma Genome Atlas (CGGA). Grade 2 and 3 gliomas are selected for the study. However, grade information of each diagnosis is not available for all data sets. In these datasets, only *IDH1/2*-mutant diffuse gliomas (WHO grades 2-3) were selected. The “TCGAbiolinks” package in R was used to pull the information from the TCGA database. CGGA Part A was downloaded from the Chinese Glioma Genome Atlas website and used for the analysis. The distribution of cases in a world map was made using “maps”, “mapdata”, “ggthemes” and “ggplot2” packages in R. The centers in the TCGA were shown separately to demonstrate the actual genetic background and diversity in the whole dataset. The latitude and longitude of centers for the world map were obtained from Google maps.

**Information theory calculations:** The “infotheo” package in R was used for all mutual information and entropy calculations. Permutation test was used for the statistical significance of the mutual information and the differences between the mutual information. For permutation test, the features were scrambled, and calculations were repeated 10,000 times. Then, p values were calculated based on the probability of the results by chance as described previously [83].

**Image acquisition:** The images were extracted from the whole slide imaging archives of The Ohio State University. Glass slides were scanned by a Philips IntelliSite UltraFast Scanner (Philips Digital Pathology Solutions, Best, the Netherlands) with a resolution of 0.25 microns/pixel at 40x magnification. 5-8 randomly selected image patches were exported for each case via Philips IMS software. Image patches have a dimension of 2142 x 1277 on average for H&E staining, 1987 x 1170 on average for Olig2 staining, and 2214 x 1570. on average for p53 staining. Mean segmented nuclei numbers are 4101 for H&E images, 1977 for Olig2 images, and 3774 for p53 images per case.

**Comparison of image segmentation methods (unsupervised and supervised):** We used different types of segmentation methods: unsupervised (Deconvolution combined with Otsu thresholding, K-means, and the Cut-Cluster-Classify), and supervised methods (Trainable Weka Segmentation and U-Net). With the exception of the Cut-Cluster-Classify method, all methods have been previously used in a similar context of segmenting cell nuclei from medical images with good results [84-86]. Unless otherwise stated, all methods take the raw RGB color intensity information to segment the image. An explanation of how we evaluated fidelity is included at the end of this section.

*Unsupervised Methods:*

*Deconvolution with Otsu’s thresholding and Watershed:* The Otsu method developed by Ruifrok and Johnston [87] was combined with the watershed for segmentation as described by Yi et al. [84] Otsu’s thresholding is a method that automatically chooses a color intensity value that separates the foreground from the background by analyzing the color histogram of the image [88]. After the thresholding is applied, a Watershed function is used to separate overlapping cells.

In our analysis, colors of stains were separated using scikit-image via reticulate in RStudio. RGB channels of the images were converted to HED channels (Hematoxylin + Eosin + DAB) in H&E-stained images and HDX channels (Hematoxylin + DAB) in Olig2 stained images (Workflow for Olig2 stain in **Supplementary Fig. 53**). The channels that the nuclei present were selected for the segmentation. Median filter and Otsu thresholding of the EBImage package were applied to the images and then spaces in the nuclei were filled. For feature extraction, in addition to this process, nuclei positioned on the image borders were eliminated and watershed function was used for the separation of overlapped or contacting nuclei.

*K-means clustering:* For this project, pixel intensity values of the RGB channels were converted to a 3-dimensional data for k-means clustering (**Suplementary Fig. 54**) The number of clusters was selected as 3 for k-means clustering [89]. The cluster that includes nuclei was selected for the foreground and the other 2 clusters were accepted as the background to generate binary masks for the comparison.

*Cut-Cluster-Classify (CCC):* CCC is a density-based clustering method that starts by thresholding the sample density of points, clustering the points that pass the threshold, and finishes the labelling via the use of a classifier (**Supplementary Fig. 55b**) [90]. For image segmentation, this method is applied to all the image patches, or squared windows of a fixed size inside the image, instead of the pixels directly, and then a maximum voting algorithm is calculated to go from patch labels to pixel labels (**Supplementary Fig. 55a**). Using patches instead of pixels allows for local patterns, such as neighborhoods of pixels, to be analyzed and taken into account [90]. We applied the Cut-Cluster-Classify to find 2 regions in the RGB images using 5 by 5 patches and used Watershed to separate overlapping nuclei.

*Supervised Methods*

*Trainable Weka segmentation:* Trainable Weka segmentation is an open-source software developed to handle image segmentation of biological images using machine learning solutions [91]. To produce pixel-based segmentation, we used a Fiji plugin to apply the Trainable Weka segmentation. Ground truths were made in 10 images correcting the classifier after every training for each image. Default parameters were used for training of classifiers. After creating segmentation plots, these plots were converted to binary masks in Fiji.

*U-NET:* Details for U-Net can be found in [92]. One of the drawbacks of neural nets is its need for large amounts of training data, or labeled images, in order to be accurate. U-Net makes use of data augmentation, which is a method in which original images and their ground truths are passed through various transformations and added as new training images.

Image patches were subdivided into 512x512 image tiles to ensure consistent network training, generating 120 tiles. Ground truth image annotations were performed using GIMP as mentioned below. In our network training schemes, 2 training libraries were used to develop 2 separate U-Net neural networks-- one trained without noised images and one with noised images. Image noising was performed as described in the image noise assay below. Between both models however, the network architecture and workflow to generate models and predicted masks were the same. Network architecture was written in python using tensorflow [93]. For training, 110 image tiles were used, and the remaining 10 images were used to test our models. Images underwent 5 layers of convolution in the contraction pathway to generate 16, 32, 64, 128, and 256 features maps at each layer. Four layers of convolution were utilized in the expansive pathways to generate 16, 32, 64 and 128 feature maps which were transposed and concatenated with respective feature maps in the contraction path. Image padding was used to preserve borders and a 3x3 kernel matrix was used during convolution with a reLU activation function [94]. During training, a binary cross entropy loss function was used for optimization [95]. Finally, upon mask prediction, predicted masks were converted to binary and exported for use in R to assess model performance.

*Measures of Fidelity:* To define the accuracy, let P denote the total number of nuclei pixels, N denote the total number of background pixels, TP denote the total number of correctly labeled nuclei pixels, and TN denote the total number of correctly labeled background pixels. Then accuracy is simply the fraction of correctly labeled points $\left( \frac{TP + TN}{P + N} \right)$. To define IoU, let A denote the ground truth area of pixels from a nucleus and B denote the segmentation results for that same nucleus. The IoU is simply the area of overlap divided by the area of union $\left( \frac{A\cap B}{A\cup B} \right)$.

To evaluate segmentation fidelity, we used a total of 10 testing images (512 by 512) for each staining in all supervised and unsupervised methods. The Deconvolution with Otsu and the K-means were applied using RStudio software, CCC and U-Net were applied using Python, and Weka was applied using FIJI. These results were run in a Ryzen 7 laptop with integrated graphics and 16GB of RAM. One of the challenges of working with stained tissues is the varying noise level that comes from the staining techniques. Therefore, we also compared the methods against increasing levels of simulated noise to have better control over the ground truth masks.

*Resistance to noise: Image noise assay:* An image noise assay was developed to compare the resistance of segmentation methods to noise. In order to create a relevant, noisy image, the mean intensity values of the nuclei were taken, and then a support vector machine modeling was made to predict G and B values from the R values (non-linear relationships of R, G and B colors in H&E and Olig2 images are shown in **Supplementary Fig. 56**). These models were made for each image because the noise would be different for each image and this approach represents a similar color of nuclei in the noise. The same size image with added noise was created via the prediction of G and B values from the R values of the image and then random pixels were selected as percentages to implement different noise ratios into the original images. Percentage ratios of added noise were selected as 0, 5, 10 15, 25, 50, 75, 90 to evaluate the whole pattern of noise resistance.

*Exclusion of incomplete nuclei:* In image patches, there were incomplete nuclei due to the 3-dimensional position of nuclei in the paraffin blocks during dissection. For a better representation of nuclear features, a ground truth was made for the prediction of incomplete and complete nuclei. 1000 incomplete nuclei (500 for astrocytoma and 500 for oligodendroglioma) and 1000 complete nuclei (500 for astrocytoma and 500 for oligodendroglioma) were labeled for ground truth. A total of 2000 nuclei were split into training (70%) and testing (30%) for modeling. A random forest modeling was made with a 97.83 accuracy to predict the incomplete nuclei. This model was used a filter prior to downstream applications.

*Making Ground truth with GIMP and R:* GIMP is a free open-source image manipulation software. GIMP software and R codes were used to make ground truth masks. GIMP was used for freehand annotation of nuclei in a similar approach in [96]. Then, the annotated layer was exported as a PNG file. This PNG file was imported to RStudio for further manipulation. The layer was converted to grayscale and applied Otsu thresholding. After thresholding, the fillHull function of EBImage was used to fill the space in the nuclei of binary masks. These ground truth masks were used for the evaluation of segmentation fidelity and the training of U-Net.

**Case histories from patients utilized in the validation study:** In order to validate our workflows, we identified eleven cases from ten patients that were particularly challenging diagnostically. Some of these cases had been evaluated prior to widespread implementation of cIMPACT guidelines. These cases refer to the cases utilized in the validation study in **Figure 6** of the main text.

- **Case 1:** A 57-year-old female with a history of oligodendroglioma, WHO grade 2 diagnosed over ten years prior to the current surgery. The patient suffered refractory seizures, and an imaging study performed one month prior to surgery showed a non-enhancing, FLAIR hyperintense lesion that had increased in size relative to prior imaging. The patient underwent surgical resection of the lesion. Histological evaluation demonstrated a cytologically low grade infiltrating astrocytoma diffusely positive for OLIG2 and IDH1R132H. ATRX expression was lost in tumor cell nuclei, and p53 showed intense immunoreactivity. A 1p/19q FISH analysis returned co-deletion positive, with a 1p/1q ratio of 0.64 and a 19q/19p ratio of 0.59. EGFR was negative for amplification by FISH, and MGMT analysis showed promoter hypermethylation. This case was diagnosed as astrocytoma, IDH-mutant, WHO grade 2. The 1p/19q co-deletion was deemed a false positive result, and no further molecular evaluation was performed as recommended by cIMPACT guidelines.
- **Case 2:** A 38-year-old female with a prior history of left frontal oligodendroglioma diagnosed at an outside hospital who on routine follow-up imaging showed development of a nodular, enhancing region in the left frontal area that was concerning for recurrence. She underwent resection, and the histopathology demonstrated diffusely infiltrating glioma with mitoses and microvascular hyperplasia without necrosis. IDH1R132H immunohistochemistry was positive, ATRX expression was retained in tumor cell nuclei, and p53 immunoreactivity was reported positive. Molecular studies revealed a positive 1p/19q co-deletion by FISH, MGMT hypermethylation, and negative amplification of EGFR. Chromosomal microarray (CMA) analysis was performed using molecular  inversion probes on a whole genome array (Affymetrix OncoScan platform). These studies showed Genomic alterations include loss of 4q31.22q35.2, gain of 7p12.1q36.3, gain of 8q22.1q24.3, complex loss of heterozygosity (LOH) with multiple focal losses on 9p (including homozygous loss of CDKN2A and CDKN2B), gain of 10p15.3p11.1, loss of 11p15.5p15.2, loss of 12q23.1q24.31, multiple losses on 14q, copy neutral LOH (cnLOH) of 17p13.311.1 (including TP53) . A final diagnosis of astrocytoma, IDH-mutant, WHO grade 2 was rendered. The 1p/19q co-deletion by FISH was deemed a false positive result.
- **Case 3-1 and 3-2:** These two surgical neuropathology cases represent two specimens resected from the same patient four years apart. Case 3-1 represents patient’s second neuro-oncological presentation, with case 3-2 representing a third recurrence with resection of the same. Case 3-1 presented four years following the patient’s first recurrence, and case 3-2 thus occurring 8 years after the initial neuro-oncological evaluation. Case 3-1 was characterized by diffusely infiltrating neoplastic oligodendroglioma cells without high grade architectural features (specifically, low mitotic count and an absence of both microvascular hyperplasia and necrosis). IDH1R132H was immunoreactive, ATRX expression was retained in tumor cell nuclei, and p53 negative. Molecular evaluation demonstrated a 1p/19q co-deletion (1p/1q ratio of 0.49 and a 19q/19p ratio of 0.51). The specimen was MGMT unmethylated. Chromosomal microarray demonstrated a genomic profile characterized by co-deletion of the whole arms of 1p and 19q. Additional alterations included loss of 14q23.1q31.3. Thus, the 1p/19q co-deletion of case 3-1 is deemed to represent a true positive result, and a diagnosis of oligodendroglioma, WHO grade 2 was rendered. Case 3-2 was characterized by high grade features including mitoses and microvascular hyperplasia, as well as an overwhelmingly astrocytic tumor morphology. IDh1R132H was positive, with ATRX expression was retained in tumor cell nuclei and negative p53 staining. Molecular evaluation revealed an unmethylated MGMT promoter, negative detection of EGFR amplification, and a 1p/19q co-deletion. Chromosomal microarray revealed multiple genomic alteration include copy neutral loss of heterozygosity (cnLOH) of the 1p arm with multiple focal deletions, gain of 1q21.1q31.1, gain of 2q22.1 (disrupting LRP1B), gain of chromosome 4 with focal deletion of 4q24q25 (including TET2), cnLOH of 9p24.3p13.3 with focal homozygous loss of 9p21.3 (including CDKN2A and CDKN2B), loss of 10q24.2q25.1, loss of 14q23.1q31.3, gain of the 19p arm and cnLOH of the 19q arm. However, whole arm loss of chrosomomes 1p and 19q was absent. A diagnosis of astrocytoma, IDH-mutant, WHO grade 2 was rendered. The 1p/19q co-deletion by FISH was deemed a false positive in case 3-2.
- **Case 4:** The patient is a 63-year-old man with history of prostate cancer who presented with altered mental status, with a left frontal mass found on CT during evaluation in the emergency department. The patient underwent surgical resection, which demonstrated a diffusely infiltrating glioma with mitoses, microvascular proliferation, and necrosis. The tumor cells were IDH1R132H immunoreactive, with an equivocal ATRX result (negative staining in endothelial cells and tumor cells was noted and thus was not possible to ascertain by immunohistochemistry). P53 showed intense immunoreactivity. MGMT promoter methylation was reported as having just crossed the threshold to positivity in our assay. EGFR was not amplified, and 1p19q co-deletion was sufficient for positive designation (ratios of 1p/1q and 19q/19p were both 0.75). Chromosomal microarray revealed a pattern consistent with an IDH-mutant astrocytoma without CDKN2A/B homozygous deletion and without loss of 1p and 19q. A diagnosis of astrocytoma, IDH-mutant, WHO grade 2 was rendered, and the 1p/19q FISH was deemed a false positive result.
- **Case 5:** A 35-year-old man with a right frontal lobe mass underwent resection. The tissue demonstrated both round and elongate nuclear morphology on the H&E stained section. IDH1R132H was detected by immunohistochemistry, ATRX expression was lost in tumor cell nuclei, and diffuse p53 expression. The specimen showed MGMT hypermethylation, negative BRAFV600E status, negative EGFR amplification, and 1p/19q co-deletion by FISH (1p/1q ratio of 0.71 and 19q/19p ratio of 0.78). This case was evluated prior to cIMPACT guidelines were disseminated and thus diagnosed as an anaplastic oligodendroglioma, WHO grade 3. The patient experienced a recurrence 4 years later of a astrocytoma, WHO grade 4, and an NGS panel was performed at tempus of the original case which did not conform the 1p/19q co-deletion. This, the 1p/19q FISH was deemed a false positive.
- **Case 6:** The patient is a 37-year-old female with a temporal lesion. Her symptoms had started with seizures, first reported 17 years prior to the patient’s resection. Upon evaluation for long-term seizure management, an MRI performed 3 years prior to the resection had identified a non-enhancing tumor. Histological findings showed diffusely infiltrating oligodendroglioma cells showing IDH1R132H expression, ATRX expression was retained in tumor cell nuclei, and negative p53 status. EGFR amplification was negative, and a 1p/19q co-deletion was detected by FISH (1p/1q and 19q/19p ratios were both 0.44). No further evaluation was performed.
- **Case 7:** The patient is a 26-year-old female who suffered a motor vehicle accident likely due to a new-onset seizure. Evaluation revealed a right temporal brain tumor, and the patient underwent neurosurgical resection. The tissue sections demonstrated diffusely infiltrating neoplastic astrocytes with mitoses but without microvascular hyperplasia and no necrosis. IDH1R132H was positive, ATRX expression was lost in tumor cell nuclei, and p53 showed strong nuclear intensity in the tumor cells. 1p/19q co-deletion was not tested by FISH. A chromosomal microarray (performed to evaluate CDKN2a/b) did not show any 1p or 19q whole arm deletion. The EGFR was not amplified, and MGMT promoter methylation was detected.
- **Case 8:** The patient is a 31-year-old male with a history of depression, opioid substance abuse, and depression/anxiety disorder who presented with new onset seizures. CT imaging demonstrated a right frontal lobe mass. Further evaluation revealed a T2/FLAIR hyperintense lesion without enhancement that underwent resection. Evaluation of the histology demonstrated a diffusely infiltrating glioma without microvascular hyperplasia and without necrosis. IDH1R132H was positive, ATRX expression was retained in tumor cell nuclei, and p53 showed no immunoreactivity in the tumor cells. A 1p/19q co-deletion by FISH was positive (1p/1q ratio was 0.35, and 19q/19p ratio was .044). MGMT promoter hypermethylation was detected, and EGFR showed no amplification. A diagnosis of oligodendroglioma, WHO grade 2 was rendered and no further molecular evaluation was performed.
- **Case 9:** The patient is a 44-year-old male with a history of multiple craniotomies for anaplastic oligodendroglioma. The patient underwent surveillance imaging which showed increased growth of an enhancing lesion and underwent a resection. The patient’s histopathology showed necrosis, mitoses, and microvascular hyperplasia. IDH1R132H was positive, ATRX was retained in tumor cell nuclei, and p53 was negative. Molecular evaluation showed MGMT promoter hypermethylation, no EGFR amplification, and 1p/19q co-deletion (1p/1q ratio was 0.362, 19q/19p ratio was 0.51). Chromosomal microarray was performed, which confirmed whole arm loss of arms 1p and 19q. A diagnosis of anaplastic oligodendroglioma, WHO grade 3 was rendered, and FISH result was deemed to represent a true positive.
- **Case 10:** The patient is a 33-year-old male with a prior history of high grade brain cancers diagnosed at an outside hospital. The patient showed increased growth of a contrast enhancing lesion in their surveillance imaging and underwent resection. The histopathology was notable for diffusely infiltrating neoplastic astrocytes, necrosis, and microvascular hyperplasia. The patient showed IDH1R132H immunoreactivity, ATRX expression was lost in tumor cell nuclei, and negative labelling for p53. EGFR was negative for amplification, and MGMT promoter hypermethylation was noted (1p/19q co-deletion testing by FISH was not performed). Chromosomal microarray was performed to establish CDKN2A/B status and showed no evidence of codeletion of arms 1p and 19q.

**References for Supplementary Information**

1. Lyle, M.R., et al., *Newly Identified Characteristics and Suggestions for Diagnosis and Treatment of Diffuse Leptomeningeal Glioneuronal/Neuroepithelial Tumors: A Case Report and Review of the Literature.* Child Neurol Open, 2015. **2**(1): p. 2329048X14567531.

2. Rodriguez, F.J., et al., *Disseminated oligodendroglial-like leptomeningeal tumor of childhood: a distinctive clinicopathologic entity.* Acta Neuropathol, 2012. **124**(5): p. 627-41.

3. Ohgaki, H., et al., *Mutations of the p53 tumor suppressor gene in neoplasms of the human nervous system.* Mol Carcinog, 1993. **8**(2): p. 74-80.

4. Min, H.S., et al., *Medulloblastoma: histopathologic and molecular markers of anaplasia and biologic behavior.* Acta Neuropathol, 2006. **112**(1): p. 13-20.

5. Louis, D.N., Wiestler, O., Cavenee, W., Ellison, D., Figarella-Branger, D., Perry, A., Reifenberger, G., Deimling, A., *WHO Classification of Tumors of the Central Nervous System*, ed. W.H. Organization. 2016, Geneva, Switzerland.

6. CBTRUS, *Statistical Report: Primary Brain Tumors in the United States, 2000–2004. Published by Central Brain Tumor Registry of the United States*. 2008: .

7. Dunham, C., *Pediatric brain tumors: a histologic and genetic update on commonly encountered entities.* Semin Diagn Pathol, 2010. **27**(3): p. 147-59.

8. Rigau, V., et al., *French brain tumor database: 5-year histological results on 25 756 cases.* Brain Pathol, 2011. **21**(6): p. 633-44.

9. Matyja, E., et al., *Heterogeneity of histopathological presentation of pilocytic astrocytoma - diagnostic pitfalls. A review.* Folia Neuropathol, 2016. **54**(3): p. 197-211.

10. Nguyen, H.S., et al., *Subependymal Giant Cell Astrocytoma: A Surveillance, Epidemiology, and End Results Program-Based Analysis from 2004 to 2013.* World Neurosurg, 2018. **118**: p. e263-e268.

11. Perkins, S.M., et al., *Patterns of care and outcomes of patients with pleomorphic xanthoastrocytoma: a SEER analysis.* J Neurooncol, 2012. **110**(1): p. 99-104.

12. Alexandrescu, S., et al., *Epithelioid Glioblastomas and Anaplastic Epithelioid Pleomorphic Xanthoastrocytomas--Same Entity or First Cousins?* Brain Pathol, 2016. **26**(2): p. 215-23.

13. Jain, A., et al., *Subependymoma: clinical features and surgical outcomes.* Neurol Res, 2012. **34**(7): p. 677-84.

14. Barton, V.N., et al., *Unique molecular characteristics of pediatric myxopapillary ependymoma.* Brain Pathol, 2010. **20**(3): p. 560-70.

15. Hasselblatt, M., et al., *Immunohistochemical profile and chromosomal imbalances in papillary tumours of the pineal region.* Neuropathol Appl Neurobiol, 2006. **32**(3): p. 278-83.

16. Ahmed, K.A., et al., *Astroblastomas: a Surveillance, Epidemiology, and End Results (SEER)-based patterns of care analysis.* World Neurosurg, 2014. **82**(1-2): p. e291-7.

17. Romero-Rojas, A.E., et al., *Histopathological and immunohistochemical profile in anaplastic gangliogliomas.* Neurocirugia (Astur), 2013. **24**(6): p. 237-43.

18. VandenBerg, S.R., *Desmoplastic infantile ganglioglioma and desmoplastic cerebral astrocytoma of infancy.* Brain Pathol, 1993. **3**(3): p. 275-81.

19. Komori, T., et al., *Papillary glioneuronal tumor: a new variant of mixed neuronal-glial neoplasm.* Am J Surg Pathol, 1998. **22**(10): p. 1171-83.

20. Yang, C., et al., *Histopathological, molecular, clinical and radiological characterization of rosette-forming glioneuronal tumor in the central nervous system.* Oncotarget, 2017. **8**(65): p. 109175-109190.

21. Curran, E.K., et al., *Gender affects survival for medulloblastoma only in older children and adults: a study from the Surveillance Epidemiology and End Results Registry.* Pediatr Blood Cancer, 2009. **52**(1): p. 60-4.

22. Brown, H.G., et al., *"Large cell/anaplastic" medulloblastomas: a Pediatric Oncology Group Study.* J Neuropathol Exp Neurol, 2000. **59**(10): p. 857-65.

23. Okamatsu, C., et al., *Clinicopathological characteristics of ganglioneuroma and ganglioneuroblastoma: a report from the CCG and COG.* Pediatr Blood Cancer, 2009. **53**(4): p. 563-9.

24. Ahmadi, R., et al., *No prognostic value of IDH1 mutations in a series of 100 WHO grade II astrocytomas.* J Neurooncol, 2012. **109**(1): p. 15-22.

25. Kleinschmidt-DeMasters, B.K., et al., *Epithelioid GBMs show a high percentage of BRAF V600E mutation.* Am J Surg Pathol, 2013. **37**(5): p. 685-98.

26. Blakeley, J. and S. Grossman, *Anaplastic oligodendroglioma.* Curr Treat Options Neurol, 2008. **10**(4): p. 295-307.

27. Theeler, B.J., et al., *Adult pilocytic astrocytomas: clinical features and molecular analysis.* Neuro Oncol, 2014. **16**(6): p. 841-7.

28. Giorgianni, A., et al., *Lhermitte-Duclos disease. A case report.* Neuroradiol J, 2013. **26**(6): p. 655-60.

29. He, W.G., et al., *Clinical and biological features of neuroblastic tumors: A comparison of neuroblastoma and ganglioneuroblastoma.* Oncotarget, 2017. **8**(23): p. 37730-37739.

30. Larjavaara, S., et al., *Incidence of gliomas by anatomic location.* Neuro Oncol, 2007. **9**(3): p. 319-25.

31. Poulen, G., et al., *Huge heterogeneity in survival in a subset of adult patients with resected, wild-type isocitrate dehydrogenase status, WHO grade II astrocytomas.* J Neurosurg, 2018: p. 1-10.

32. Oslobanu, A., Florian, S.I., *Anatomic Locations in high grade gliomas.* Romanian Neurosurgery, 2015. **3**: p. 269-275.

33. Furuta, T., et al., *Clinicopathological and genetic association between epithelioid glioblastoma and pleomorphic xanthoastrocytoma.* Neuropathology, 2018. **38**(3): p. 218-227.

34. Solomon, D.A., et al., *Diffuse Midline Gliomas with Histone H3-K27M Mutation: A Series of 47 Cases Assessing the Spectrum of Morphologic Variation and Associated Genetic Alterations.* Brain Pathol, 2016. **26**(5): p. 569-80.

35. Smits, M., *Imaging of oligodendroglioma.* Br J Radiol, 2016. **89**(1060): p. 20150857.

36. Collins, V.P., D.T. Jones, and C. Giannini, *Pilocytic astrocytoma: pathology, molecular mechanisms and markers.* Acta Neuropathol, 2015. **129**(6): p. 775-88.

37. Kraetzig, T., et al., *Metastases of spinal myxopapillary ependymoma: unique characteristics and clinical management.* J Neurosurg Spine, 2018. **28**(2): p. 201-208.

38. Raghunathan, A., et al., *Histological predictors of outcome in ependymoma are dependent on anatomic site within the central nervous system.* Brain Pathol, 2013. **23**(5): p. 584-94.

39. Preusser, M., et al., *Angiocentric glioma: report of clinico-pathologic and genetic findings in 8 cases.* Am J Surg Pathol, 2007. **31**(11): p. 1709-18.

40. Lellouch-Tubiana, A., et al., *Angiocentric neuroepithelial tumor (ANET): a new epilepsy-related clinicopathological entity with distinctive MRI.* Brain Pathol, 2005. **15**(4): p. 281-6.

41. Sethi, D., et al., *Choroid plexus papilloma.* Asian J Neurosurg, 2017. **12**(1): p. 139-141.

42. Dudley, R.W., et al., *Pediatric low-grade ganglioglioma: epidemiology, treatments, and outcome analysis on 348 children from the surveillance, epidemiology, and end results database.* Neurosurgery, 2015. **76**(3): p. 313-9; discussion 319; quiz 319-20.

43. Cho, H.J., et al., *Primary diffuse leptomeningeal glioneuronal tumors.* Brain Tumor Pathol, 2015. **32**(1): p. 49-55.

44. Gaillard, F., *Central Neurocytoma: Radiology Reference Article.* Radiopaedia Blog RSS, 2016.

45. Mishra, T., N.A. Goel, and A.H. Goel, *Primary paraganglioma of the spine: A clinicopathological study of eight cases.* J Craniovertebr Junction Spine, 2014. **5**(1): p. 20-4.

46. Koeller, K., Rushing, E., *Medulloblastoma: A comprehensive review with radiologic-pathologic correlation.* Radiographics, from the archives of the AFIP.

47. Leonard, J.R., et al., *Large cell/anaplastic medulloblastomas and medullomyoblastomas: clinicopathological and genetic features.* J Neurosurg, 2001. **95**(1): p. 82-8.

48. Molloy, P.T., et al., *Central nervous system medulloepithelioma: a series of eight cases including two arising in the pons.* J Neurosurg, 1996. **84**(3): p. 430-6.

49. Yang, M., et al., *Primary atypical teratoid/rhabdoid tumor of central nervous system in children: a clinicopathological analysis and review of literature in China.* Int J Clin Exp Pathol, 2014. **7**(5): p. 2411-20.

50. Ebrahimi, A., et al., *ATRX immunostaining predicts IDH and H3F3A status in gliomas.* Acta Neuropathol Commun, 2016. **4**(1): p. 60.

51. Reuss, D.E., et al., *Adult IDH wild type astrocytomas biologically and clinically resolve into other tumor entities.* Acta Neuropathol, 2015. **130**(3): p. 407-17.

52. Reuss, D.E., et al., *IDH mutant diffuse and anaplastic astrocytomas have similar age at presentation and little difference in survival: a grading problem for WHO.* Acta Neuropathol, 2015. **129**(6): p. 867-73.

53. Reuss, D.E., et al., *ATRX and IDH1-R132H immunohistochemistry with subsequent copy number analysis and IDH sequencing as a basis for an "integrated" diagnostic approach for adult astrocytoma, oligodendroglioma and glioblastoma.* Acta Neuropathol, 2015. **129**(1): p. 133-46.

54. Chatterjee, D., et al., *IDH1, ATRX, and BRAFV600E mutation in astrocytic tumors and their significance in patient outcome in north Indian population.* Surg Neurol Int, 2018. **9**: p. 29.

55. Zanello, M., et al., *Clinical, Imaging, Histopathological and Molecular Characterization of Anaplastic Ganglioglioma.* J Neuropathol Exp Neurol, 2016. **75**(10): p. 971-980.

56. Singh, V., et al., *Rosette-forming and papillary glioneuronal tumors - A clinicopathological and molecular analysis.* Clin Neuropathol, 2019. **38**(4): p. 180-188.

57. Linsenmann, T., Monoranu, C., Alkonyi, B., Westermaier, T., Hagemann, C., Kessler, A., Ernestus, R., Lohr, M. , *Cerebellar Liponeurocytoma- molecular signature of a rare entity and the importance of an accurate diagnosis. .* Interdisciplinary Neurosurgery, 2019. **16**(16): p. 7-11.

58. Zhikrivetskaya, S.O., et al., *Molecular markers of paragangliomas/pheochromocytomas.* Oncotarget, 2017. **8**(15): p. 25756-25782.

59. Ishizawa, K., et al., *Olig2 and CD99 are useful negative markers for the diagnosis of brain tumors.* Clin Neuropathol, 2008. **27**(3): p. 118-28.

60. Amatya, V.J., et al., *Clinicopathological and immunohistochemical features of three pilomyxoid astrocytomas: comparative study with 11 pilocytic astrocytomas.* Pathol Int, 2009. **59**(2): p. 80-5.

61. Sharma, M.C., et al., *Subependymal giant cell astrocytoma--a clinicopathological study of 23 cases with special emphasis on histogenesis.* Pathol Oncol Res, 2004. **10**(4): p. 219-24.

62. Zakrzewska, M., et al., *Prevalence of mutated TP53 on cDNA (but not on DNA template) in pleomorphic xanthoastrocytoma with positive TP53 immunohistochemistry.* Cancer Genet Cytogenet, 2009. **193**(2): p. 93-7.

63. Bohara, M., et al., *Choroid Plexus Tumors: Experience of 10 Cases with Special References to Adult Cases.* Neurol Med Chir (Tokyo), 2015. **55**(12): p. 891-900.

64. Luyken, C., et al., *Supratentorial gangliogliomas: histopathologic grading and tumor recurrence in 184 patients with a median follow-up of 8 years.* Cancer, 2004. **101**(1): p. 146-55.

65. Gokden, M., *If it is Not a Glioblastoma, Then What is it? A Differential Diagnostic Review.* Adv Anat Pathol, 2017. **24**(6): p. 379-391.

66. Ertan, Y., et al., *Medulloblastoma: clinicopathologic evaluation of 42 pediatric cases.* Childs Nerv Syst, 2009. **25**(3): p. 353-6.

67. Venneti, S., et al., *p16INK4A and p14ARF tumor suppressor pathways are deregulated in malignant rhabdoid tumors.* J Neuropathol Exp Neurol, 2011. **70**(7): p. 596-609.

68. Mellai, M., et al., *IDH1 and IDH2 mutations, immunohistochemistry and associations in a series of brain tumors.* J Neurooncol, 2011. **105**(2): p. 345-57.

69. Thotakura, M., N. Tirumalasetti, and R. Krishna, *Role of Ki-67 labeling index as an adjunct to the histopathological diagnosis and grading of astrocytomas.* J Cancer Res Ther, 2014. **10**(3): p. 641-5.

70. Pradhan, P., et al., *Clinico-Histomorphological and Immunohistochemical Profile of Anaplastic Pleomorphic Xanthoastrocytoma: Report of Five Cases and Review of Literature.* Int J Hematol Oncol Stem Cell Res, 2018. **12**(4): p. 265-272.

71. Lamzabi, I., et al., *Immunophenotype of myxopapillary ependymomas.* Appl Immunohistochem Mol Morphol, 2013. **21**(6): p. 485-9.

72. Suri, V.S., et al., *Histological spectrum of ependymomas and correlation of p53 and Ki-67 expression with ependymoma grade and subtype.* Indian J Cancer, 2004. **41**(2): p. 66-71.

73. Nambirajan, A., et al., *A Comparative Immunohistochemical Study of Epithelial Membrane Antigen and NHERF1/EBP50 in the Diagnosis of Ependymomas.* Appl Immunohistochem Mol Morphol, 2018. **26**(1): p. 71-78.

74. Karamitopoulou, E., et al., *Ki-67 immunoreactivity in human central nervous system tumors: a study with MIB 1 monoclonal antibody on archival material.* Acta Neuropathol, 1994. **87**(1): p. 47-54.

75. Heim, S., et al., *Increased mitotic and proliferative activity are associated with worse prognosis in papillary tumors of the pineal region.* Am J Surg Pathol, 2014. **38**(1): p. 106-10.

76. Meurer, R.T., et al., *Immunohistochemical expression of markers Ki-67, neun, synaptophysin, p53 and HER2 in medulloblastoma and its correlation with clinicopathological parameters.* Arq Neuropsiquiatr, 2008. **66**(2B): p. 385-90.

77. Graham, D., et al., *Evaluation of Ki-67 reactivity in neuroblastoma using paraffin embedded tissue.* Pathol Res Pract, 1995. **191**(2): p. 87-91.

78. Lee, Y.S., T.W. Yeung, and O.C. Leung, *One of a kind-chordoid glioma in the fourth ventricle: a case report and literature review.* Acta Radiol Open, 2020. **9**(12): p. 2058460120980143.

79. Bandopadhayay, P., et al., *MYB-QKI rearrangements in angiocentric glioma drive tumorigenicity through a tripartite mechanism.* Nat Genet, 2016. **48**(3): p. 273-82.

80. Ligon, K.L., et al., *The oligodendroglial lineage marker OLIG2 is universally expressed in diffuse gliomas.* J Neuropathol Exp Neurol, 2004. **63**(5): p. 499-509.

81. Palsgrove, D.N., et al., *Subependymal giant cell astrocytoma-like astrocytoma: a neoplasm with a distinct phenotype and frequent neurofibromatosis type-1-association.* Mod Pathol, 2018. **31**(12): p. 1787-1800.

82. Taylor, M.D., et al., *Molecular subgroups of medulloblastoma: the current consensus.* Acta Neuropathol, 2012. **123**(4): p. 465-72.

83. Silva, T.M.E., et al., *Machine learning approaches reveal subtle differences in breathing and sleep fragmentation in Phox2b-derived astrocytes ablated mice.* J Neurophysiol, 2021.

84. Yi, F., et al., *Automatic extraction of cell nuclei from H&E-stained histopathological images.* J Med Imaging (Bellingham), 2017. **4**(2): p. 027502.

85. Sikpa, D., et al., *Automated detection and quantification of breast cancer brain metastases in an animal model using democratized machine learning tools.* Sci Rep, 2019. **9**(1): p. 17333.

86. Zeng, Z., et al., *RIC-Unet: An Improved Neural Network Based on Unet for Nuclei Segmentation in Histology Images.* IEEE Access, 2019. **7**: p. 21420-21428.

87. Ruifrok, A.C. and D.A. Johnston, *Quantification of histochemical staining by color deconvolution.* Anal Quant Cytol Histol, 2001. **23**(4): p. 291-9.

88. Otsu, N., *A Threshold Selection Method from Gray-Level Histograms.* IEEE Transactions on Systems, Man, and Cybernetics, 1979. **9**(1): p. 62-66.

89. Wu, H.S., J. Murray, and S. Morgello, *Segmentation of Brain Immunohistochemistry Images Using Clustering of Linear Centroids and Regional Shapes.* J Imaging Sci Technol, 2008. **52**(4): p. 405021-4050211.

90. Vazquez Landrove, M., *Consistency of Density Based Clustering and its Application to Image Segmentation*. 2018, George Mason University.

91. Arganda-Carreras, I., et al., *Trainable Weka Segmentation: a machine learning tool for microscopy pixel classification.* Bioinformatics, 2017. **33**(15): p. 2424-2426.

92. Ronneberger, O., P. Fischer, and T. Brox. *U-Net: Convolutional Networks for Biomedical Image Segmentation*. in *Medical Image Computing and Computer-Assisted Intervention – MICCAI 2015*. 2015. Cham: Springer International Publishing.

93. Abadi, M., et al., *TensorFlow: Large-Scale Machine Learning on Heterogeneous Distributed Systems.* ArXiv, 2016. **abs/1603.04467**.

94. Agarap, A.F., *Deep Learning using Rectified Linear Units (ReLU).* ArXiv, 2018. **abs/1803.08375**.

95. Zhang, Z. and M. Sabuncu. *Generalized Cross Entropy Loss for Training Deep Neural Networks with Noisy Labels*. in *NeurIPS*. 2018.

96. Karhohs, K. *Annotating Images with CellProfiler and GIMP*. 2018 [cited 2021; Available from: <carpenterlab.broadinstitute.org/blog/annotating-images-with-cellprofiler-and-gimp>.

**Supplementary Tables**

**Supplementary Table 1 (attached as an Xcel file): Impact of scrambling features on accuracy of random forest model.**

**Supplementary Table 2** Validation set data

| **Case** | **Diagnosis** | **Oligodendroglioma Probability** | **Age** | **p53_cont** | **ATRX** |
| --- | --- | --- | --- | --- | --- |
| Case 1 | Astrocytoma | 0.188 | 55 | 89 | mutation |
| Case 2 | Astrocytoma | 0.614 | 39 | 100 | no mutation |
| Case 3-1 | Oligodendroglioma | 0.744 | 40 | 1 | no mutation |
| Case 3-2 | Astrocytoma | 0.844 | 44 | 1 | no mutation |
| Case 4 | Astrocytoma | 0.922 | 63 | 2 | equivocal |
| Case 5 | Astrocytoma | 0.092 | 35 | 93 | mutation |
| Case 6 | Oligodendroglioma | 0.914 | 37 | 2 | no mutation |
| Case 7 | Astrocytoma | 0.22 | 26 | 100 | mutation |
| Case 8 | Oligodendroglioma | 0.63 | 31 | 0 | no mutation |
| Case 9 | Oligodendroglioma | 0.9 | 45 | 5 | no mutation |
| Case 10 | Astrocytoma | 0.52 | 33 | 0 | mutation |

**Figure legends**

**Fig. S1** Anaplastic Astrocytoma, WHO grade 2, IDH−mutated

**Fig. S2** Anaplastic Astrocytoma, WHO grade 2, IDH−wild type

**Fig. S3** Anaplastic Ependymoma, Posterior Fossa A, WHO grade 3

**Fig. S4** Anaplastic Ependymoma, Posterior Fossa B, WHO grade 3

**Fig. S5** Anaplastic Ependymoma, Spine, WHO grade 3

**Fig. S6** Anaplastic Ependymoma, Supratentorial−RELA, WHO grade 3

**Fig. S7** Anaplastic Ependymoma, Supratentorial−YAP, WHO grade 3

**Fig. S8** Anaplastic Ganglioglioma

**Fig. S9** Anaplastic Oligodendroglioma

**Fig. S10** Anaplastic Pleomorphic Xanthoastrocytoma

**Fig. S11** Angiocentric Glioma

**Fig. S12** Astroblastoma

**Fig. S13** Astrocytoma, WHO grade 2, IDH−Mutated

**Fig. S14** Astrocytoma, WHO grade 2, IDH−wild type

**Fig. S15** Atypical Choroid Plexus Papilloma, WHO grade 2

**Fig. S16** Atypical Rhabdoid Tumour

**Fig. S17** Central Neurocytoma

**Fig. S18** Cerebellar Liponeurocytoma, WHO grade 2

**Fig. S19** Choroid Glioma of The Third Ventricle

**Fig. S20** Choroid Plexus Carcinoma

**Fig. S21** Choroid Plexus Papilloma

**Fig. S22** Desmoplastic Infantile Astrocytoma, WHO grade 1

**Fig. S23** Desmoplastic Infantile Ganglioglioma

**Fig. S24** Diffuse Leptomeningeal Glioneuronal Tumor

**Fig. S25** Diffuse Midline Glioma, WHO grade 4

**Fig. S26** Diffuse Oligodendroglioma, WHO grade 2

**Fig. S27** Dysembryoplastic Neuroepithelial Tumor

**Fig. S28** Dysplastic Cerebellar Gangliocytoma, WHO grade 1

**Fig. S29** Ependymoma, Posterior Fossa A, WHO grade 2

**Fig. S30** Ependymoma, Posterior Fossa B, WHO grade 2

**Fig. S31** Ependymoma, Spine, WHO grade 2

**Fig. S32** Ependymoma, Supratentorial-YAP, WHO grade 2

**Fig. S33** Ependymoma, Supratentorial, RELA, WHO grade 2

**Fig. S34** Extraventricular Neurocytoma

**Fig. S35** Gangliocytoma, WHO grade 1

**Fig. S36** Glioblastoma

**Fig. S37** Astrocytoma, IDH−mutant, WHO grade 4

**Fig. S38** Glioblastoma, IDH−wild type, WHO grade 4

**Fig. S39** Medulloblastoma, non−WNT/non−SHH, WHO grade 4

**Fig. S40** Medulloblastoma, SHH Class, WHO grade 4

**Fig. S41** Medulloblastoma, WNT Group, WHO grade 4

**Fig. S42** Meningioma, WHO grade 1

**Fig. S43** Paraganglioma

**Fig. S44** Pilocytic Astrocytoma

**Fig. S45** Pilomyxoid Astrocytoma

**Fig. S46** Pleomorphic Xanthoastrocytoma, WHO grade 2

**Fig. S47** Rossete-forming Glioneuronl Tumour, WHO grade 1

**Fig. S48** Schwannoma, WHO grade 1

**Fig. S49** Solitary Fibrous Tumour/Hemangiopericytoma Grade 1

**Fig. S50** Subependymoma

**Fig. S51** Subependymal Giant Cell Astrocytoma, WHO grade 1

**Fig. S1-51. Summary of each entity in simulation model. a** Age distributions **b** Neuroanatomical distributions **c** Ki67 distributions **d** Distribution of histological features **e** Distribution of immunohistochemical features **f** Distribution of molecular pathology features. Data represented in panels D, E, and F are violin plots of the distribution of the histological, immunohistochemical, and molecular biomarkers. Each biomarker is listed as a binary readout of 0 = negative, 1 = positive. The violin shape provides an indication of the relative proportion showing positive or negative results for this biomarker. In cases where the literature does not support a distribution, (for instance, necrosis in astrocytoma, a notch is placed at the level of the 0).

**Fig. S52. Distribution of cases in the global IDH mutant (Grade 2 and 3) dataset.** World map shows the distribution of the cases in the global dataset. TCGA study (purple circles) has cases from different areas of the world such as Italy, Germany, Australia, but mostly the United States. This overall distribution of global dataset shows a good variety of ethnicity and genetic background to represent a real-world data.

**Fig. S53** **Deconvolution with Otsu’s thresholding and Watershed.** The images were splitted into R, G and B channels. Then R (Red), G (Green) and B (Blue) channels were converted to H (Hematoxylin), D (DAB) and X channels. D channel was selected for brown color in OLIG2 staining. After application of median filtering, Otsu's thresholding was applied to the D channel. The holes in the nuclei were filled out and then the nuclei on the border were eliminated for a better representation of nuclei. As a last step, watershed function was applied for overlapping or contacting nuclei.

**Fig. S54 K-means clustering segmentation.** The images were converted to a data frame using R, G and B pixel intensity values. All pixels were clustered into 3 clusters using k-means clustering method. Each pixel was labeled as Cluster 1, Cluster 2, or Cluster 3. The cluster that contain specifically the nuclei was chosen as foreground and the other 2 clusters were accepted as background.

**Fig. S55 Cut-Cluster-Classify (CCC) segmentation method. a** All patches in the image are viewed as high dimensional points and a threshold is applied to the sample density (Cut) to get clusters. After clustering (Cluster), the patches below the threshold were classified based on the clusters (Classify). **b** Then all pixels were voted for clusters based on cluster result on overlapping patches. The cluster that contains nuclei was accepted as foreground and the other one was accepted as background. 5x5 patches were used for the segmentation.

**Fig. S56 Relationships of R channel pixel intensity values with G and B pixel intensity values for color of noise.** Mean pixel intensity values of R channel are correlated to mean pixel intensity values of G and B channels. To better represent these spectrums, we used support vector machine (SVM) modeling to predict G and B values from R. The colors of noise are purple for H&E staining and brown for OLIG2 staining.

**Fig. S57 Image noise assay workflow and comparison of supervised and unsupervised image segmentation methods for H&E and OLIG2 immunostaining.** **a** An image noise assay was created for the fidelity evaluation of image segmentation methods to determine the best one for our purpose. We extracted the purple color for H&E staining and brown color for OLIG2 staining. A support vector model (SVM) was made to generate a noise similar to colors of the image. After generation of noise for a full image, the pixels were randomly sampled with certain percentages to implement the noise to the images. This workflow provided us to compare the performance of segmentation methods with different amount of noise. **b/c** We plotted segmentation performance by accuracy and intersection-over-union (IoU). Additionally, we added the U-Net trained with random noise generation as “U-Net with noise” to the comparison. In U-Net with noise, we trained a new model every time when we added noise to the image **b** In H&E staining, U-Net models show highest accuracies independent from the noise percentage. On the other hand, U-Net method shows low IoU, when U-Net with noise has a better IoU values. Overall deconvolution method has the better performance for accuracy and IoU. **c** In OLIG2 staining, similar to the H&E, deconvolution and U-Net with noise methods have the highest performance regarding accuracy and IoU. Despite deconvolution method has a sharp decrease after 25% noise, U-Net with noise maintained high IoU even after 25% noise.

**Fig S58. a** Gini importance of RF model for the probability of 1p19q codeletion using ATRX and age. **b** Gini importance of final RF model used for the 1p19q codeletion in validation data set. **c** Simulation of the model with different p53 percentages showing 1p19q codeletion probability in different scenarios.
