# Supplementary material for: Information theory approaches to improve glioma diagnostic workflows in surgical neuropathology
Source: Brain Pathol. 2022 Jan 10;32(5):e13050. doi: 10.1111/bpa.13050 (PMC9425010; doi:10.1111/bpa.13050)
Supplement: Supplementary file 4 — Supplementary Material [file BPA-32-e13050-s001.docx]

**GLOSSARY**

## Tumor GRADE

As in other subareas of anatomical pathology, we use common worldwide shared classifications of tumors in order to guide studies, treatments and prognostics of patients across different countries. This is established by the World Health Organization (WHO) in their “*blue book series*”. In this classification, each tumor type is assigned a roman number (I to IV) based on histological parameters (atypia, mitosis, necrosis and microvascular proliferation) which transmit somehow a bad or good prognosis. In general terms: grade I is benign (e.i. cured if resected), and grade II, III and IV are malign in an ascending fashion.

## CELL TYPE

Central nervous system is composed predominantly of nervous tissue, which cells include those of neuroectodermal origin: (a1) neurons, (a2) astrocytes, (a3) oligodendrocytes and (a4) ependymocytes; and cells of mesenchymal origin: (b1) meningeal cells, (b2) conjunctive tissue cells such as fibroblast, endothelium, adipose tissue, (b3) microglia.

In the peripheral nervous system, we don’t have oligodendrocytes but have a relative sibling called Schwann cell.

### Neurons

Neurons are cells diverse in size (small, intermediate, large), shape (rounded, pyramidal, etc), functions (inhibitory, excitatory) and structure (unipolar, pseudomonopolar, bipolar, multipolar). A prototypical pyramidal neuron is a triangular-shaped with rounded euchromatic nuclei (e.i. non-compacted chromatin) with prominent nucleoli. The cytoplasm is basophilic with dense bodies called “*Nissle substance*”, which represent highly packed rough endoplasmatic reticulum (ER). They have a variable number of afferent (e.i. inputs) processes called dendrities, and usually a single efferent process called axon.

There is some neuropathological related nomenclature referred to neurons such as

- ***Neurocytic***: they are small rounded cells, with sometimes a clear cytoplasm and rounded monotonous nuclei with fine chromatin. Prototype tumor: neurocytoma.
- ***Dysmorphic neuron***: this is like a neuron but not as well-formed that means their processes (dendrites and axon) are not well oriented, and their Nissel substance is lost. Prototype lesion: Focal Cortical Dysplasia type IIa.
- ***Ganglion cells***: they try to be like a neuron but without clear processes and Nissel substance. They have rounded nuclei and prominent nucleoli. Prototype tumor: ganglioglioma.

### Astrocytic glial cell

Being very simplistic, astrocytes can be thought of as “*sustentacular cells*”. They have a rounded-oval‌ ‌pale‌ ‌vesicular nucleus-quite small or non-prominent nucleoli. Cytoplasmic processes are not usually seen unless in reactive states, in which the eosinophilic cytoplasm forms short symmetric prolongations and the pedicels (“foot”) related to a vessel are more evident. There are some special “variants” which includes:

### Oligodendrocytes

^^[[1]](#footnote-1)^^Oligodendrocytes are responsible for the formation and maintenance of the myelin, which is a highly rich fatty acid substance that covers axons and allows normal electrical nerve impulse. They are small cells, with small rounded heterochromatic nuclei and no evident cytoplasm boundaries on H&E but evident with special techniques such as Golgi silver stain (right image). They are not infrequently seen near neurons, phenomena called satellitosis (not only seen in diffuse gliomas).

### Ependymocytes

This type of glial cell, which lines the ventricles and central canal of the spinal cord (hollow spaces in the brain filled with cerebrospinal fluid) are formed by a single layer of cuboidal cells with apical (e.i. surface) microvilli and cilia. Those localized in the third ventricle are considered specialized ependymal cells called **tanycytes**.

### Choroid plexus

They are responsible for producing the cerebrospinal fluid and can be thought as a folded membrane composed of a small amount of connective tissue (rich in blood vessels) lined with a simple cuboidal cell layer with rounded basal nuclei.

### Meningeal cells

Meningeal cells form part of the meninges, the “covers” of the brain. There are three meninges based on morphology and biophysical properties: the outer one that is very hard composed of fibrous tissue (duramater), the middle one has many vessels and was described as analogous to a spider web (arachnoid) , and the inner one that covers the smooth surface of the brain called pia mater. Meningeal cells are big-sized with eosinophilic cytoplasm which is difficult to discriminate between two adjacent cells (→ syncytial appearance)

### Schwann cells

Schwann cells main function is to produce myelin in the peripheral nervous system. It is a single cell whose membrane twists one or multiple times around a portion of an axon. Each axon is lined by multiple Schwann cells. It is characterized by a fusiform shape and ovoid nuclei.

### Mesenchymal cells

Mesenchymal cells are a heterogeneous designation is generally referred to those cells that reminds to any of the “less” differentiated cells of the connective tissue (immature mesenchymal cells such as those encountered in fetal/embryonal connective tissue) or to those that are similar to the prototypical cell of the mature connective tissue that is the fibroblast.

# OTHER CELLULAR/CYTOLOGICAL DESCRIPTIONS

## Rounded cells with a clear halo / oligo-like cells

This is an umbrella category that includes oligodendrogliomas, clear cell ependymomas, clear cell meningiomas, neurocytoma and some oligo-like areas of glioblastoma and pilocytic astrocytoma. They have in common that cells have an optic empty clear space (e.i. white space) between the nuclei and the extracellular matrix (e.i. the substance that surrounds the cells).

## Epithelioid cells

Epithelioid refers to cells that remind to epithelial cells in terms of shape (polygonal) and amount of eosinophilic cytoplasm (huge/moderate). Prototype tumor: Epithelioid glioblastoma.

## Rhabdoid cells

Rhabdoid cells are cells that somehow remind to the intermediate developmental stage of striated muscle. These are polygonal-shaped cells, with intense eosinophilic cytoplasm, and rounded nuclei with a prominent nucleolus. These cells are characteristic of the atypical teratoid/rhabdoid tumor (AT/RT).

## Lipidized cells

These cells are characterized by medium to big size, with foamy granular cytoplasm. They are frequently seen in Pleomorphic xanthoastrocytoma.

## Small round blue cells

This type of cells are defined by a high nuclear: cytoplasm relationships. This implies that they have hardly any cytoplasm and they are represented nearly all by their nuclei. Usually, they have a high mitotic index, fine chromatin pattern and extensive necrosis. Prototype tumors in this category are medulloblastomas, small cell neuroendocrine carcinomas and small cell glioblastomas.

# GLOBAL CELLULARITY

In pathology, cellularity refers to the relative amount of cells in a tissue, usually compared with their normal counterparts. As you can imagine, it is a very subjective observation. ***NUCLEAR PLEOMORPHISM***

Nuclear pleomorphism is another subjective descriptive term in pathology and refers to how different are nuclei in a population of study in terms of shape, chromatin pattern, etc. Usually (not always) there is a relationship between how terrible a nucleus looks like and their likelihood of malignancy. As in cellularity, they are grading it with words like mild, moderate or high pleomorphism based on “reference” values (e.i normal counterpart tissue).

#### Gigant multinucleated cells

As their name says, they are huge cells with multiple nuclei within their cytoplasm. They can have evenly shaped nuclei (as in tuberculosis reaction) or in some instances being very pleomorphic (called giant bizarre cells).

# GROWTH PATTERN

Based on the relationship of the tumor cells and the native parenchymal cells, growth pattern can be divided as

## Infiltrative diffuse pattern: In this pattern, tumoral cell intermingles with the native parenchyma making impossible to draw a line dividing normal from affected areas. Usually, it is possible to see a gradient of infiltrative cells. Prototype tumor: diffuse gliomas. In the image, there is a gradient of less cellular area (bottom left corner) to more cellular areas (upper right corner).

## Solid with infiltration: In this case, there is a distinction between tumor and native parenchyma but there are some cords, individual cells or islands that are in the advancing front of the tumor (ragged interface appearance). A prototypical example is a metastasis.

**Non-infiltrative** there is possible to distinguish a continuous line without irregularities between the tumors from the normal parenchyma. In some instances is also possible to resect the entire tumor without remnants. Although not in all cases, meningiomas can be an example of this category.

# ARCHITECTURAL PATTERN

## Nodular (Nests): Nodular/Nest pattern refers to masses of well-delimitated tumor cells with rounded contours.

## Solid discohesive/poorly cohesive sheets of cells

This pattern is characterized by they lose arrangement of cells without clear intercellular unions giving the appearance that cells are rejecting each other. Usually, they are seen in highly cellular tumors such as lymphoma or undifferentiated tumors.

## Cords/columns

In this pattern, cells tend to arrange in single rows (columns) or in more than one row (cords). This is typically seen in choroid glioma, (epithelial) metastasis, and in choroid meningioma.

## Chicken wire

In this pattern there is a prominent vascular network composed of small delicate anastomotic blood vessels (usual capillaries) that gives the impression of a chicken-wire, being capillaries the wire of the net. It is usually seen in endocrine tumors such as pheochromocytoma or in oligodendrogliomas.

## Pseudopapillar/Papillary

A **papilla** is defined as a central vascular fibro-conjunctive core lined by a variable amount and shape of cells. Meanwhile, **pseudopapilla is** a bunch of cells that reminds papillae but there is no real fibro-conjunctive core. When they are just a few clusters of cells (3-5 cells) that protrude into the lumen, they are called **micro-papilla**. A prototypal tumor in this category is Choroid Plexus Papilloma.

## Microcystic

This pattern shows multiple small to medium cystic spaces (e.i. open spaces) that can be empty or filled with variable content (mucin, glucogen, etc). It is frequently seen with the myxoid pattern described later.

## Myxoid

In this pattern cells are merged or floating within a rich extracellular matrix that reminds of an immature mesenchymal tissue because of the hypocellularity and somehow bluish colour. Usually, they are positive for special stains such as Alcian-Blue or Periodic Acid Schiff (PAS).

### Perivascular pseudorosettes

Perivascular pseudo-rosettes are characteristic of ependymomas. They are composed of a central small vessel, covered with a hyaline fibrillar intense eosinophilic area and a few layers of cells in the outermost space. Usually, those cells look quite similar to ependymocytes of the normal ventricular lining.

## Multilayered rosettes

It is quite similar to perivascular pseudo-rosettes but instead of having a few layers of cells covering the central vascular structure, they have multiple layers. This pattern is seen in the paradigmatic Embryonal tumors with multilayered rosettes.

## Storiform / Herringbone / Sarcomatous pattern

In this pattern cells usually have a fibroblastic-like appearance arranged in fascicles, laces, and trabeculae. Sometimes these fascicles are arranged in a way that resembles the bones of a fish (Herringbone-pattern) or fascicles radiate to form a central vortex like a whirlpool (storiform-pattern).

## Desmoplasia

Desmoplasia is defined as a growth of fibrous-like tissue caused by the accumulation of collagen fibers of various types (left image). Usually, it is highlighted with special stains such as reticulin stain (right image).

# SPECIAL FEATURES

## Rosenthal fibers

Rosenthal fibers are eosinophilic, corkscrew-like, thick fibers (looks like a thick pink rope). They can be seen in tumoral and non-tumoral lesions.

### Eosinophilic granular bodies

EGB are bright intense eosinophilic round bodies of variable size. They are usually seen in pilocytic astrocytomas and pleomorphic xanthoastrocytoma.

## Perivascular lymphocytes

In central nervous system immune cell entrance is highly regulated, so it is not expected to see a great amount of lymphoid tissue in normal conditions. In some conditions lymphocytes (small cells with hyperchromatic nuclei and scarcely basophilic cytoplasm) tend to wrap vascular structures giving the **perivascular** designation.

## Calcifications

Calcifications are seen in H&E as variable size rounded basophilic bodies (left image). Sometimes they are composed of concentric layers and are called **psammoma bodies**.

## Endothelial proliferation / Microvascular proliferation

Endothelial proliferation is defined as an augment in blood vessels numbers with more than one endothelial lining internal cell layer, compared with their normal counterpart that have only one single layer. When there is a stinking proliferation, that forms rounded grape-like structures reminiscent of renal glomeruli, they are called **glomerular endothelial proliferation**. Usually, they are seen in high-grade gliomas but can be also seen in low-grade gliomas too.

### Necrosis

Necrosis is the premature death of cells caused by an irreversible injury. There are many morphological patterns of necrosis (coagulative, liquefactive, caseous, fat, etc). Coagulative necrosis occurs as a result of hypoxic states (such as those seen in tumors that grow faster than their capability of nourish). They are seen in H&E sections as eosinophil areas with “**ghost-cells**” (cells that are morphologically reminiscent of a cell, but without the basophilic nuclei that was lost). With time, these dead cells tend to break up, leaving a “dirty” material (similar to cracked earth).

1. Took from <http://vanat.cvm.umn.edu/neurLab1/glia.html> [↑](#footnote-ref-1)
